# Supplementary figures and images for: Loss of hepatic aldolase B activates Akt and promotes hepatocellular carcinogenesis by destabilizing the Aldob/Akt/PP2A protein complex
Source: PLoS Biol. 2020 Dec 4;18(12):e3000803. doi: 10.1371/journal.pbio.3000803 (PMC7744066; doi:10.1371/journal.pbio.3000803)

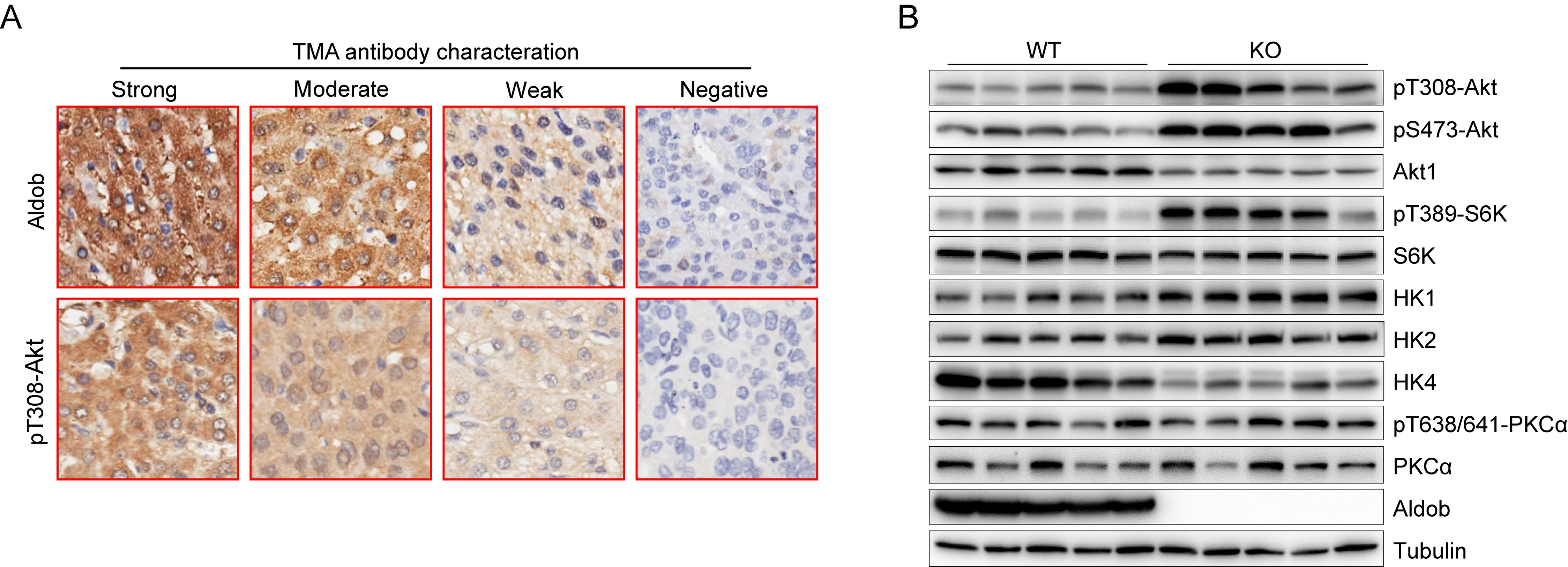

Supplement: S1 Fig — (A) TMA antibody characterization of Aldob and pT308-Akt (original magnification ×200). (B) IB analysis of WCL derived from liver tumor tissues of WT and ALDOB KO mice after injection with DEN at postnatal day 14 to induce hepatocellular carcinoma for 10 months. The data underlying this figure can be found in S2 Data. Aldob, aldolase B; DEN, diethyl nitrosamine; HCC, hepatocellular carcinoma; IB, immunoblot; KO, knockout; mTORC1, mechanistic target of rapamycin complex 1; TMA, tissue microarray; mTORC2, mechanistic target of rapamycin complex 2; WCL, whole cell lysate. (TIF) [file pbio.3000803.s001.tif]

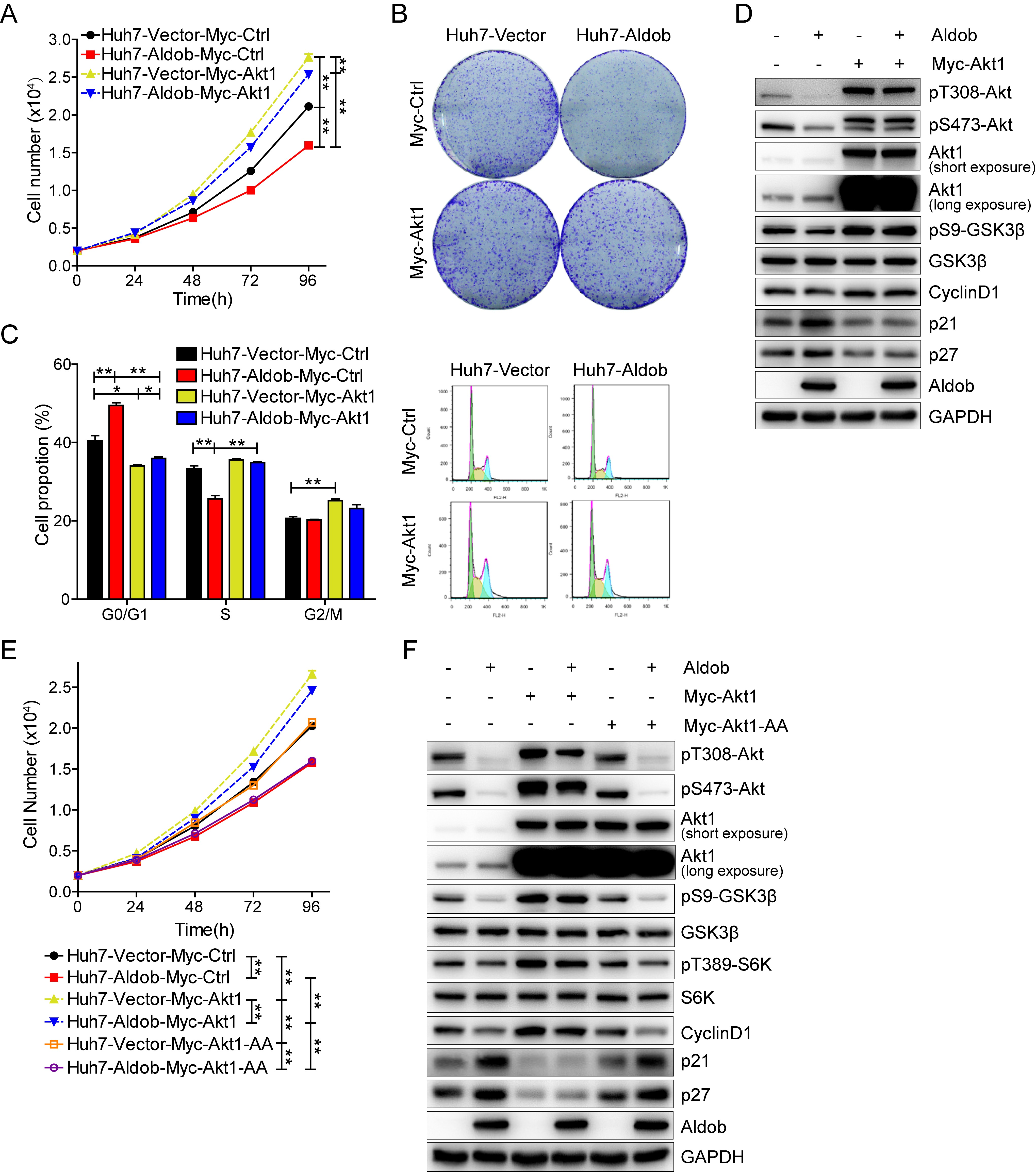

Supplement: S2 Fig — (A) Cell viability assay of Huh7-Vector and Huh7-Aldob cells transfected with Myc-Ctrl or Myc-Akt1 constructs. (B) Representative graphs from colony formation assay of Huh7-Vector and Huh7-Aldob cells after transfection with Myc-Ctrl or Myc-Akt1 for 7 days. (C and D) Cells were transfected with Myc-Ctrl or Myc-Akt1 for 48 hours, and then monitored for cell cycle distribution (C) or subjected to IB analysis (D). (E) Cell viability assay of Huh7-Vector and Huh7-Aldob cells transfected with the indicated constructs. Myc-Akt1-AA indicated the Myc-Akt1 phospho-deficient mutant harboring duple mutations (T308A/S473A). (F) IB analysis of WCL derived from Huh7 cells transfected with the indicated constructs. Data are presented as mean ± SEM. * p < 0.05; ** p < 0.01 (Student t test). The data underlying this figure can be found in S2 Data. Aldob, aldolase B; IB, immunoblot; WCL, whole cell lysate. (TIF) [file pbio.3000803.s002.tif]

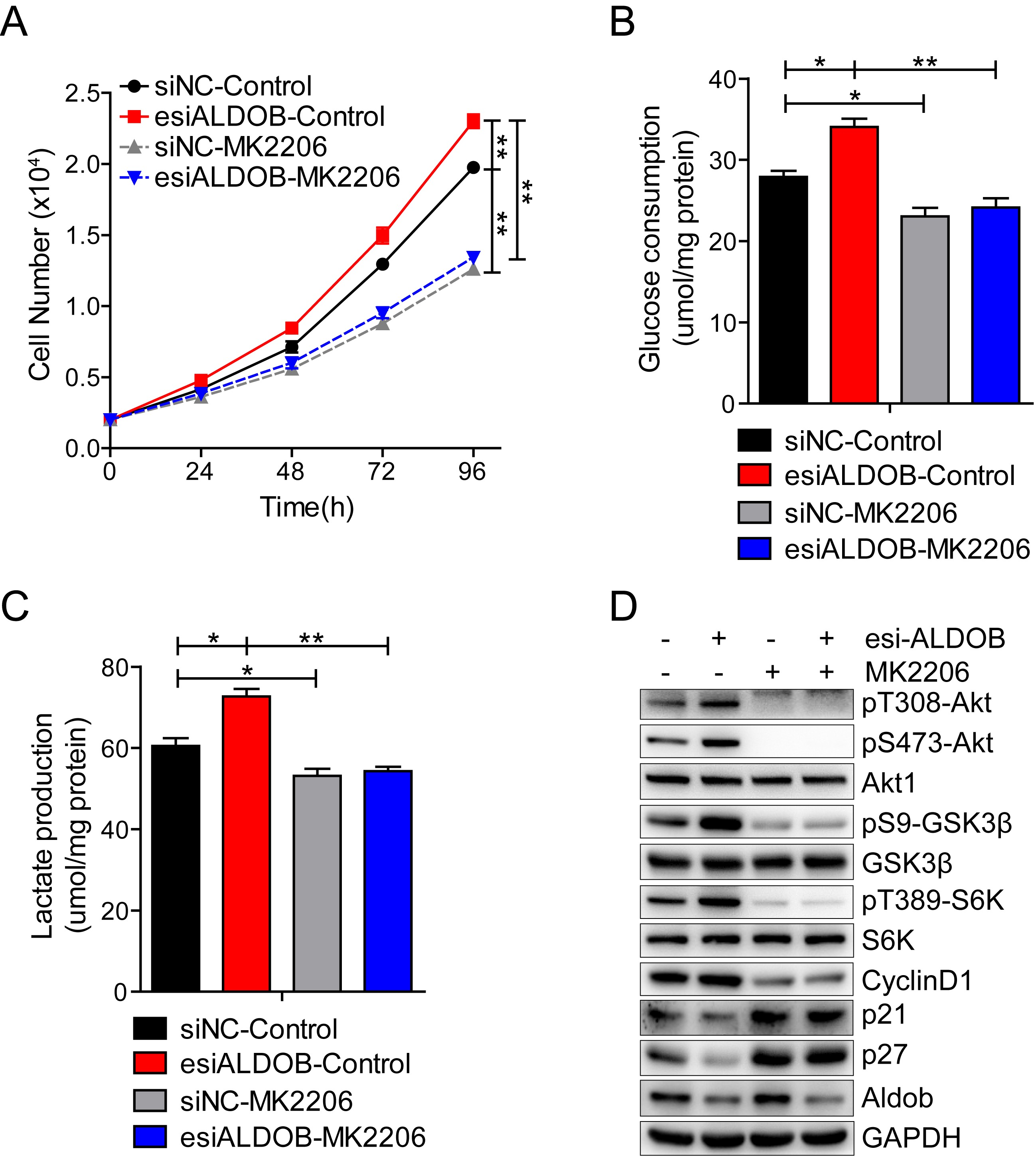

Supplement: S3 Fig — (A) Cell viability of Huh7 cells transfected with indicated siRNAs in the presence of DMSO or MK2206 (2 μM). (B and C) Glucose consumption (B) and lactate production (C) of Huh7 cells transfected with indicated siRNAs after treatment with DMSO or MK2206 (5 μM). (D) IB analysis of WCL derived from Huh7 cells transfected with indicated siRNAs in the presence of DMSO or MK2206 (2 μM). Data are presented as mean ± SEM. * p < 0.05; ** p < 0.01 (Student t test). The data underlying this figure can be found in S2 Data. Aldob, aldolase B; IB, immunoblot; siRNA, small interfering RNA; WCL, whole cell lysate. (TIF) [file pbio.3000803.s003.tif]

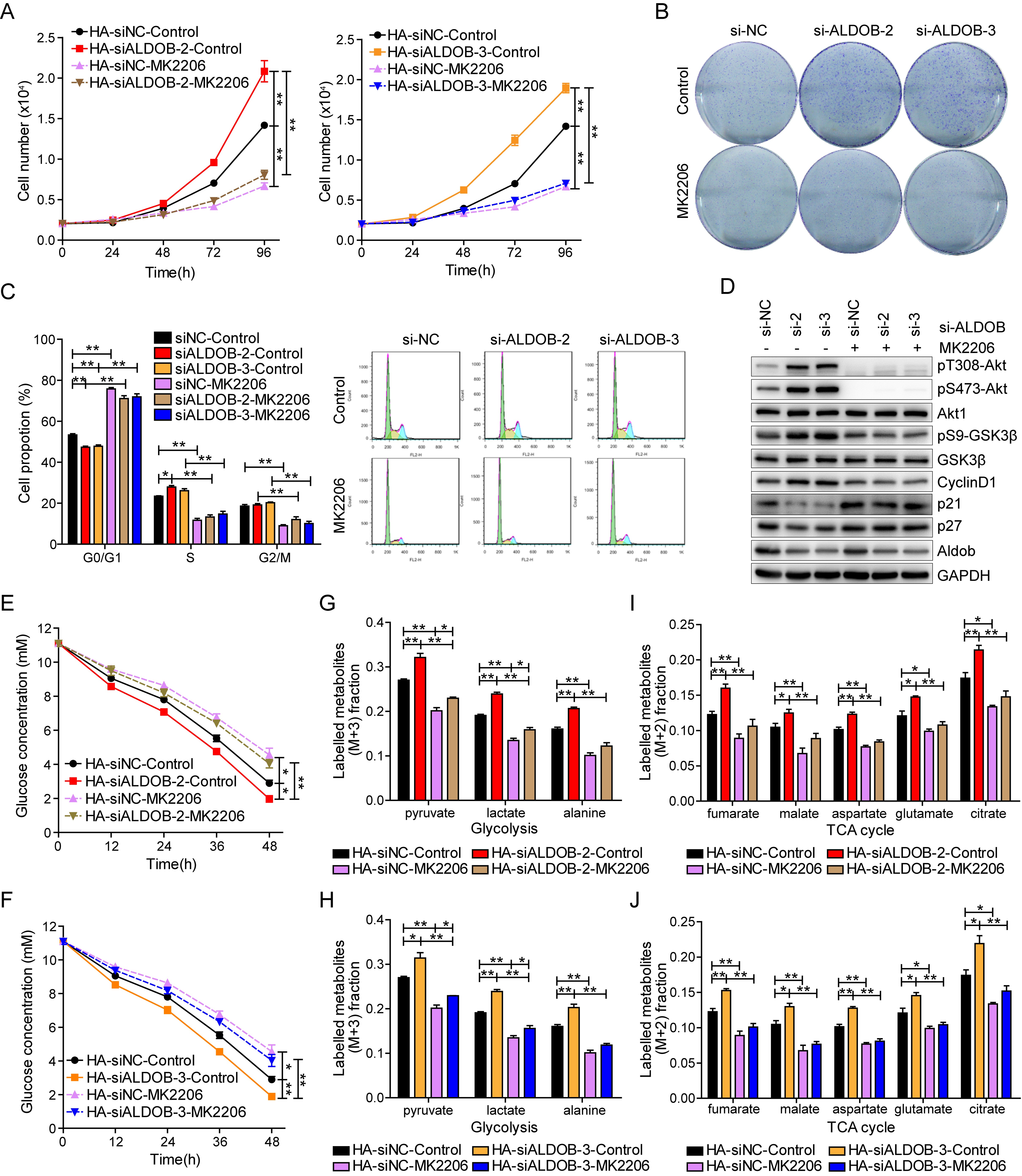

Supplement: S4 Fig — (A–D) Aldob-overexpressing Huh7 cells transfected with indicated siRNAs were used to determine their effects with or without MK2206 treatment (2 μM) on cell proliferation (A), colony formation (B), cell cycle distribution (C), and cell cycle–related protein levels (D). (E and F) Glucose levels in the culture medium of Huh7-Aldob cells transfected with indicated siRNAs after treatment with DMSO or MK2206 (5 μM) at different time points. (G and H) Fraction of the labeled metabolites of M+3 from 13C-glucose in glycolysis by DMSO or MK2206 (5 μM) treatment for 12 hours in Huh7-Aldob cells transfected with indicated siRNAs. (I and J) Fraction of the labeled metabolites of M+2 from 13C-glucose in TCA cycle by DMSO or MK2206 (5 μM) treatment for 12 hours in Huh7-Aldob cells transfected with indicated siRNAs. Data are presented as mean ± SEM. * p < 0.05; ** p < 0.01 (Student t test). The data underlying this figure can be found in S2 Data. Aldob, aldolase B; siRNA, small interfering RNA; TCA, tricarboxylic acid. (TIF) [file pbio.3000803.s004.tif]

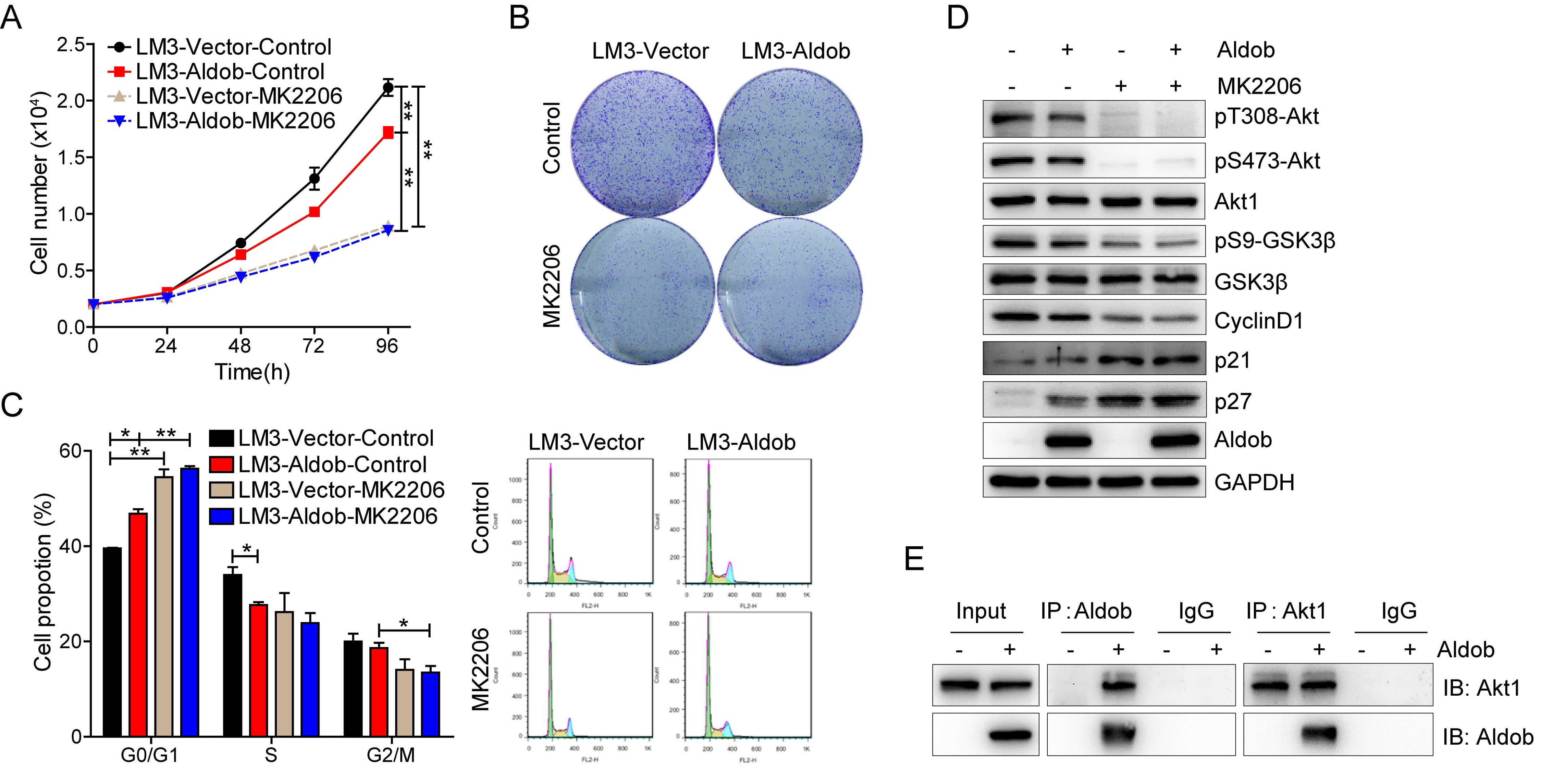

Supplement: S5 Fig — (A–D) LM3 cells stably expressing Aldob via lentiviral infection (with Vector as a negative control) were used to examine their biological functions in the presence of either control DMSO or MK2206 (2 μM), including cell proliferation (A), colony formation (B), cell cycle distribution (C), and the protein levels of Akt pathway (D). (E) Co-IP analysis to demonstrate the interaction between exogenous Aldob and endogenous Akt1 in LM3-Aldob cells. Data are presented as mean ± SEM. * p < 0.05; ** p < 0.01 (Student t test). The data underlying this figure can be found in S2 Data. Aldob, aldolase B; HCC, hepatocellular carcinoma; IP, immunoprecipitation. (TIF) [file pbio.3000803.s005.tif]

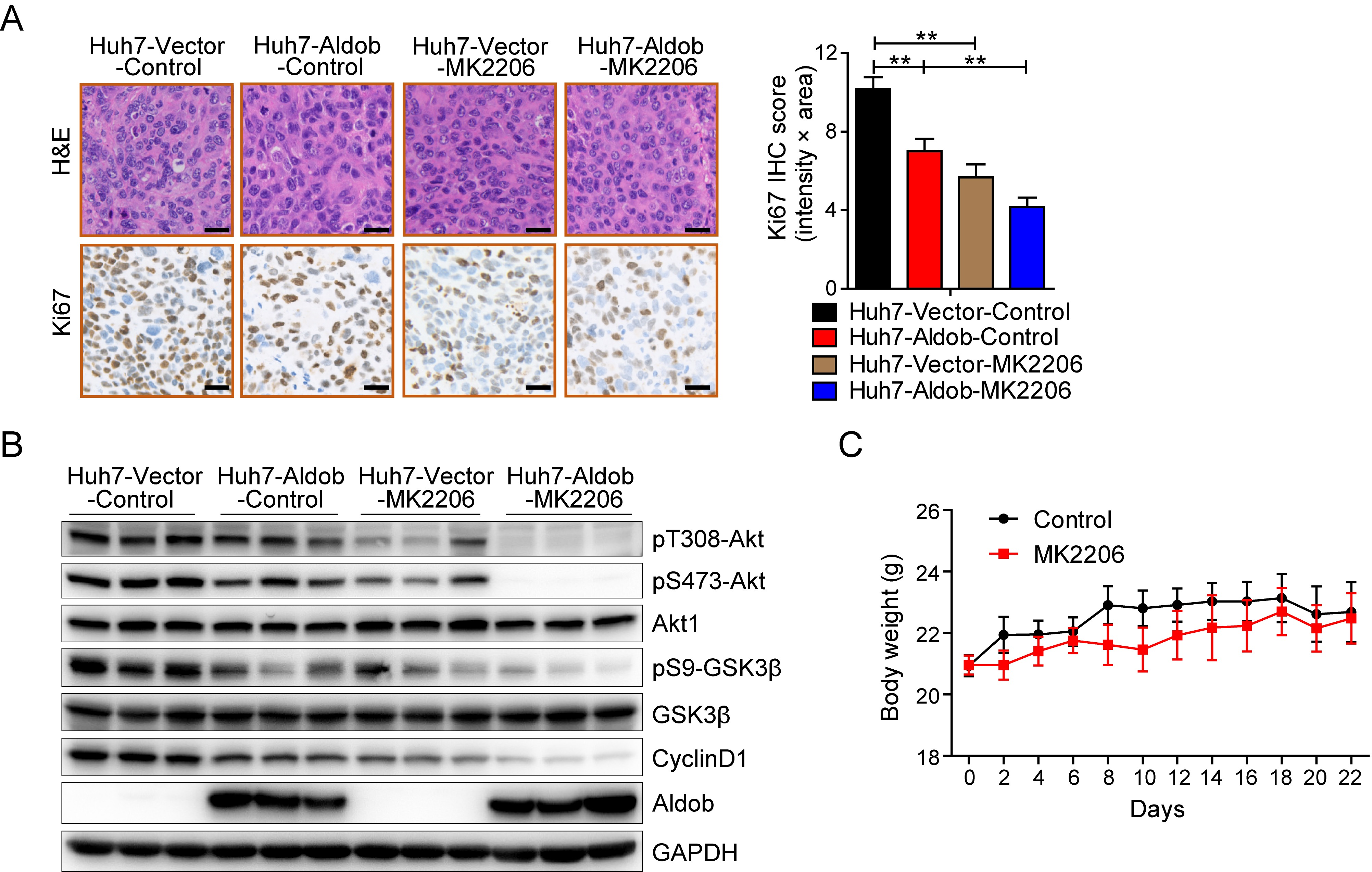

Supplement: S6 Fig — (A) Representative IHC images and quantification of Ki67 expression in Huh7-Vector and Huh7-Aldob xenograft tumors treated with control solvent or MK2206 (n = 6). Scale bars, 50 μm. (B) IB analysis of WCL derived from Huh7-Vector and Huh7-Aldob tumors treated with control solvent or MK2206. (C) The body weights of mice in Fig 3I were recorded. Data are presented as mean ± SEM. * p < 0.05; ** p < 0.01 (Student t test). The data underlying this figure can be found in S2 Data. Aldob, aldolase B; IHC, immunohistochemistry; WCL, whole cell lysate. (TIF) [file pbio.3000803.s006.tif]

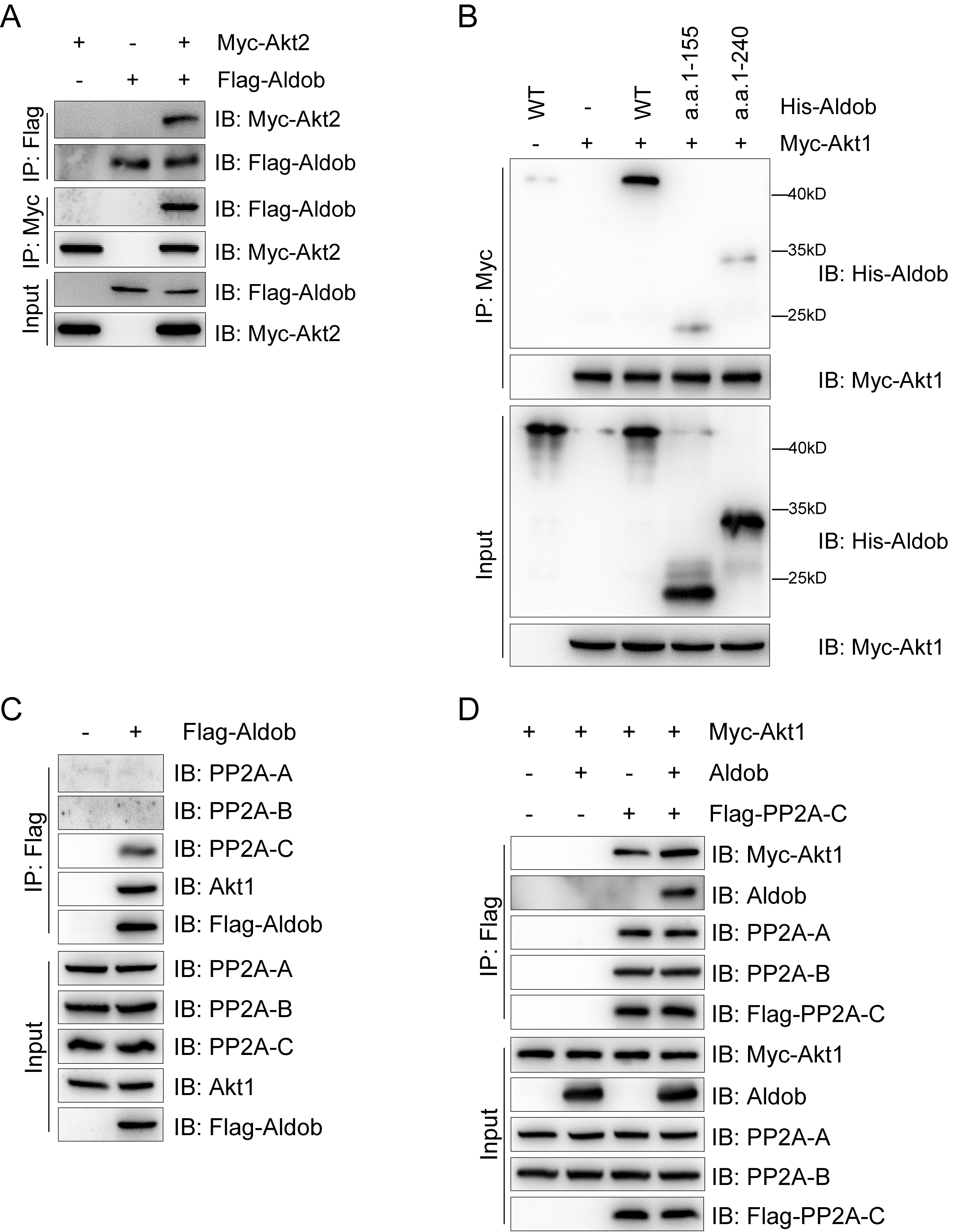

Supplement: S7 Fig — (A) Co-IP assay to show that Akt2 interacted with Aldob in Huh7 cells at ectopic expression conditions. (B) IP analysis was performed with WCL derived from Myc-Akt1 transfected Huh7 cells and various truncated mutants of recombinant His-Aldob proteins to illustrate that the carboxyl-terminal region (a.a. 241–364) of Aldob is responsible for Akt1-binding. (C and D) IB analysis of Flag-IP and WCL derived from Huh7 cells transfected with the indicated constructs. The data underlying this figure can be found in S2 Data. Aldob, aldolase B; IP, immunoprecipitation; PP2A-C, the catalytic subunit of protein phosphatase 2A; WCL, whole cell lysate. (TIF) [file pbio.3000803.s007.tif]

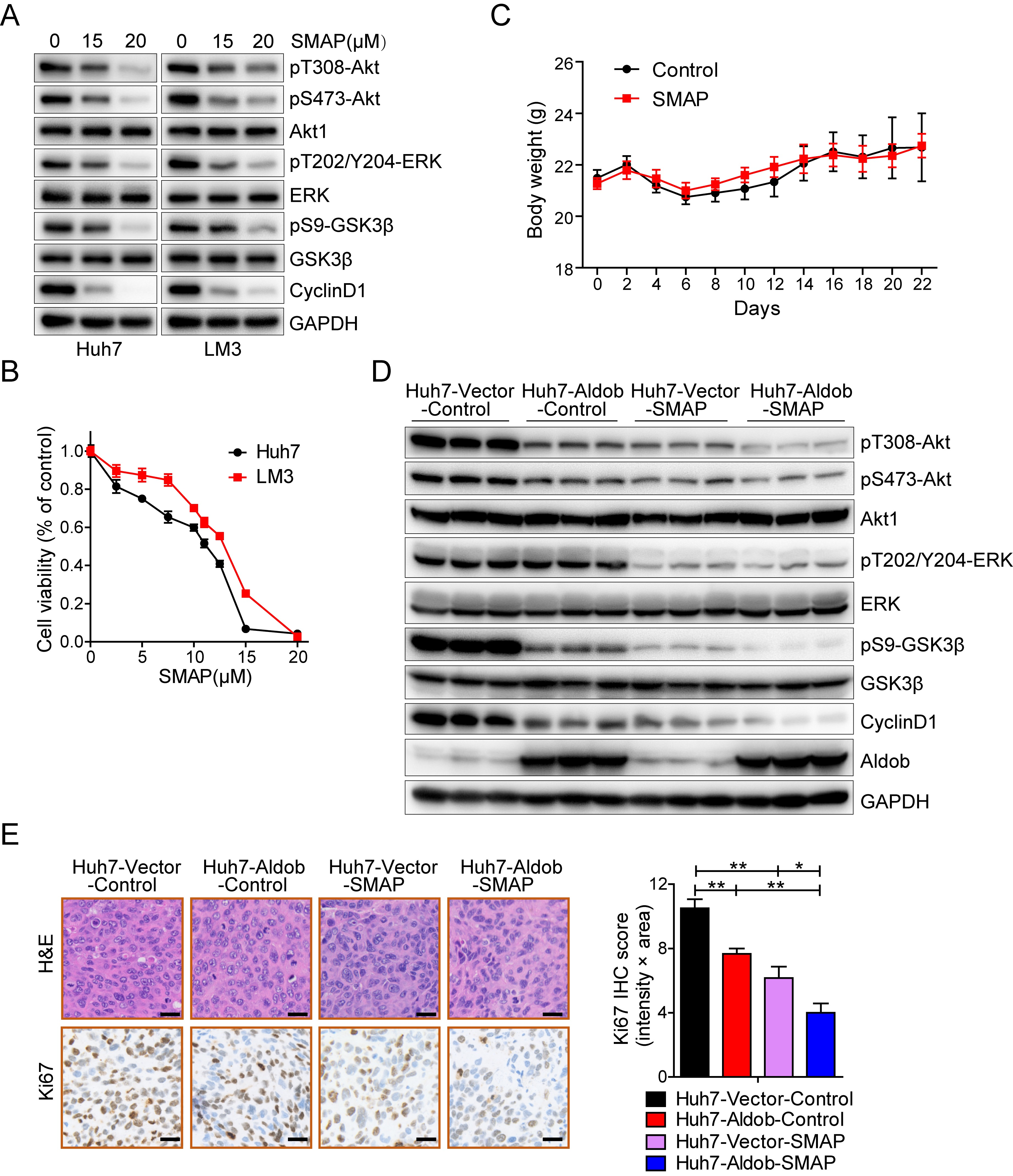

Supplement: S8 Fig — (A) IB analysis of WCL derived from Huh7 and LM3 cells treated with SMAP for 24 hours at the indicated concentrations. (B) Relative cell viability of Huh7 and LM3 cells treated with increasing concentrations of SMAP for 48 hours. (C) The body weights of mice in Fig 7I were recorded. (D) IB analysis of WCL derived from Huh7-Vector and Huh7-Aldob tumors treated with control solvent or SMAP. (E) Representative IHC images and quantification of Ki67 expression in Huh7-Vector and Huh7-Aldob xenografts treated with control solvent or SMAP (n = 6). Scale bars, 50 μm. Data are presented as mean ± SEM. * p < 0.05; ** p < 0.01 (Student t test). The data underlying this figure can be found in S2 Data. Aldob, aldolase B; HCC, hepatocellular carcinoma; IB, immunoblot; IHC, immunohistochemistry; PP2A, protein phosphatase 2A; SMAP, small-molecule activator of PP2A; WCL, whole cell lysate. (TIF) [file pbio.3000803.s008.tif]
