# Supplementary material for: Loss of hepatic aldolase B activates Akt and promotes hepatocellular carcinogenesis by destabilizing the Aldob/Akt/PP2A protein complex
Source: PLoS Biol. 2020 Dec 4;18(12):e3000803. doi: 10.1371/journal.pbio.3000803 (PMC7744066; doi:10.1371/journal.pbio.3000803)
Supplement: S1 Table — (DOCX) [file pbio.3000803.s009.docx]

S1 Table. Association of clinicopathological characteristics with Aldob and p-Akt expression in HCC patients (n=70).

| Variable | **Aldob (total n=70)** | |  | | **pT308-Akt (total n=70)** | |  | |
| --- | --- | --- | --- | --- | --- | --- | --- | --- |
|  | Low expression (n=34) | High expression (n=36) | | p | Low expression (n=38) | High expression (n=32) | | p |
| Background |  |  | |  |  |  | |  |
| Male | 32 (94.1%) | 35 (97.2%) | | .522 | 37 (97.4%) | 30 (93.8%) | | .456 |
| Age (y) | 46.0 ± 8.4 | 47.0 ± 10.0 | | .652 | 46.0 ± 9.5 | 47.1 ± 9.0 | | .161 |
| HBsAg | 33 (97.1%) | 34 (94.4%) | | .589 | 37 (97.4%) | 30 (93.8%) | | .456 |
| HBeAg | 15 (44.1%) | 11 (30.6%) | | .241 | 14 (36.8%) | 12 (37.5%) | | .955 |
| Anti-HBcAg | 33 (97.1%) | 35 (97.2%) | | .967 | 36 (94.7%) | 32 (100.0%) | | .188 |
| HBV-DNA (× 10^4^/mL) | 15.5 (0.0, 32000.0) | 12.5 (0.0, 2500.0) | | .199 | 14.0 (0.0, 3200.0) | 11.5 (0.0, 1400.0) | | .478 |
| Liver cirrhosis | 34 (100.0%) | 35 (97.2%) | | .328 | 37 (97.4%) | 32 (100.0%) | | .355 |
| AFP (ng/mL) | 1067.4 (1.8, 1210.0) | 54.2 (2.0, 1210.0) | | .000* | 230.8 (1.8, 1210.0) | 292.7 (2.7, 1210.0) | | .580 |
| Liver function |  |  | |  |  |  | |  |
| ALT (IU/L) | 42.5 (18.0, 145.0) | 41.0 (17.0, 320.0) | | .370 | 42.5 (19.0, 320.0) | 39.5 (17.0, 83.0) | | .203 |
| AST (IU/L) | 42.5 (19.0, 160.0) | 35.5 (20.0, 172.0) | | .452 | 39.5 (19.0, 172.0) | 40.5 (20.0, 120.0) | | .326 |
| Total bilirubin (μmol/L) | 13.4 (5.3, 626.5) | 12.2 (5.4, 384.1) | | .961 | 12.3 (5.3, 626.5) | 14.6 (6.4, 384.1) | | .651 |
| Albumin (g/L) | 41.3 (34.6, 49.3) | 43.0 (37.1, 53.4) | | .036* | 41.2 (34.6, 53.4) | 42.8 (34.9, 49.8) | | .356 |
| PT (s) | 12.1 (10.8, 15.3) | 12.1 (10.4, 15.3) | | .156 | 12.3 (11.0, 15.3) | 11.8 (10.4, 13.3) | | .001* |
| Tumor Factors |  |  | |  |  |  | |  |
| Tumor size (cm) | 7.5 (1.0, 17.0) | 6.0 (2.0, 13.0) | | .166 | 6.0 (1.0, 17.0) | 7.5 (2.0, 15.0) | | .507 |
| No. tumor | 29 (85.3%) | 32 (88.9%) | | .579 | 37 (94.7%) | 25 (78.1%) | | .039* |
| Macro-vascular invasion | 4 (11.8%) | 6 (16.7%) | | .558 | 5 (13.2%) | 5 (15.6%) | | .769 |
| Pathologic factors |  |  | |  |  |  | |  |
| Tumor capsule | 7 (20.6%) | 12 (61.1%) | | .001* | 20 (52.6%) | 9 (28.1%) | | .038* |
| Micro-vascular invasion | 18 (52.9%) | 12 (33.3%) | | .098 | 17 (44.7%) | 13 (40.6%) | | .729 |
| Differentiation (III) | 29 (85.3%) | 24 (66.7%) | | .069 | 28 (73.7%) | 25 (78.1%) | | .666 |
| Recurrence (month) | 6.7 (1.0, 35.0) | 15.8 (1.0, 35.6) | | .038* | 15.1 (1.0, 35.6) | 5.7 (1.0, 35.2) | | .236 |
| Overall survival (month) | 20.8 (1.3, 35.6) | 26.7 (5.1, 36.3) | | .016* | 25.7 (5.1, 36.3) | 19.8 (1.3, 35.2) | | .029* |

AFP: Alpha-fetoprotein. ALT: Alanine aminotransferase. AST: Aspartate aminotransferase. PT: Prothrombin time. Aldob and p-Akt expression were defined as staining intensity. Protein expression intensity was classified into negative, weak, moderate, strong. The score calculation formula: (weak + moderate + strong)/ (negative + weak + moderate + strong) × 100%. Aldob and p-Akt expression score >50% are high, expression score <50% are low. * p <0.05 by χ2 test.
