# Supplementary material for: Loss of hepatic aldolase B activates Akt and promotes hepatocellular carcinogenesis by destabilizing the Aldob/Akt/PP2A protein complex
Source: PLoS Biol. 2020 Dec 4;18(12):e3000803. doi: 10.1371/journal.pbio.3000803 (PMC7744066; doi:10.1371/journal.pbio.3000803)
Supplement: S1 Raw Images — (PDF) [file pbio.3000803.s012.pdf]

Raw Images of Western blot

Main Figures

Fig 1A

Left panel

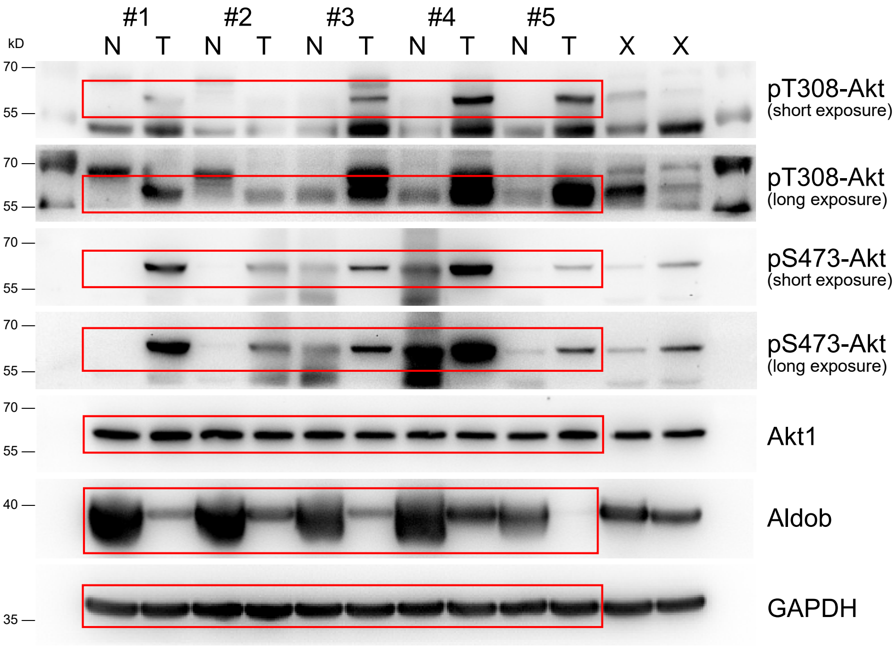

Right panel

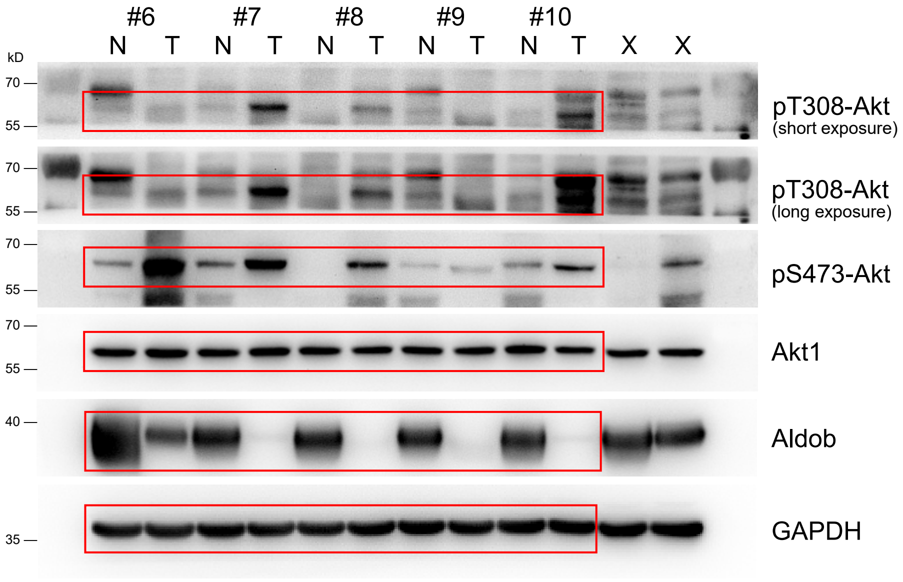

1. Images were acquired by chemiluminescence CCD camera-based digital imaging instruments (Tanon 5200 Chemiluminescent Imaging System).
2. Approx. molecular weight ladder was indicated.
3. PVDF Membranes were cut for immunoblotting of more than one protein.

Fig 2A

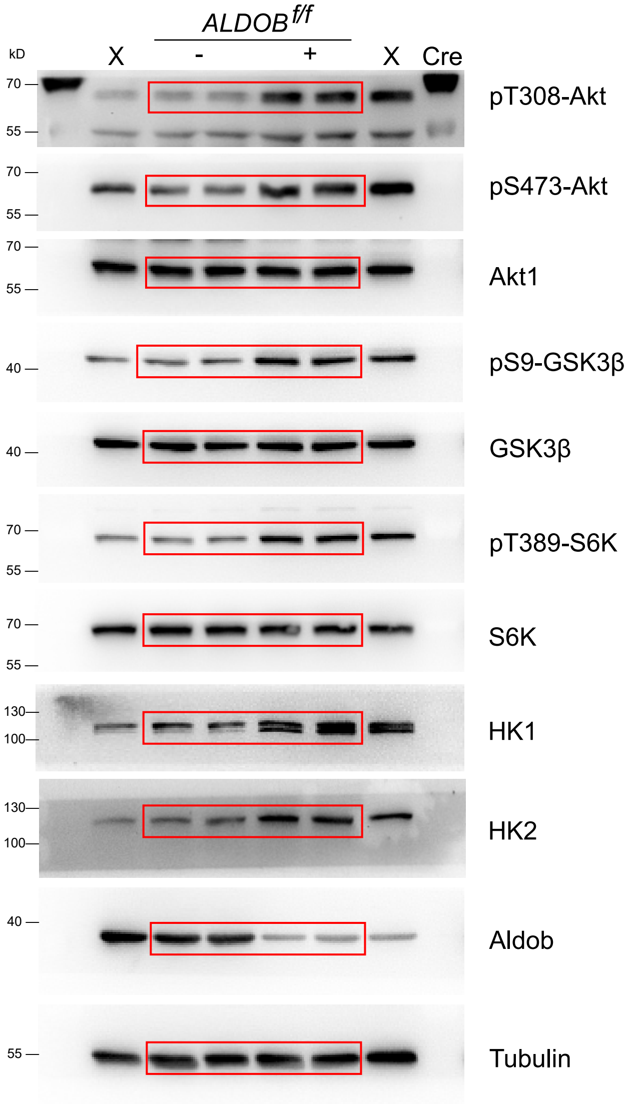

Fig 2B

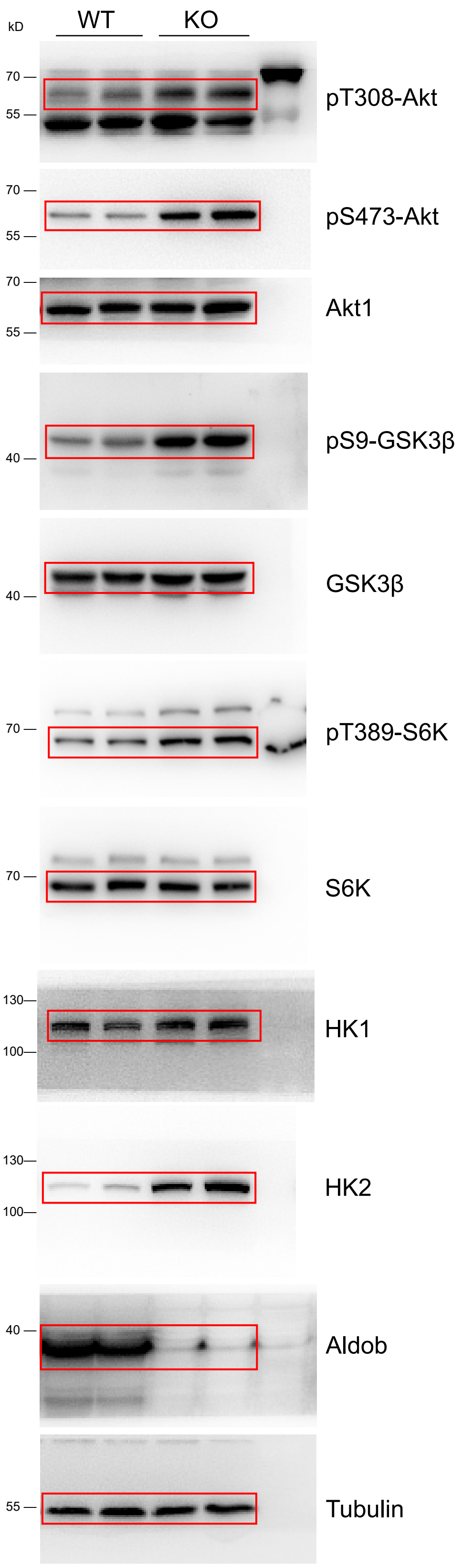

1. Images were acquired by chemiluminescence CCD camera-based digital imaging instruments (Tanon 5200 Chemiluminescent Imaging System).
2. Approx. molecular weight ladder was indicated.
3. PVDF Membranes were cut for immunoblotting of more than one protein.

Fig 2C

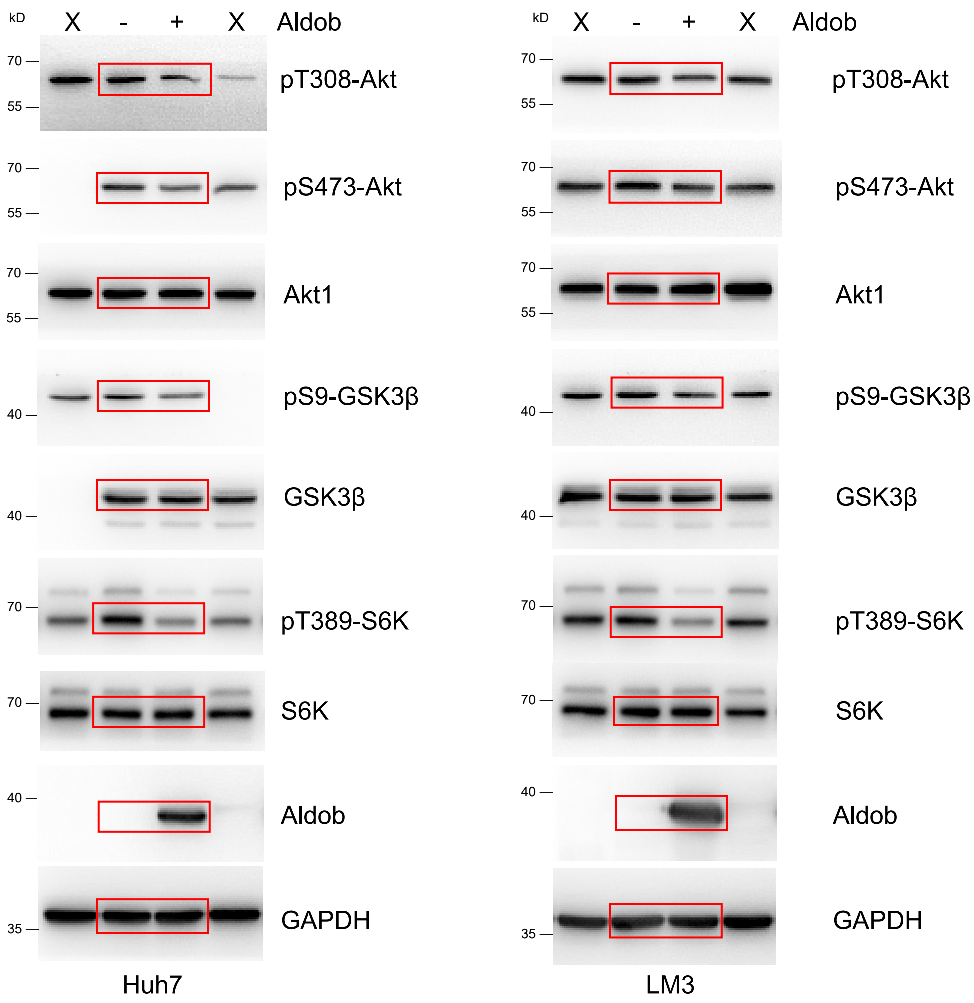

Fig 2D

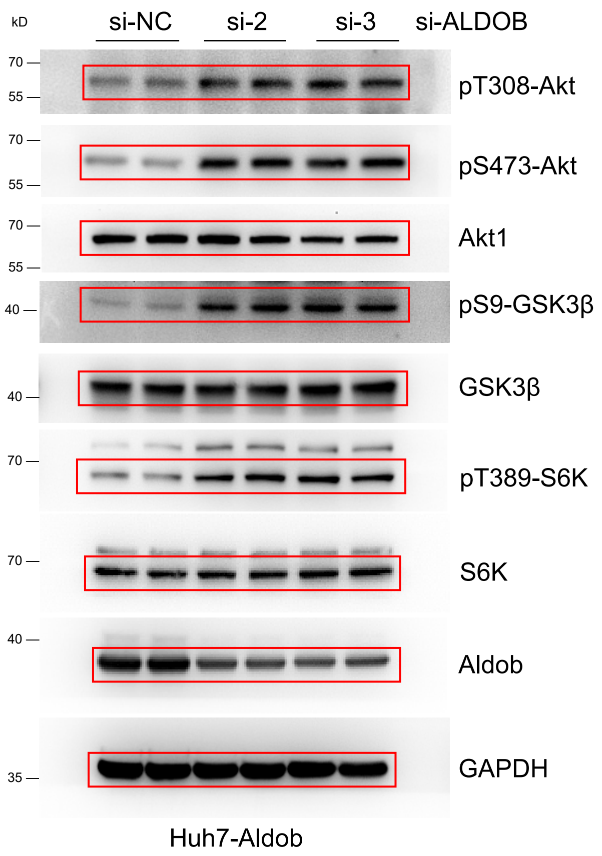

1. Images were acquired by chemiluminescence CCD camera-based digital imaging instruments (Tanon 5200 Chemiluminescent Imaging System).
2. Approx. molecular weight ladder was indicated.
3. PVDF Membranes were cut for immunoblotting of more than one protein.

Fig 2E

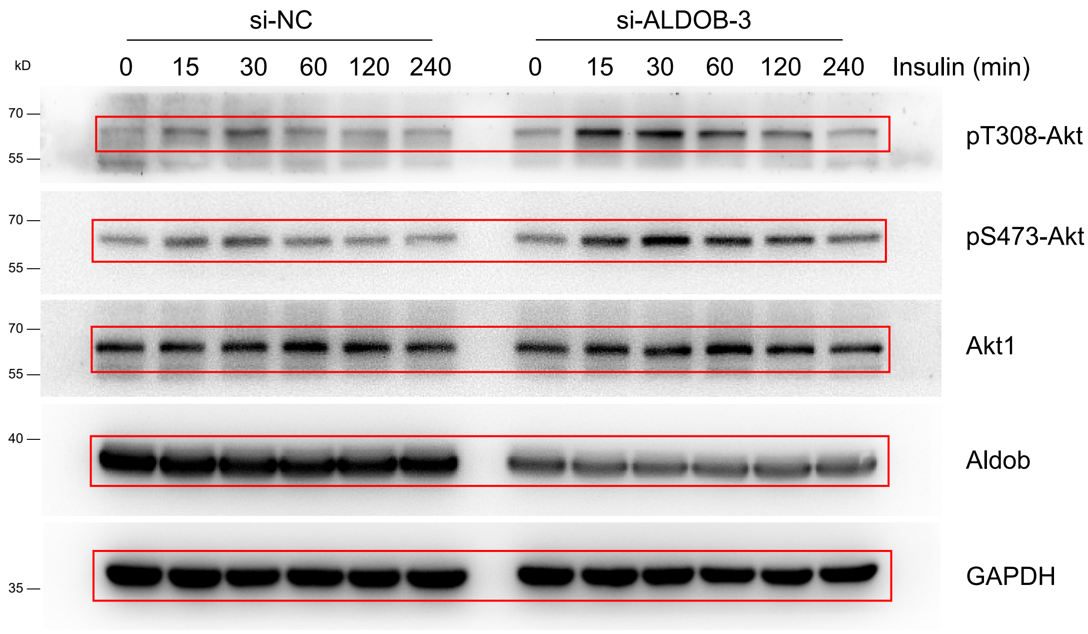

Fig 2F

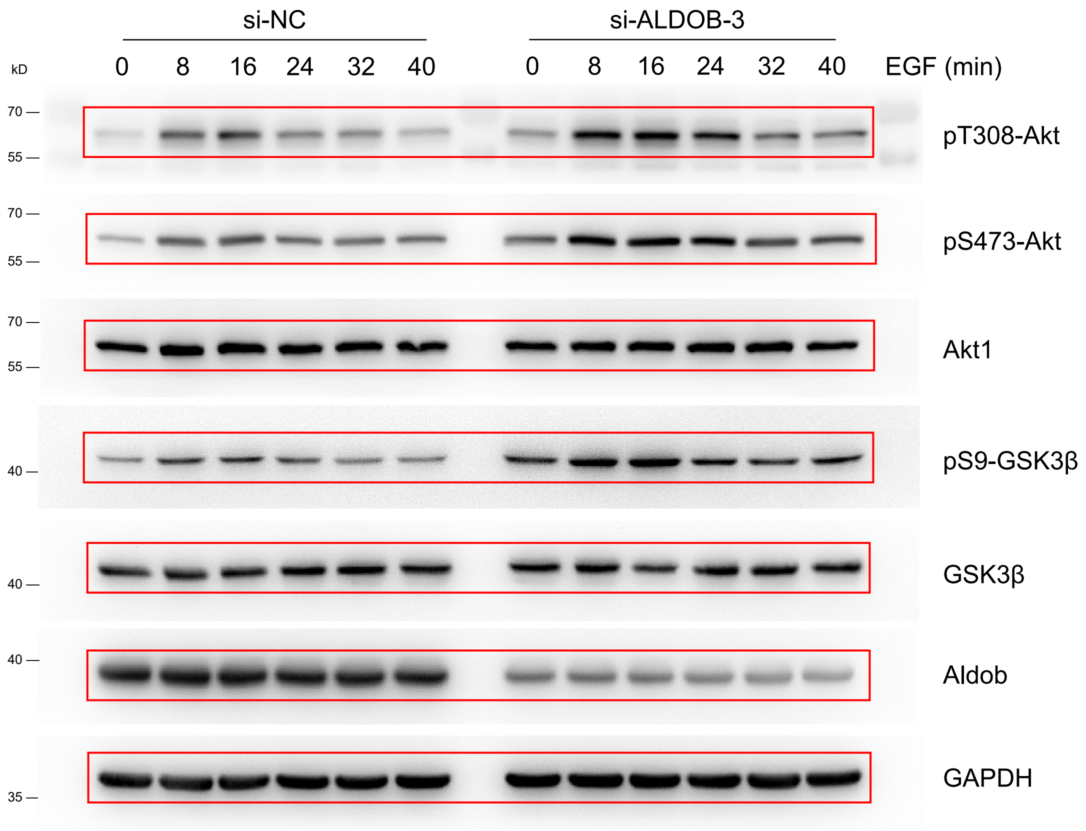

1. Images were acquired by chemiluminescence CCD camera-based digital imaging instruments (Tanon 5200 Chemiluminescent Imaging System).
2. Approx. molecular weight ladder was indicated.
3. PVDF Membranes were cut for immunoblotting of more than one protein.

Fig 2G

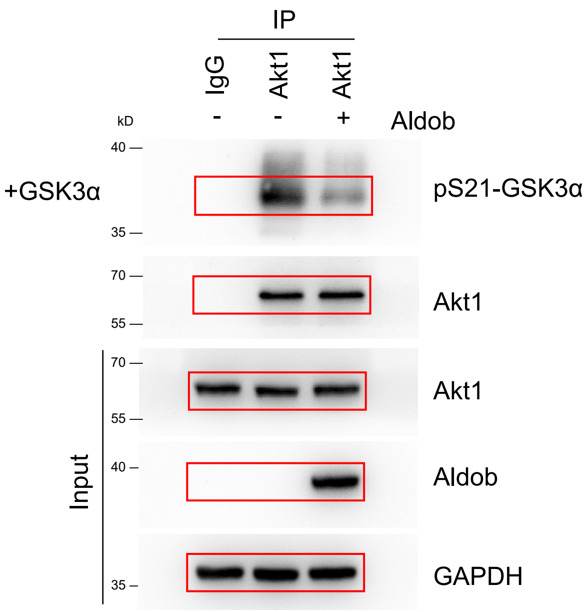

Fig 3D

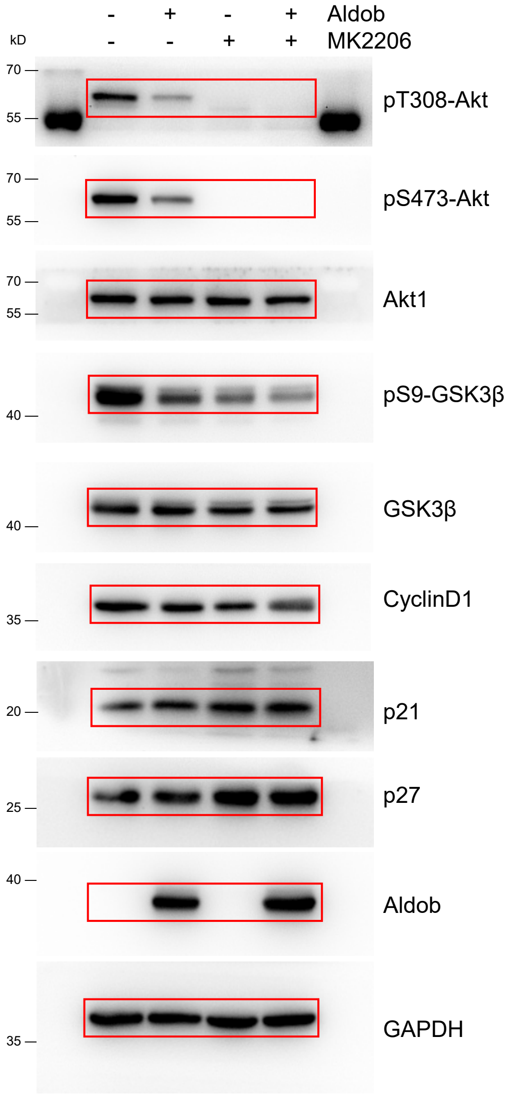

1. Images were acquired by chemiluminescence CCD camera-based digital imaging instruments (Tanon 5200 Chemiluminescent Imaging System).
2. Approx. molecular weight ladder was indicated.
3. PVDF Membranes were cut for immunoblotting of more than one protein.

Fig 4A

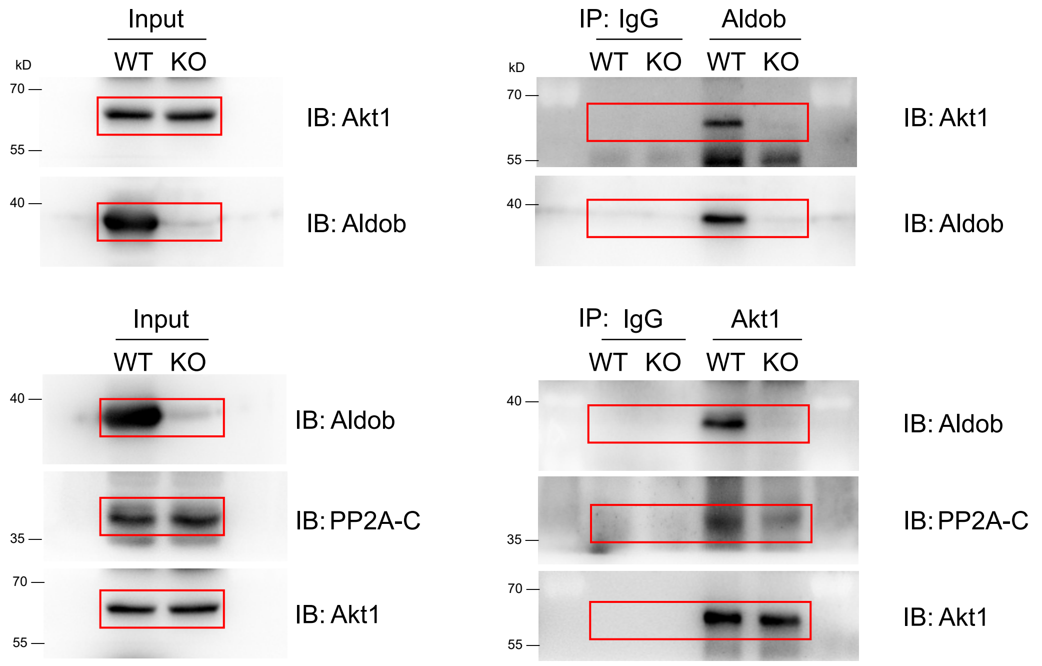

Fig 4B

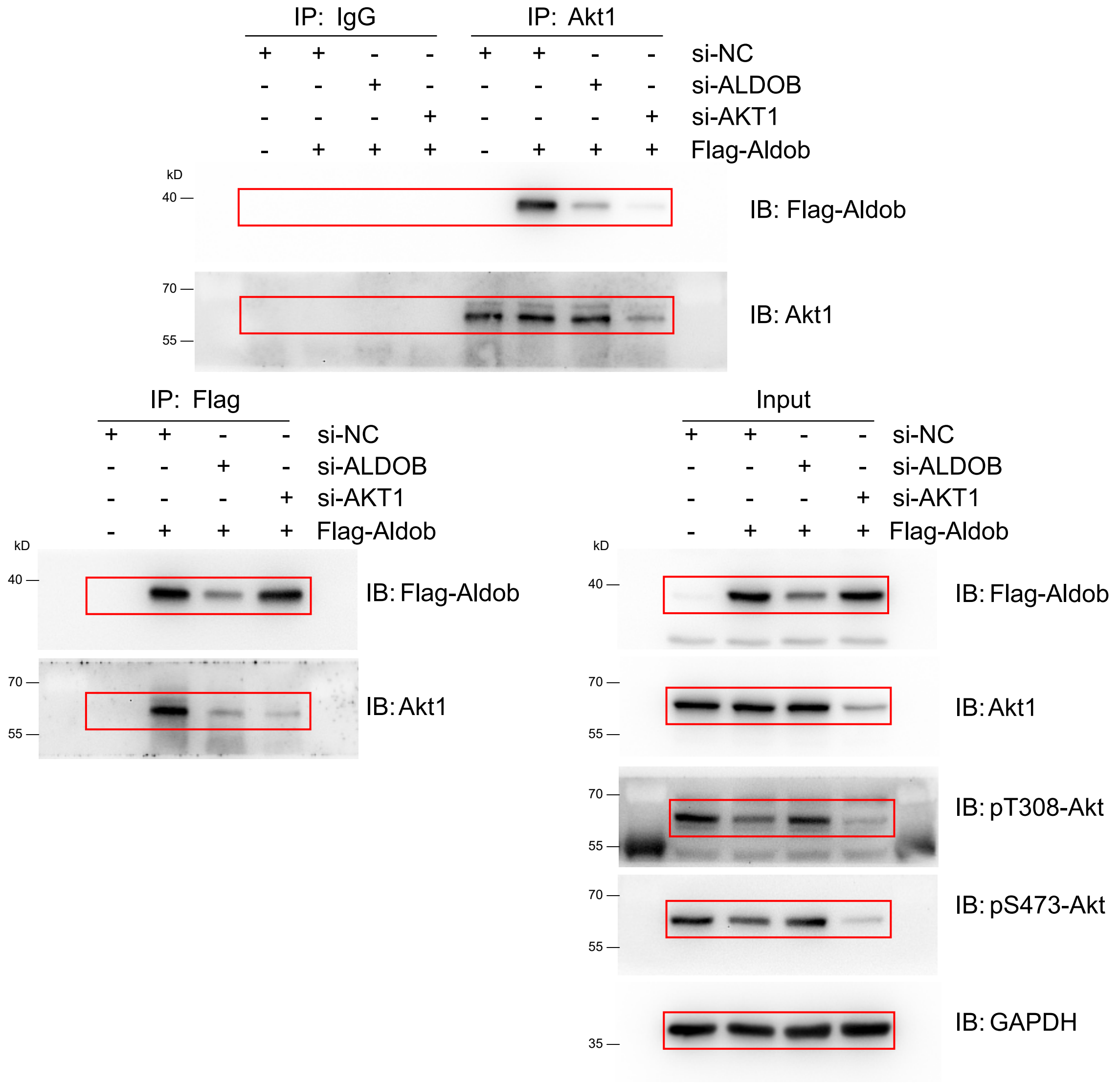

1. Images were acquired by chemiluminescence CCD camera-based digital imaging instruments (Tanon 5200 Chemiluminescent Imaging System).
2. Approx. molecular weight ladder was indicated.
3. PVDF Membranes were cut for immunoblotting of more than one protein.

Fig 4C

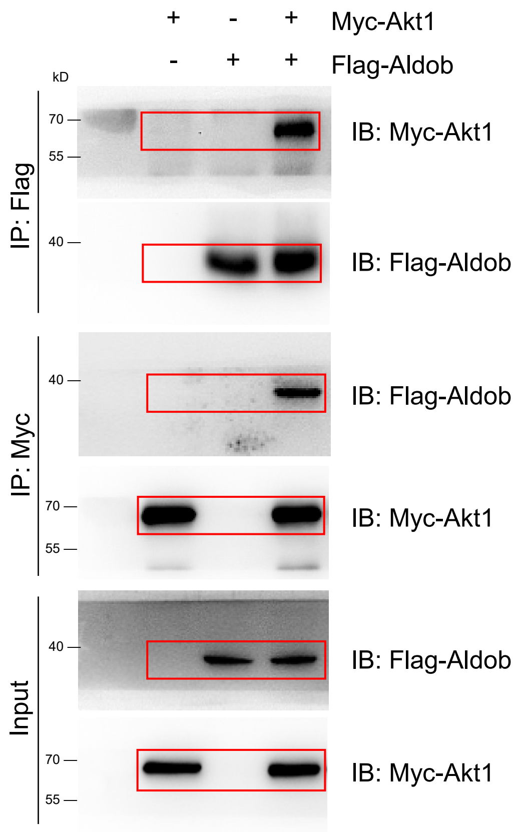

Fig 4D

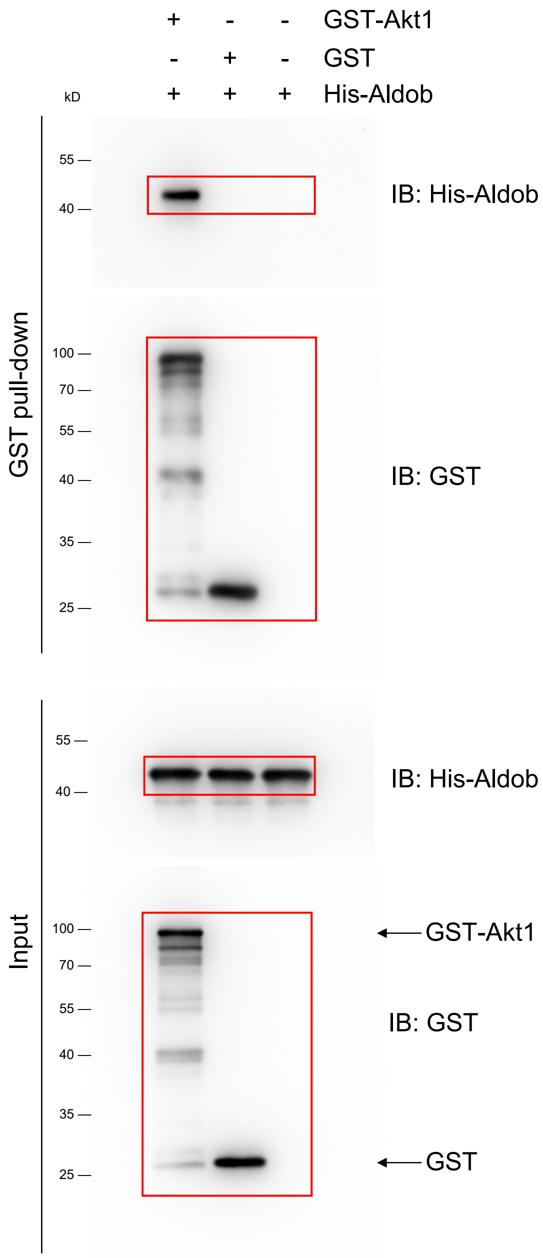

Fig 4E

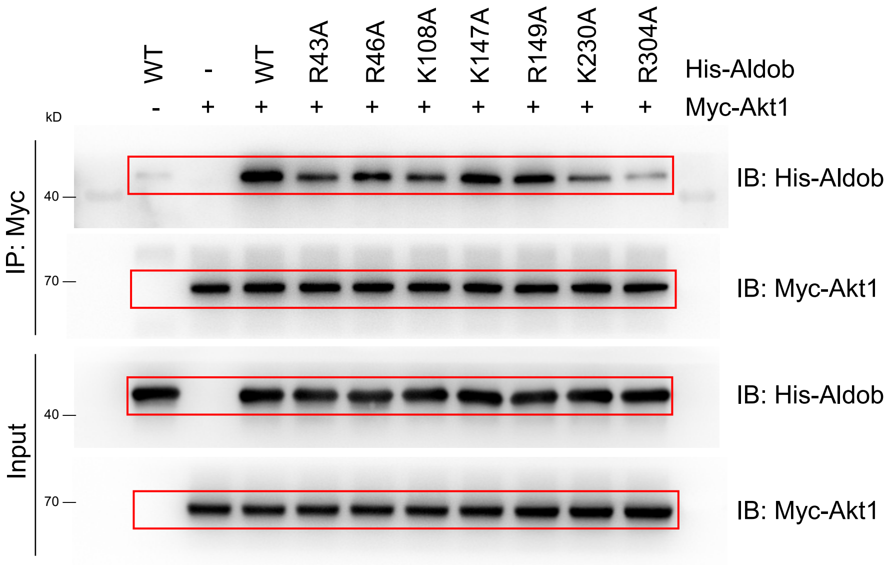

1. Images were acquired by chemiluminescence CCD camera-based digital imaging instruments (Tanon 5200 Chemiluminescent Imaging System).
2. Approx. molecular weight ladder was indicated.
3. PVDF Membranes were cut for immunoblotting of more than one protein.

Fig 4F

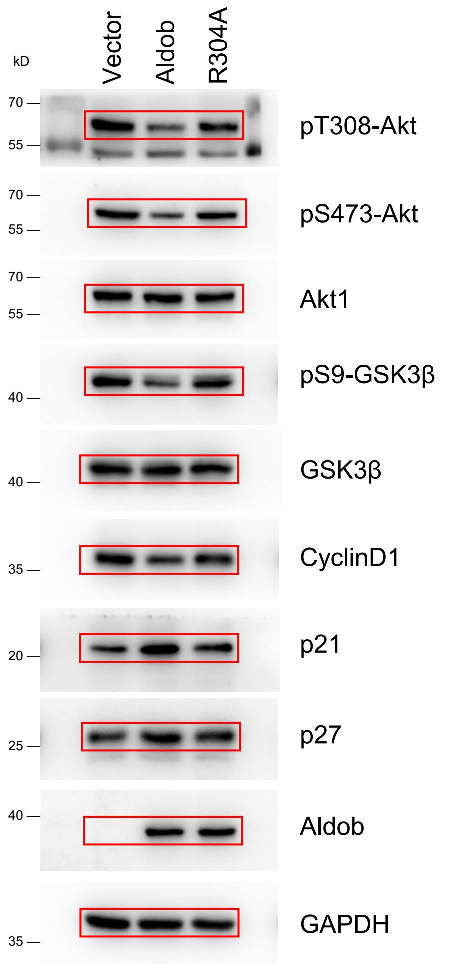

Fig 5A

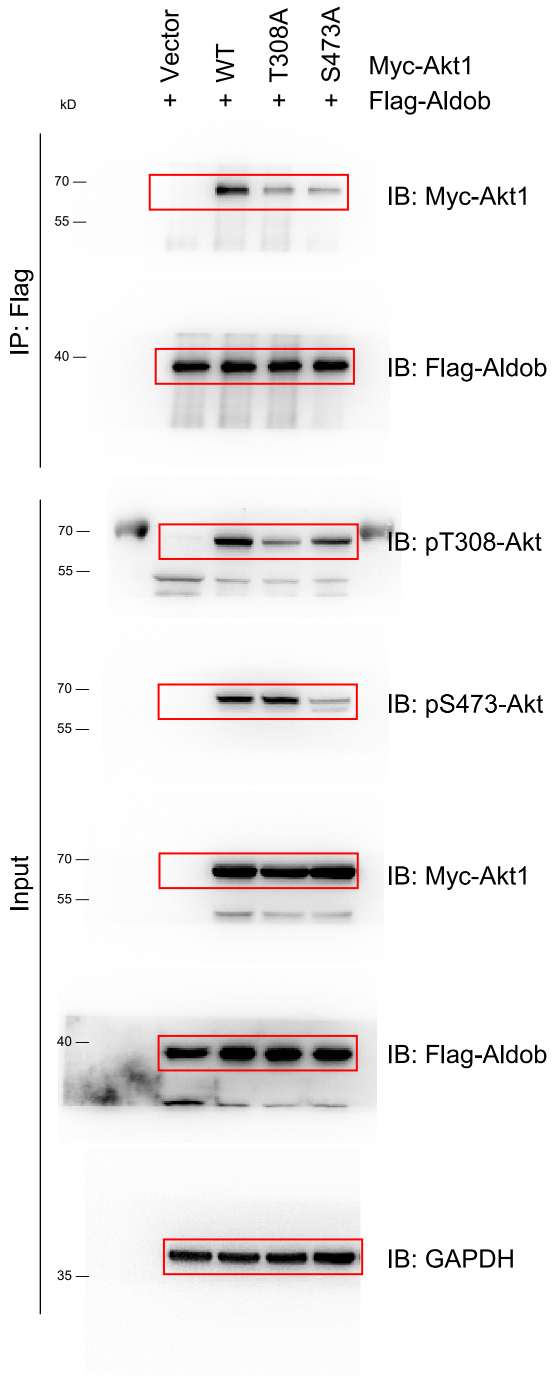

1. Images were acquired by chemiluminescence CCD camera-based digital imaging instruments (Tanon 5200 Chemiluminescent Imaging System).
2. Approx. molecular weight ladder was indicated.
3. PVDF Membranes were cut for immunoblotting of more than one protein.

Fig 5B

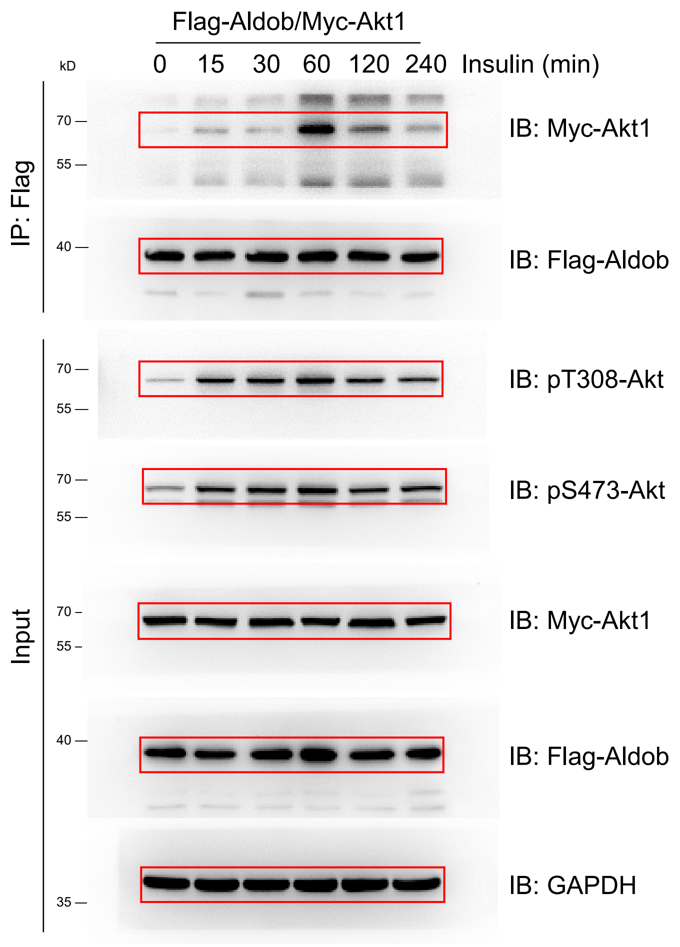

Fig 5C

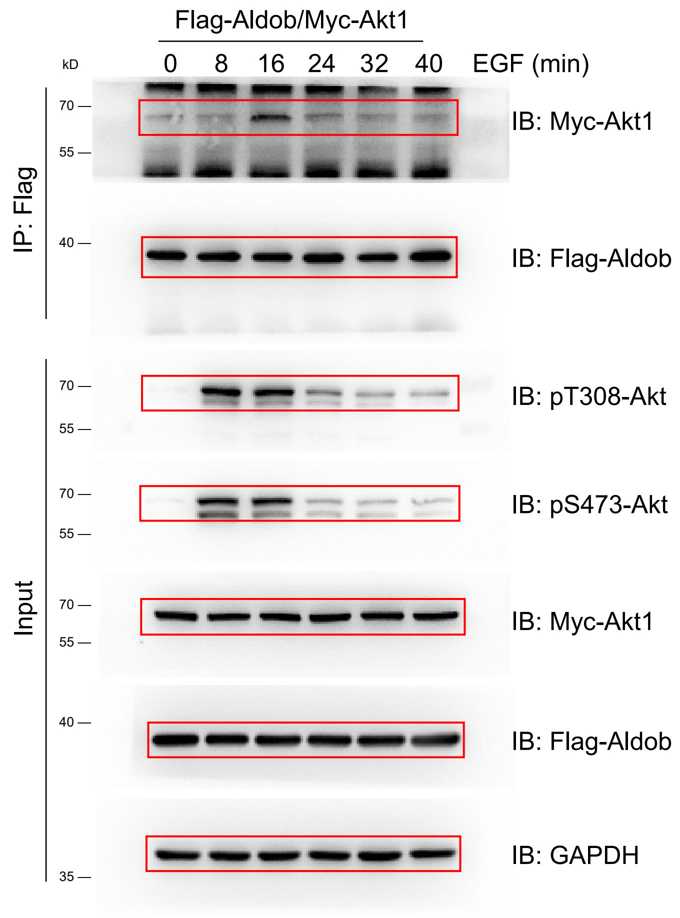

1. Images were acquired by chemiluminescence CCD camera-based digital imaging instruments (Tanon 5200 Chemiluminescent Imaging System).
2. Approx. molecular weight ladder was indicated.
3. PVDF Membranes were cut for immunoblotting of more than one protein.

Fig 5D

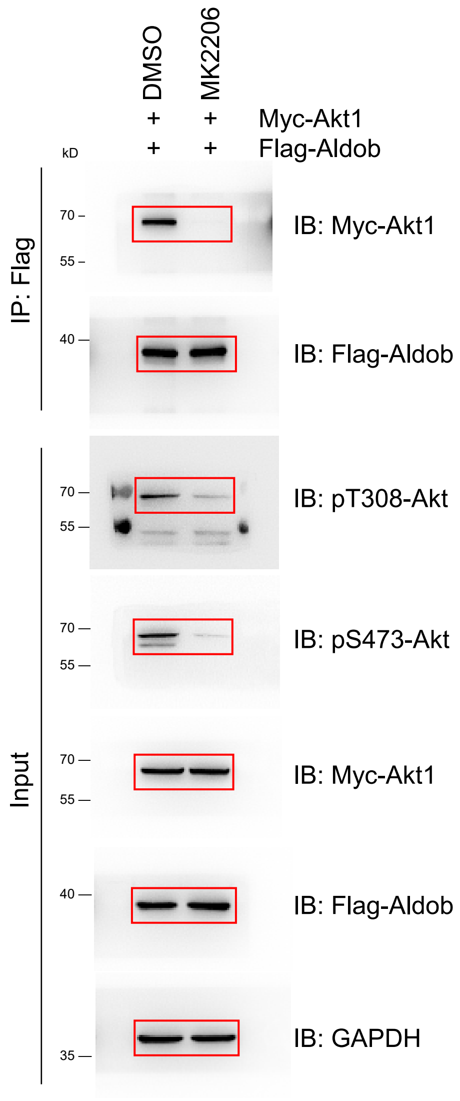

Fig 6A

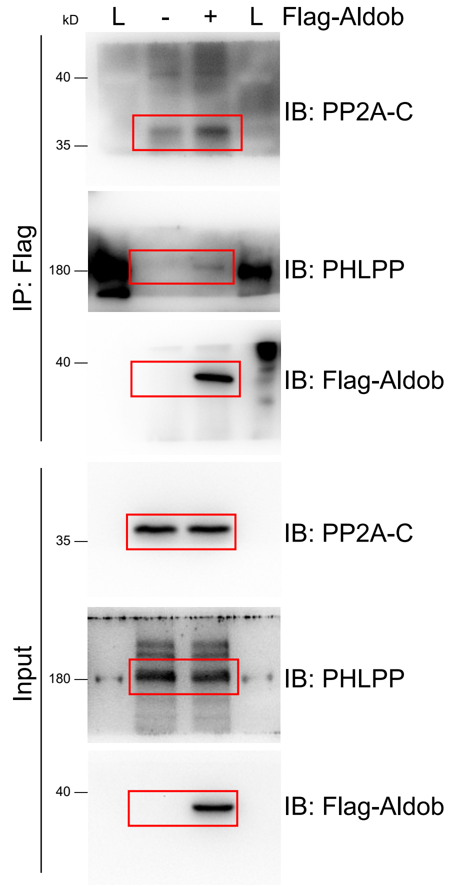

Fig 6B

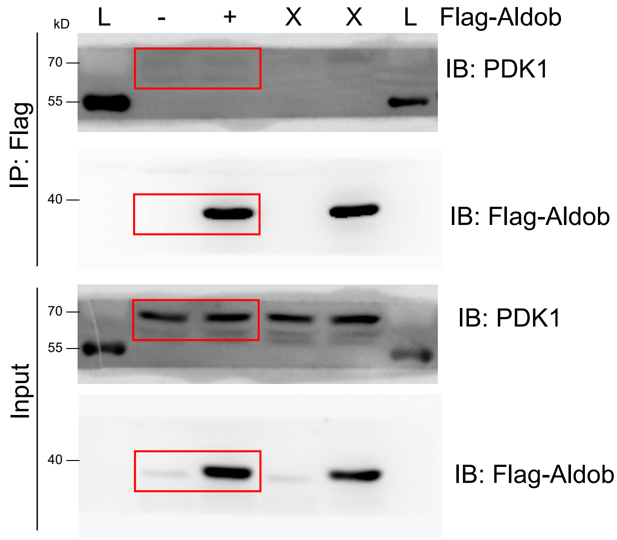

1. Images were acquired by chemiluminescence CCD camera-based digital imaging instruments (Tanon 5200 Chemiluminescent Imaging System).
2. Approx. molecular weight ladder (L) was indicated.
3. PVDF Membranes were cut for immunoblotting of more than one protein.

Fig 6C

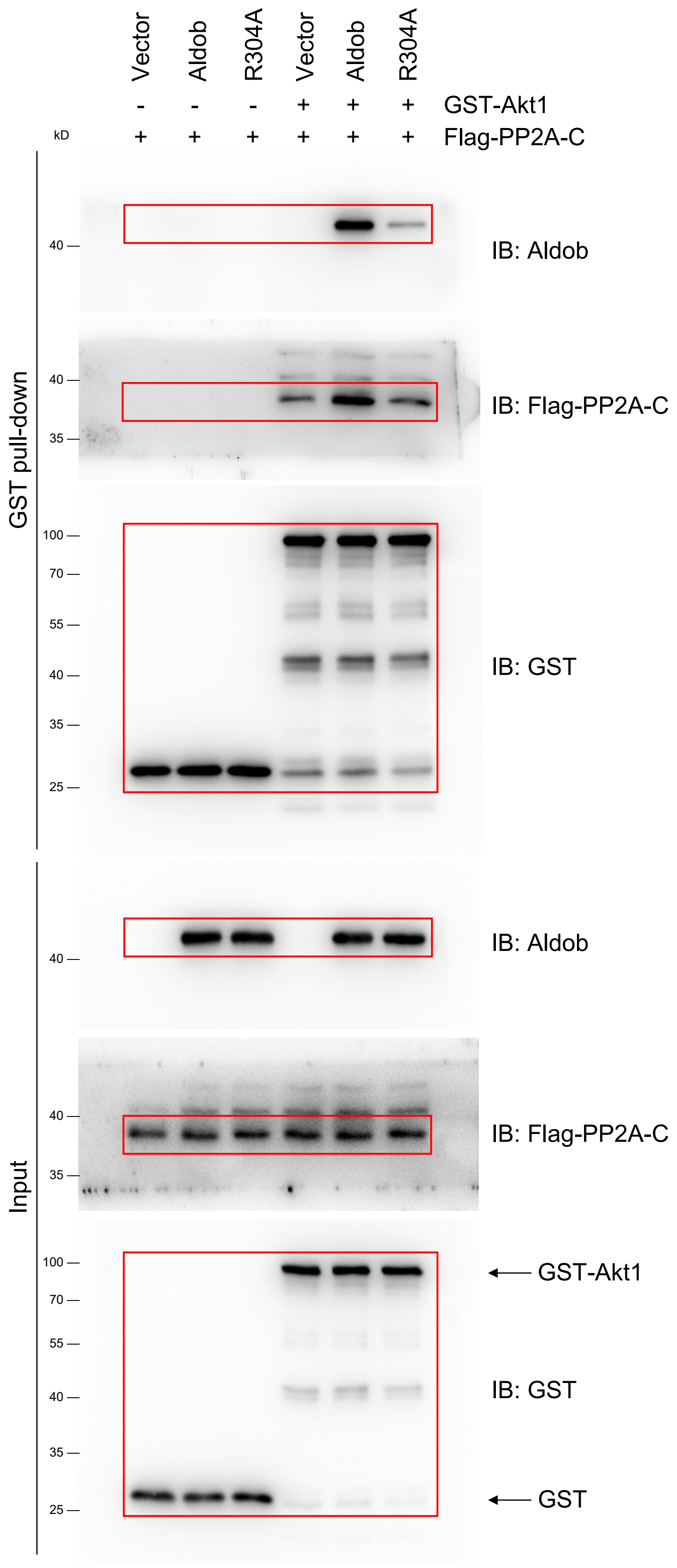

1. Images were acquired by chemiluminescence CCD camera-based digital imaging instruments (Tanon 5200 Chemiluminescent Imaging System).
2. Approx. molecular weight ladder was indicated.
3. PVDF Membranes were cut for immunoblotting of more than one protein.

Fig 6D

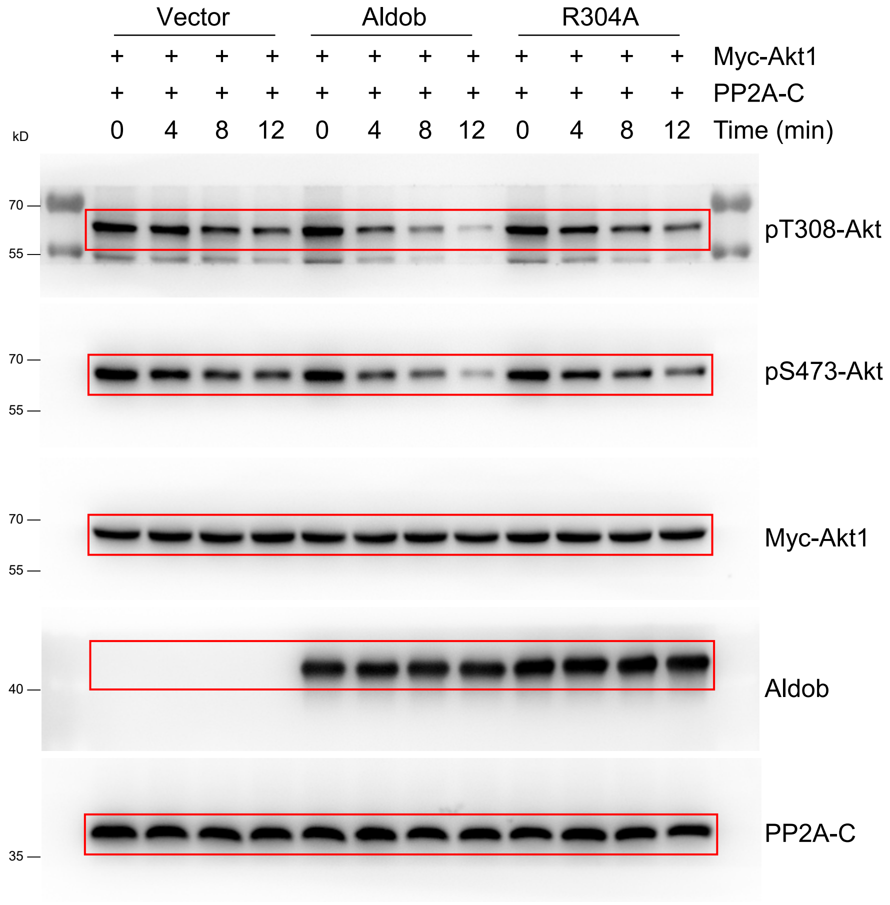

Fig 6E

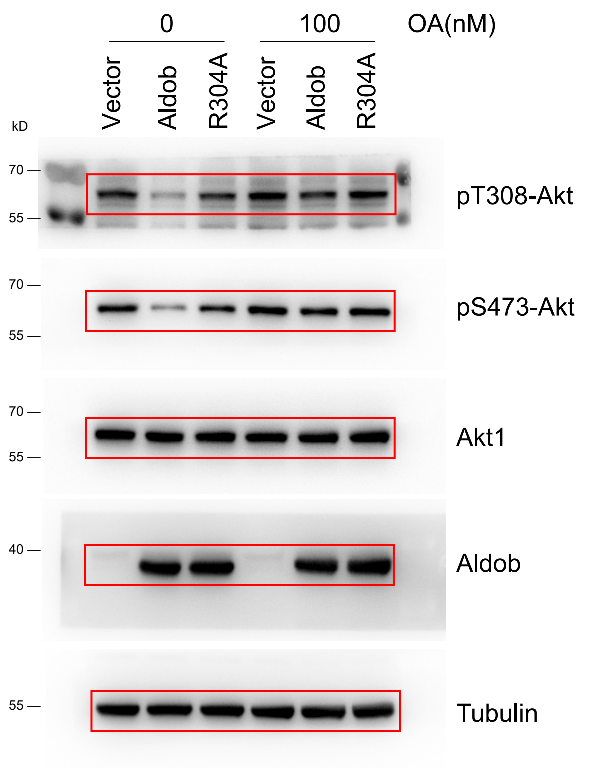

1. Images were acquired by chemiluminescence CCD camera-based digital imaging instruments (Tanon 5200 Chemiluminescent Imaging System).
2. Approx. molecular weight ladder was indicated.
3. PVDF Membranes were cut for immunoblotting of more than one protein.

Fig 6F

Left panel

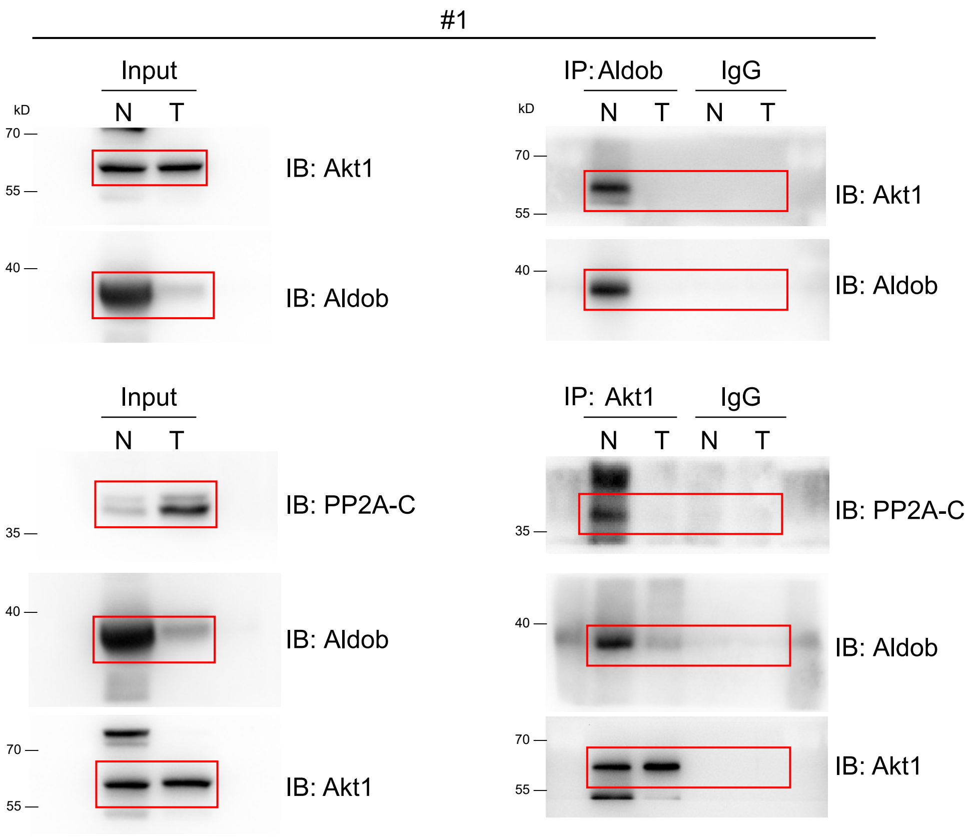

Right panel

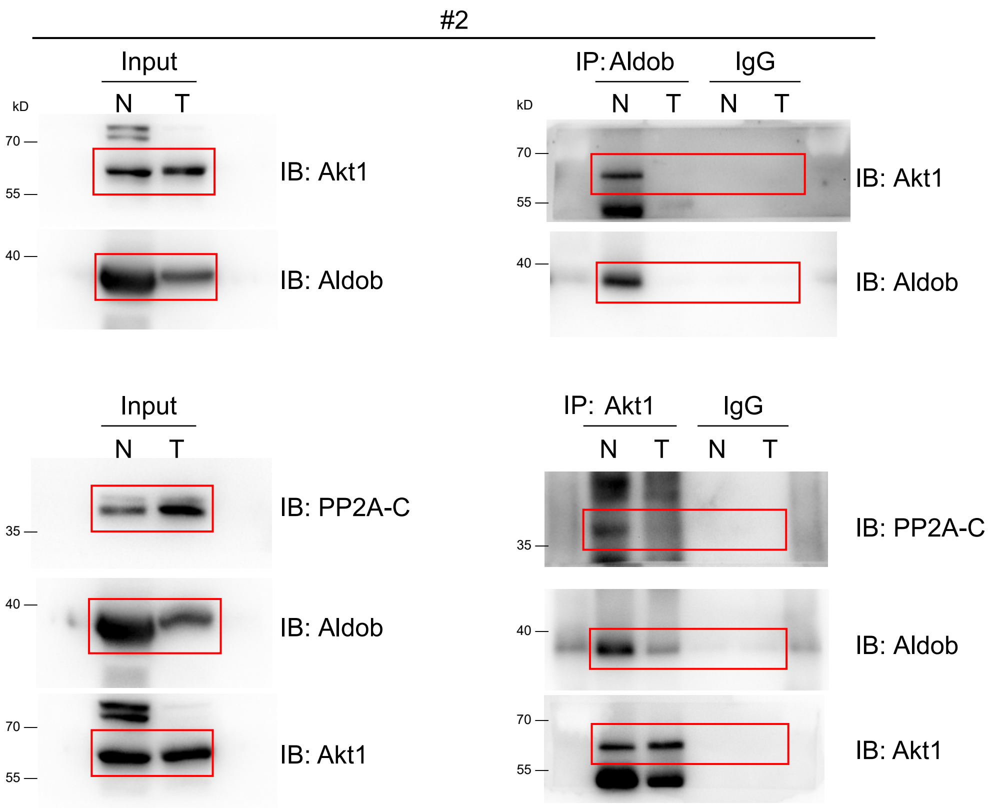

1. Images were acquired by chemiluminescence CCD camera-based digital imaging instruments (Tanon 5200 Chemiluminescent Imaging System).
2. Approx. molecular weight ladder was indicated.
3. PVDF Membranes were cut for immunoblotting of more than one protein.

Fig 7D

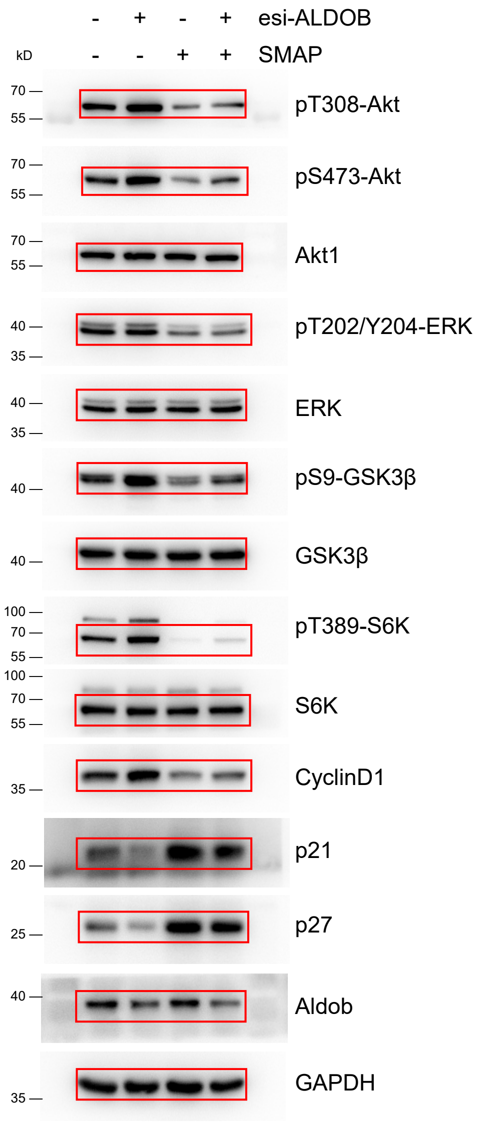

Fig 7H

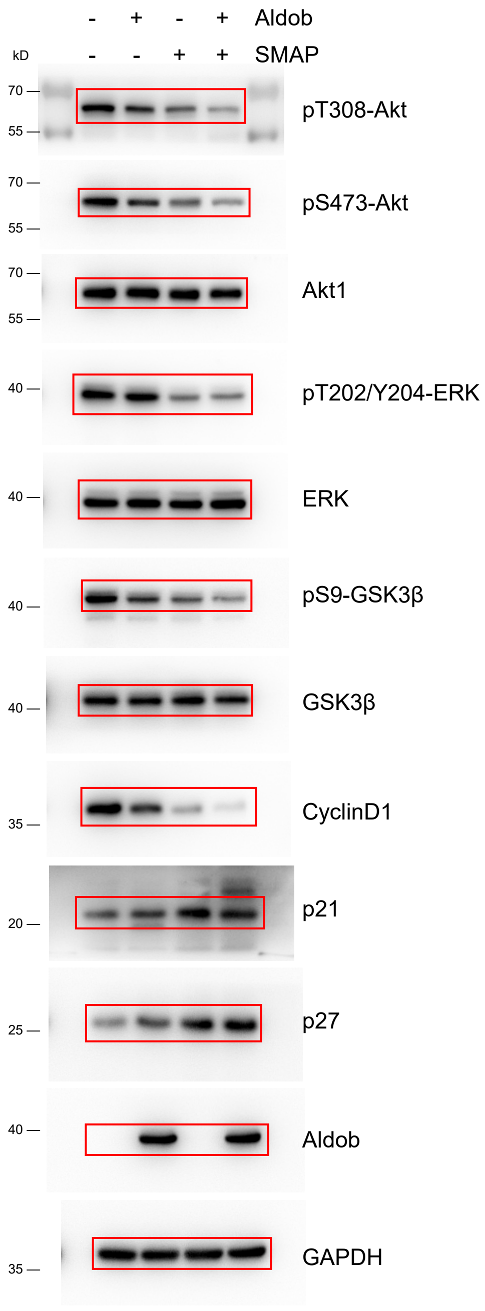

1. Images were acquired by chemiluminescence CCD camera-based digital imaging instruments (Tanon 5200 Chemiluminescent Imaging System).
2. Approx. molecular weight ladder was indicated.
3. PVDF Membranes were cut for immunoblotting of more than one protein.

Raw Images of Western blot

Supplementary Figures

S1B Fig

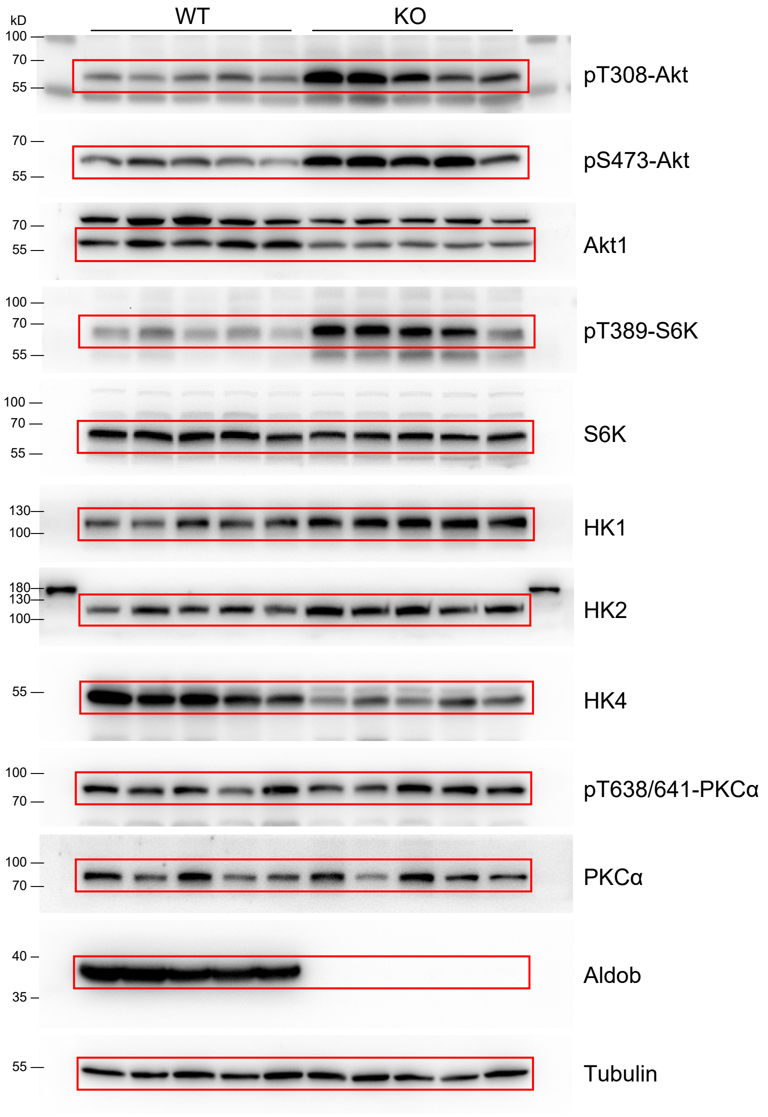

1. Images were acquired by chemiluminescence CCD camera-based digital imaging instruments (Tanon 5200 Chemiluminescent Imaging System).
2. Approx. molecular weight ladder was indicated.
3. PVDF Membranes were cut for immunoblotting of more than one protein.

S2D Fig

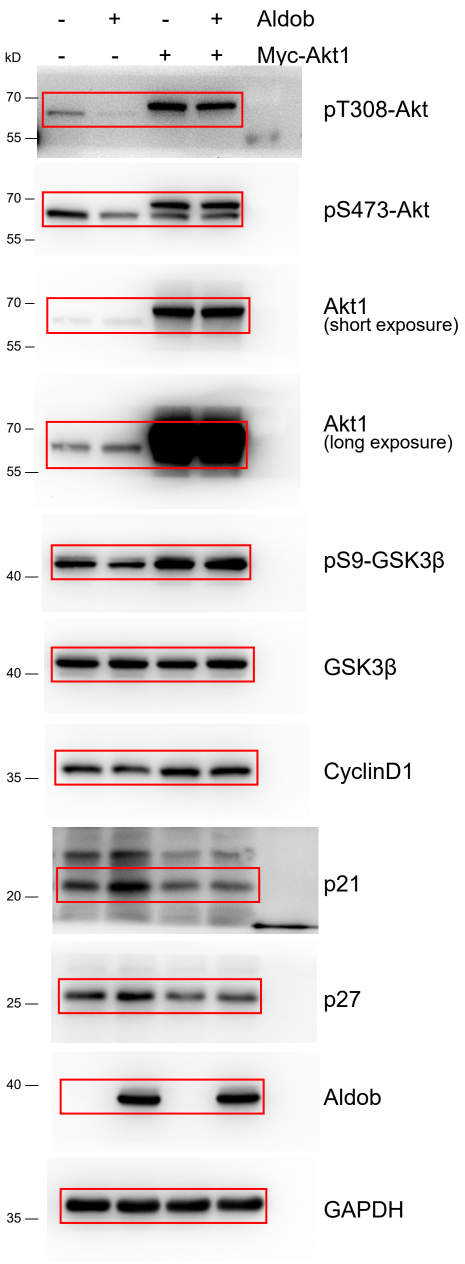

S2F Fig

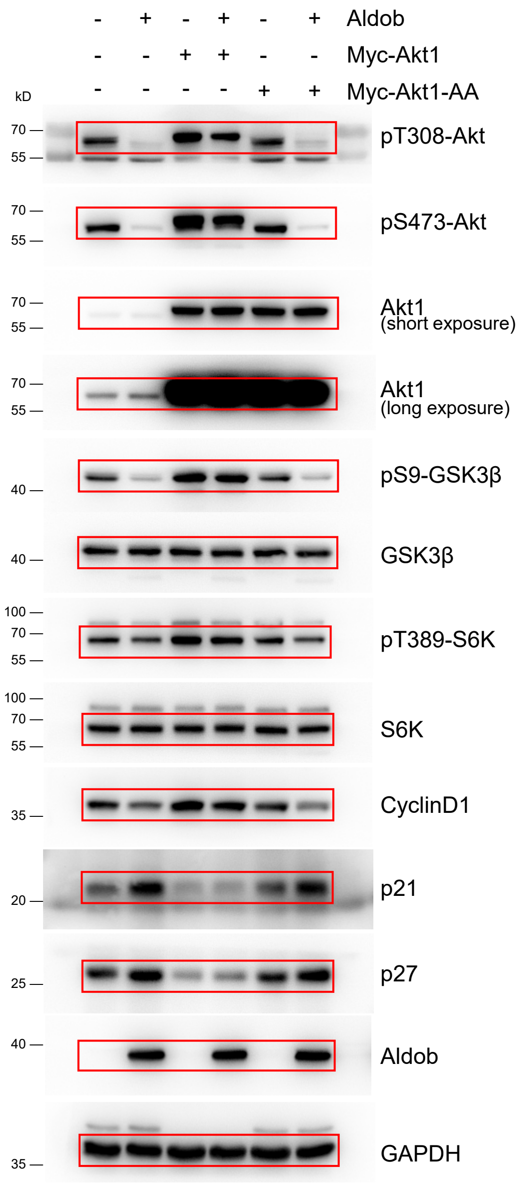

1. Images were acquired by chemiluminescence CCD camera-based digital imaging instruments (Tanon 5200 Chemiluminescent Imaging System).
2. Approx. molecular weight ladder was indicated.
3. PVDF Membranes were cut for immunoblotting of more than one protein.

S3D Fig

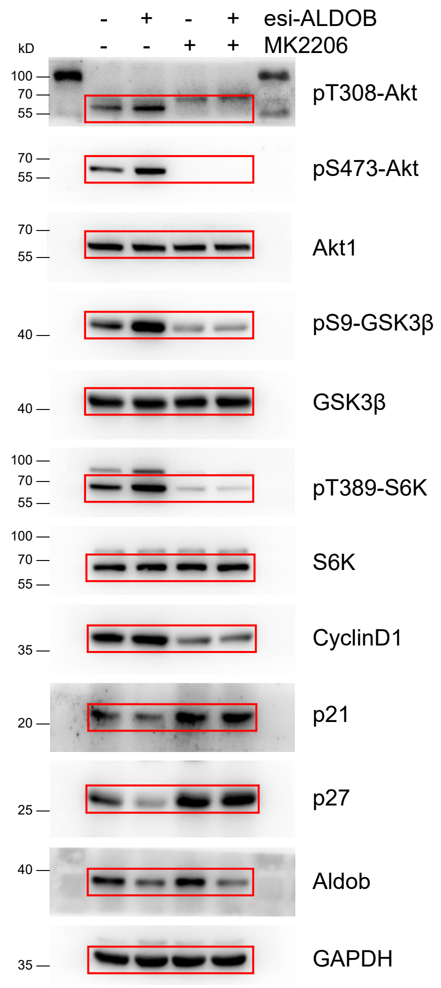

S4D Fig

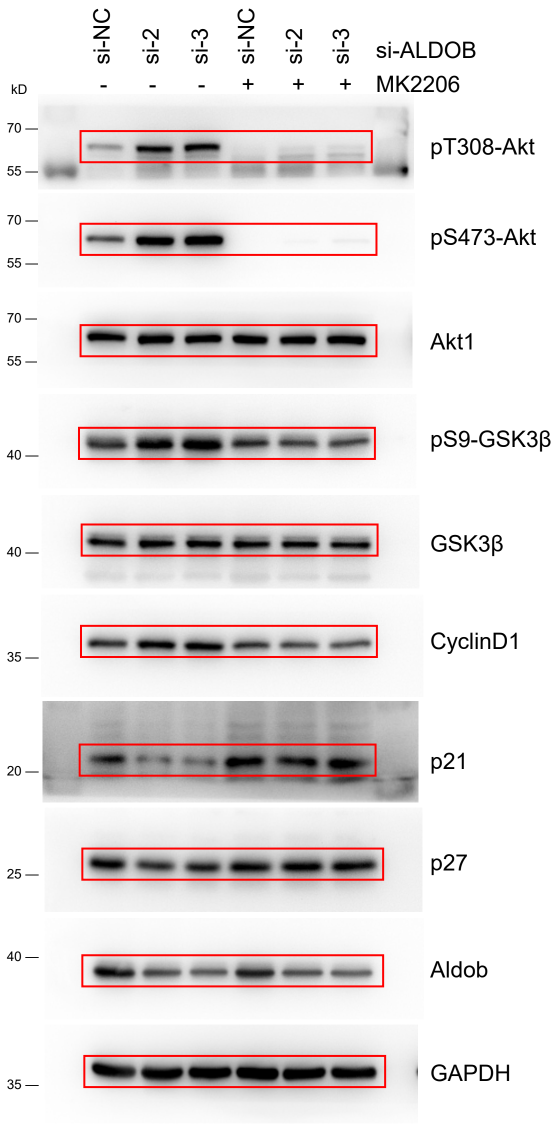

1. Images were acquired by chemiluminescence CCD camera-based digital imaging instruments (Tanon 5200 Chemiluminescent Imaging System).
2. Approx. molecular weight ladder was indicated.
3. PVDF Membranes were cut for immunoblotting of more than one protein.

S5D Fig

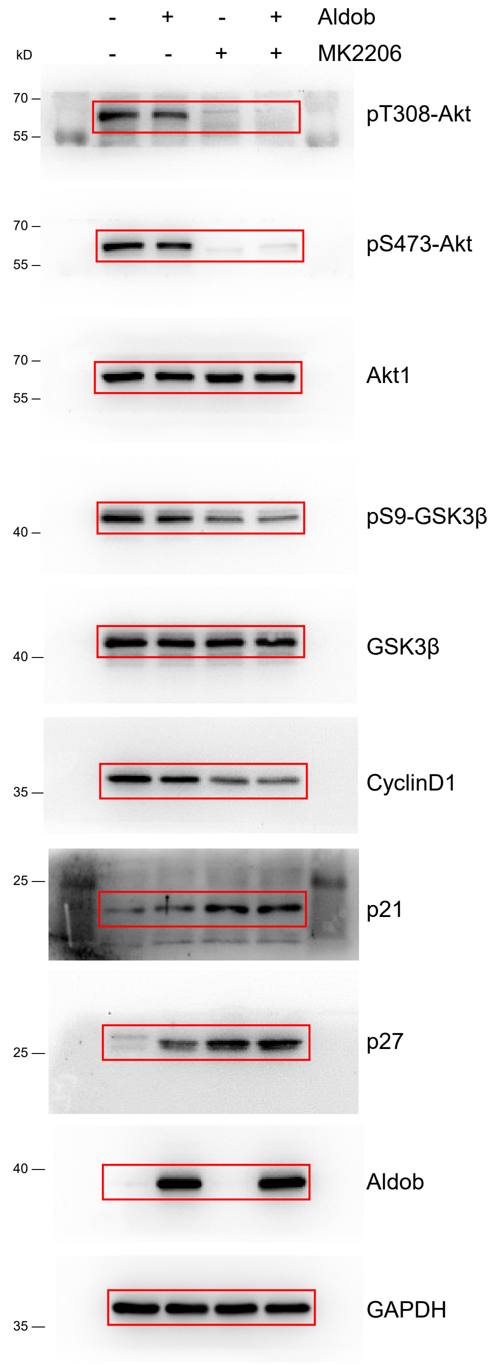

S5E Fig

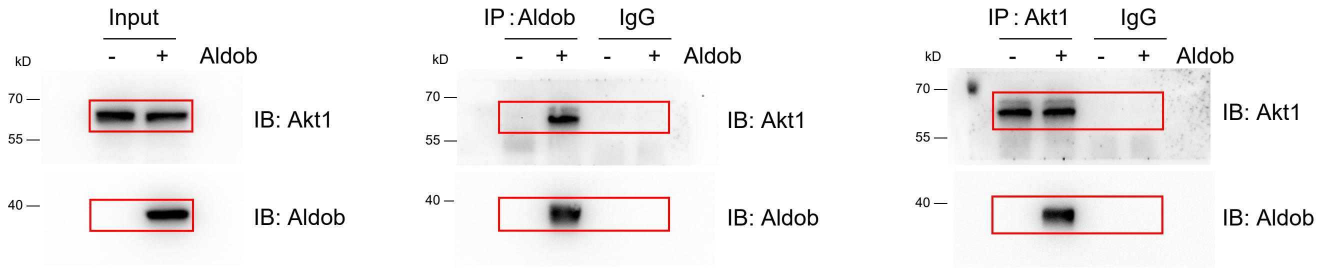

1. Images were acquired by chemiluminescence CCD camera-based digital imaging instruments (Tanon 5200 Chemiluminescent Imaging System).
2. Approx. molecular weight ladder was indicated.
3. PVDF Membranes were cut for immunoblotting of more than one protein.

S6B Fig

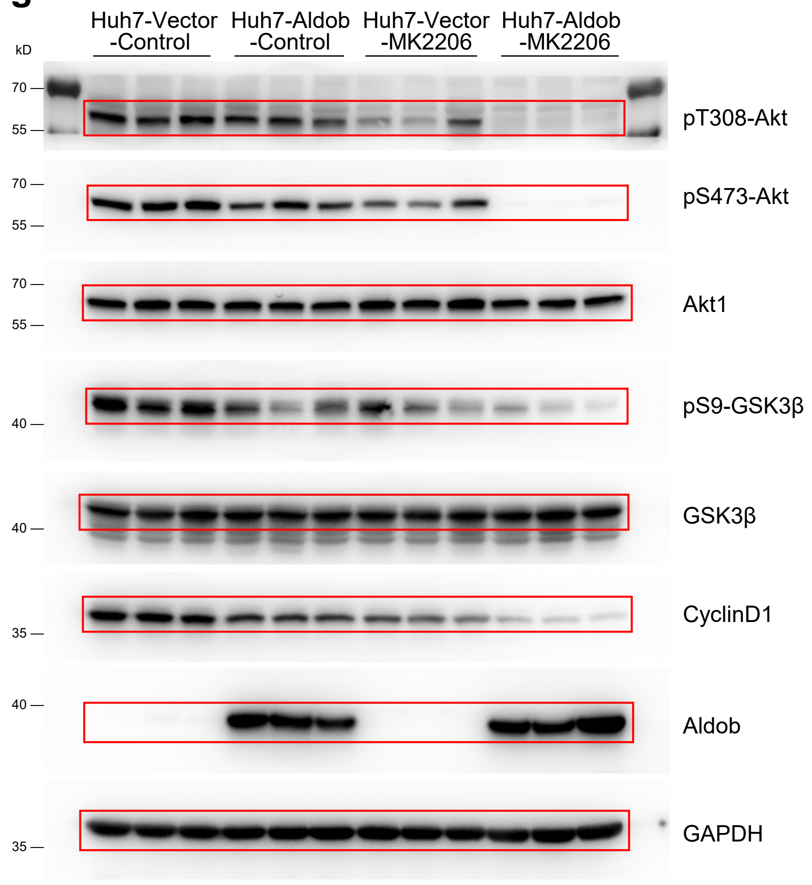

S7A Fig

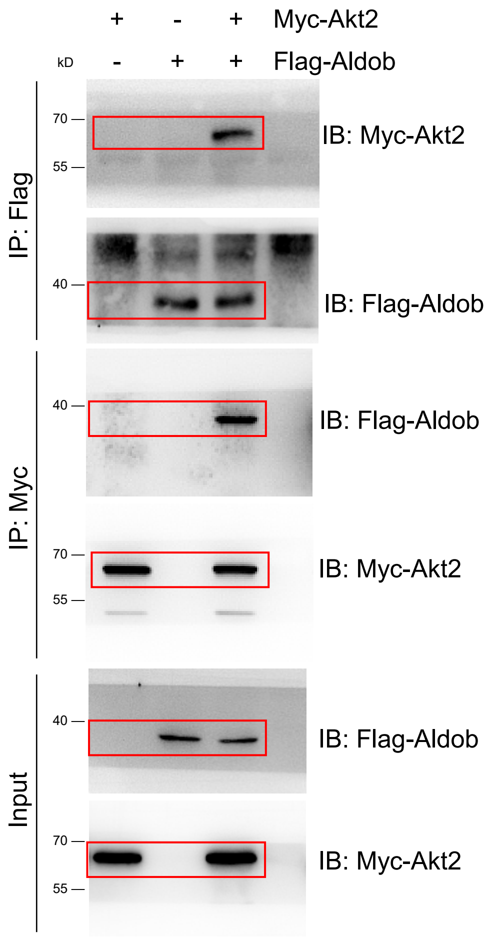

S7B Fig

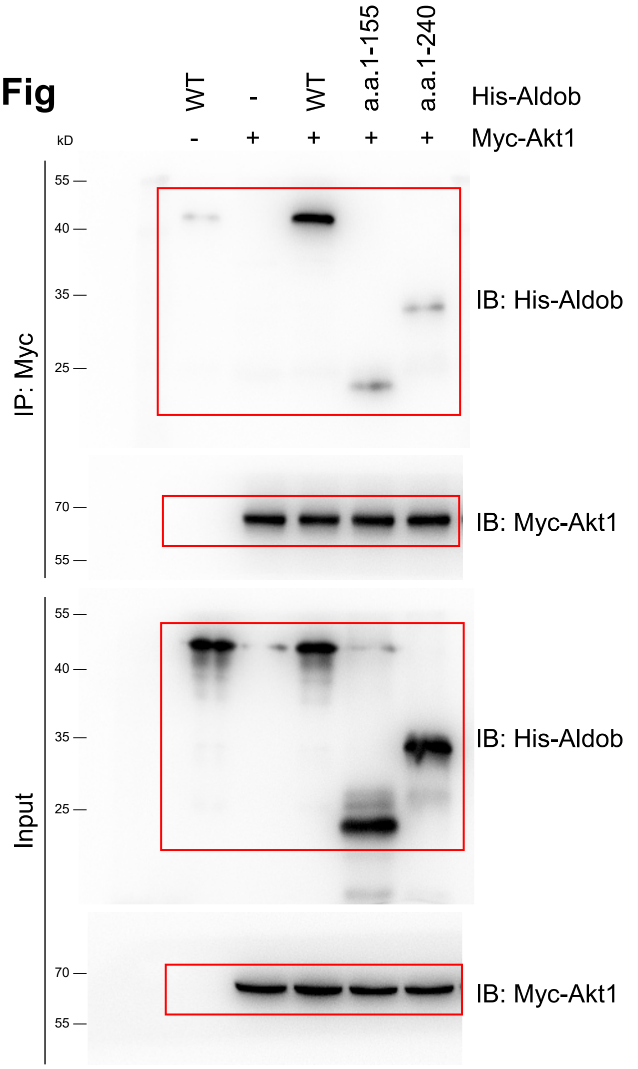

1. Images were acquired by chemiluminescence CCD camera-based digital imaging instruments (Tanon 5200 Chemiluminescent Imaging System).
2. Approx. molecular weight ladder was indicated.
3. PVDF Membranes were cut for immunoblotting of more than one protein.

S7C Fig

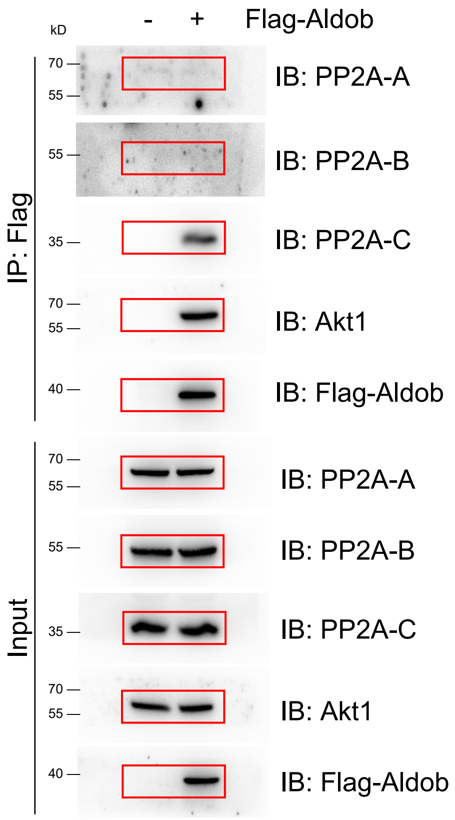

S7D Fig

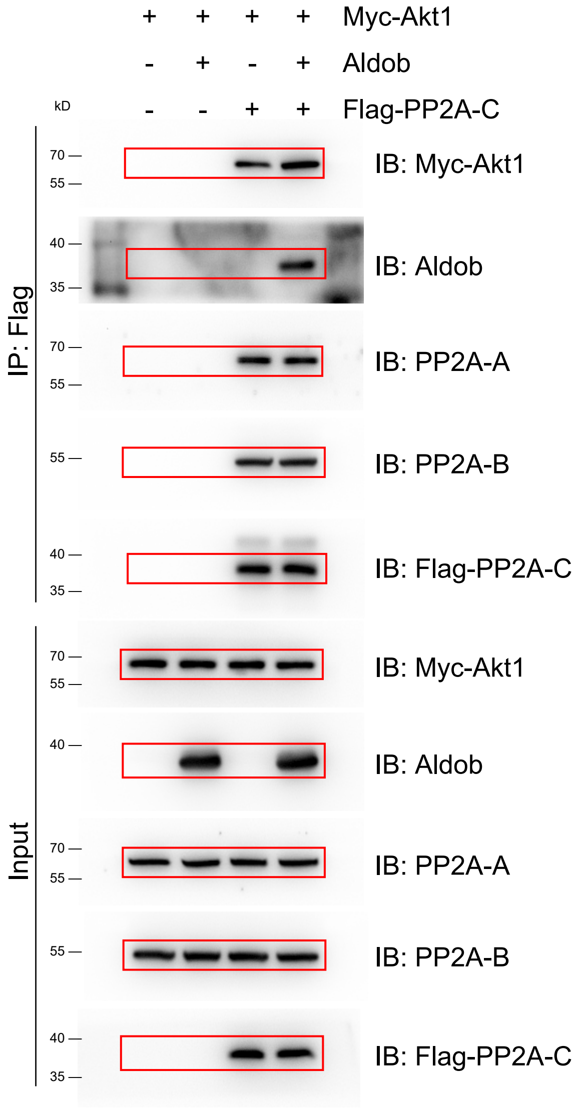

1. Images were acquired by chemiluminescence CCD camera-based digital imaging instruments (Tanon 5200 Chemiluminescent Imaging System).
2. Approx. molecular weight ladder was indicated.
3. PVDF Membranes were cut for immunoblotting of more than one protein.

S8A Fig

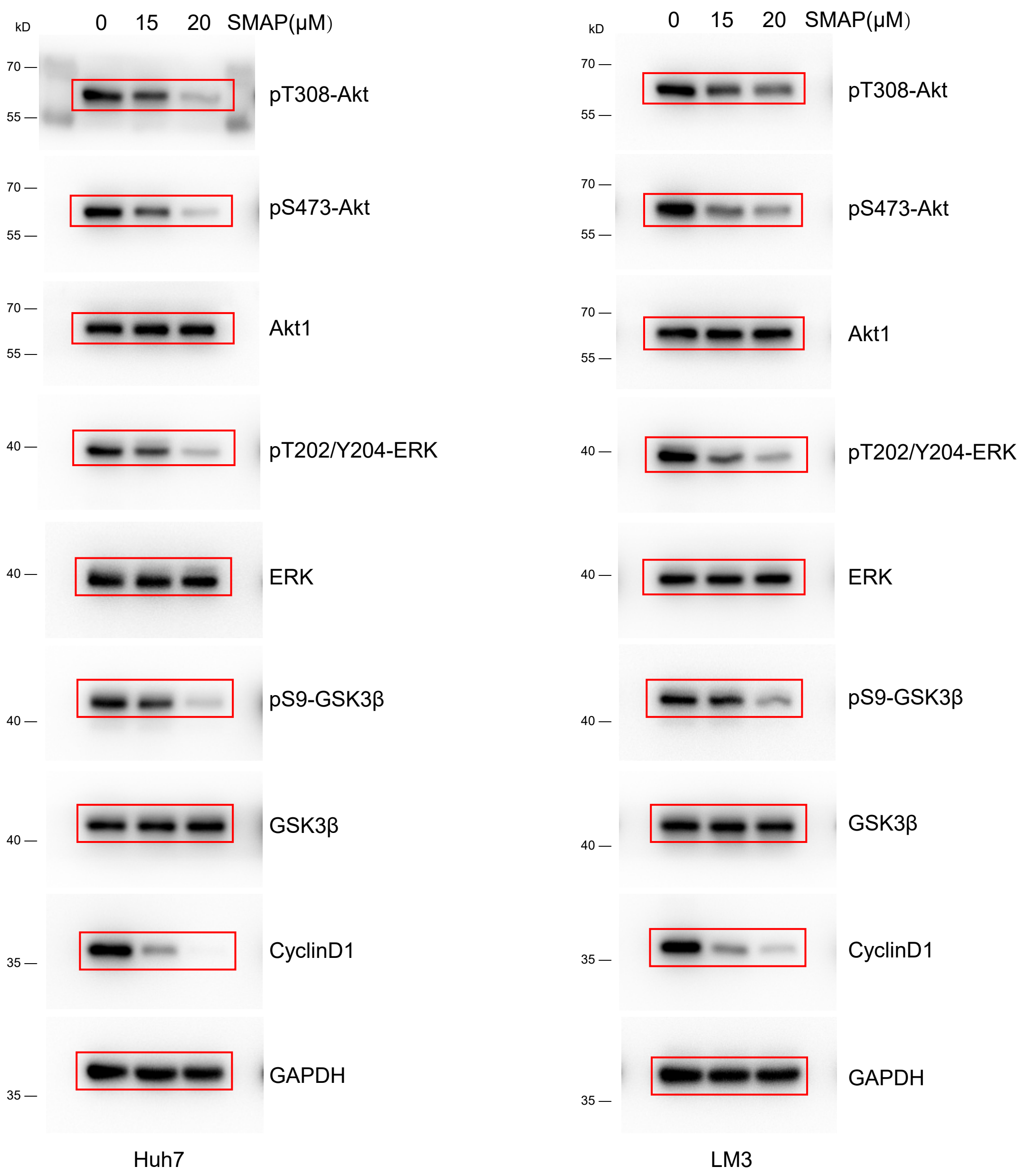

1. Images were acquired by chemiluminescence CCD camera-based digital imaging instruments (Tanon 5200 Chemiluminescent Imaging System).
2. Approx. molecular weight ladder was indicated.
3. PVDF Membranes were cut for immunoblotting of more than one protein.

S8D Fig

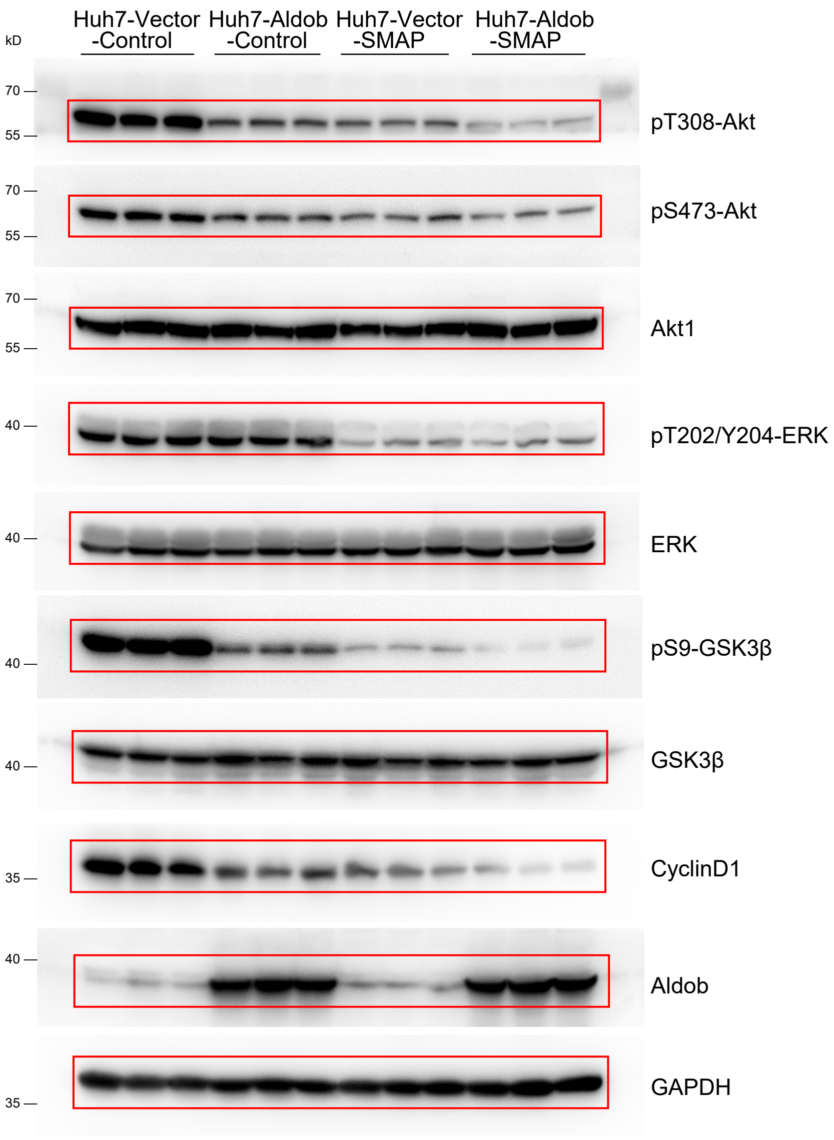

1. Images were acquired by chemiluminescence CCD camera-based digital imaging instruments (Tanon 5200 Chemiluminescent Imaging System).
2. Approx. molecular weight ladder was indicated.
3. PVDF Membranes were cut for immunoblotting of more than one protein.
