# Supplementary material for: Loss of hepatic aldolase B activates Akt and promotes hepatocellular carcinogenesis by destabilizing the Aldob/Akt/PP2A protein complex
Source: PLoS Biol. 2020 Dec 4;18(12):e3000803. doi: 10.1371/journal.pbio.3000803 (PMC7744066; doi:10.1371/journal.pbio.3000803)
Supplement: Attachment — Submitted filename: PBIOLOGY-D-20-01535_Response to Reviewers.pdf [file pbio.3000803.s013.pdf]

Reviewer #1:

The data presented clearly show an inverse correlation between Aldob expression and Akt activity. However, as discussed below, there are issues as to how these findings can be consistent with known role of Akt in cancer and glucose metabolism and some question as to how much of what is currently presented is novel. These concerns will have to be addressed before it can be accepted for publication.

Response: We thank the reviewer for the overall enthusiasm about our manuscript and will address the concerns point-to-point below.

Major Points

1. The authors must probe for expression for Aldoa expression. Aldoa is ectopically expressed in many cancers. Although it is the muscle-specific isoform, in fact it becomes the most expressed aldolase isoforms in many cancers (Chang et al, 2018, Trends Endocrinol. Metab. 29:549-559). While the mechanism presented here is independent of Aldob enzymatic activity and Aldoa expression would not affect the main findings of the paper, nevertheless this would have implications on the metabolic aspect and suggest that it is not necessary to divert glucose into the PPP to bypass the aldolase step.

Response: We thank the reviewer for raising concerns regarding the roles of Aldoa in our mechanism. Aldolase family includes Aldoa, Aldob, and Aldoc [1]. Aldoa is expressed in embryos and is abundantly available in adult muscle tissue. Aldob is the predominant isoform in liver, kidney and small intestine. Aldoc is abundant in the central nervous system. We agree with the reviewer that Aldoa has attracted increasing research interest in cancer. However, the role of Aldoa in HCC remains to be clearly defined. Previous studies appear to show that Aldoa expression is compensatorily increased in hepatocellular carcinoma (HCC) at RNA or protein levels, presumably due to the loss of Aldob [2-5]. However, in our recent study [6], we found that Aldoa expression was slightly ***decreased***, whereas there was no significant change of Aldoc in HCC tumor tissues compared to normal liver tissues ***(Extended Data Fig. 1g–j)***. Furthermore, Aldoa and Aldoc expression did ***not correlate*** with HCC survival

(Extended Data Fig. 1k–m). More importantly, we detected total aldolase enzymatic activity in 4 pairs of human HCC samples and found that the overall aldolase enzymatic activity was significantly ***decreased*** in tumor and PVTT (Portal Vein Tumour Thrombus) tissues as compared to matched adjacent normal liver tissues ***(Extended Data Fig. 3d)***. Furthermore, the aldolase activities are comparable to those in the liver tissue of Aldob KO mice ***(Extended Data Fig. 3c)***. ***All these data strongly suggest that Aldob plays a more important role than Aldoa in HCC and Aldoa expression is not enough to compensate for the decrease of Aldob in HCC even if there is some Aldoa expression in HCC***. Therefore, we respectfully disagree with the reviewer "that it is not necessary to divert glucose into the PPP to bypass the aldolase step".

In fact, our recent study clearly demonstrated that loss of Aldob leads to upregulation of G6PD activity and PPP to meet the metabolic demands for HCC tumor cells ***(Figure 6j)*** [6]. In normal cells, Aldob expression helps to maintain glycolysis, gluconeogenesis, and TCA by preventing over-acting PPP via stabilizing Aldob/G6PD/p53. However, loss of Aldob in tumor cells liberates the inhibition on G6PD and PPP, leading to an enhanced PPP flux to meet the bioenergetics and biosynthetic demands during HCC progression. This novel metabolic re-routing enables cancer cells to overcome the seemingly paradoxical loss of Aldob by upregulating PPP to provide NADPH and GSH for detoxification of intracellular reactive oxygen species (ROS) and building blocks for DNA biosynthesis. The increased PPP intermediates can be converted into F6P and GAP: F6P comes back to oxidative PPP, while GAP enters downstream of glycolysis and continues to be converted into lactate or enter the TCA cycle for ATP production. This metabolic feature is the most economical and fastest way to produce energy, biosynthetic building blocks and reducing equivalents for cancer cells to quickly respond and meet the bio-energetic demands for rapid proliferation.

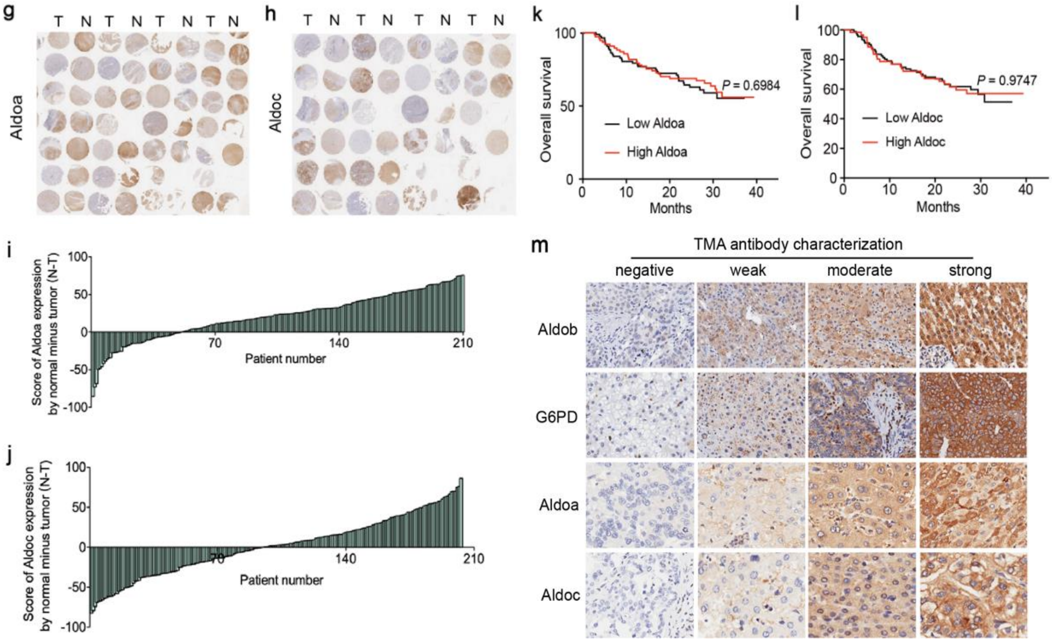

Extended Data Fig. 1. **g-h**, Representative TMA images with Aldoa (**g**) and Aldoc (**h**) antibody. i-j, Quantification of protein expression score of the matched normal tissues minus tumors from 184 HCC tissue microarray data with Aldoa (**i**) and Aldoc (**j**) antibody. Protein expression intensity is classified into negative (0-20 score), weak (21-100 score), moderate (101-180 score), strong (181-255 score). The calculation formula: (weak + moderate + strong)/(weak + moderate + strong + negative) x 100%. k-l, Kaplan-Meier survival curves in HCC patients with Aldoa (**k**, n = 214) and Aldoc (**l**, n = 203) high and low expression (patients are divided into two groups with high (>50) and low (< 50%) Aldoa or Aldoc expression). **m**, TMA antibody characterization of Aldob, G6PD, Aldoa, Aldoc (magnification: 200×). [6]

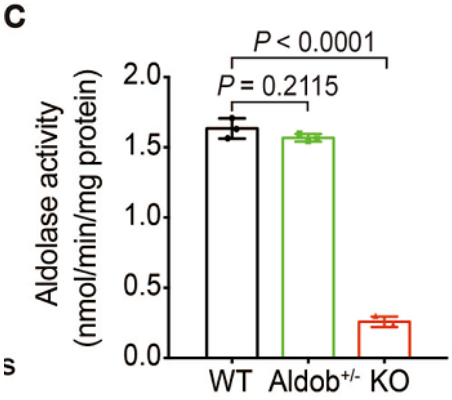

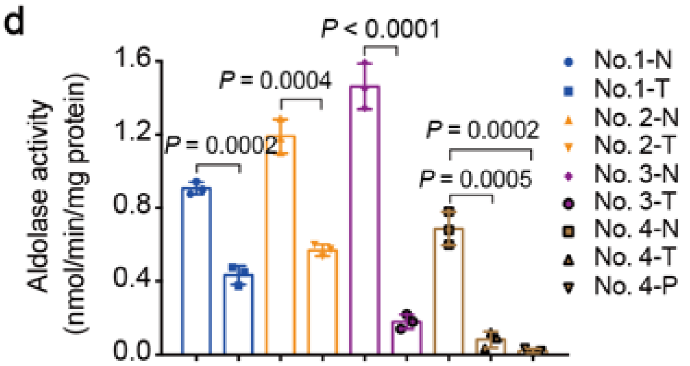

Extend Data Fig. 3. **c**, Aldolase enzymatic activity in WT and Aldob KO liver tissues (n = 3 biologically independent samples). **d**, Aldolase enzymatic activity in human HCC tissues (n = 3 biologically independent samples). [6]

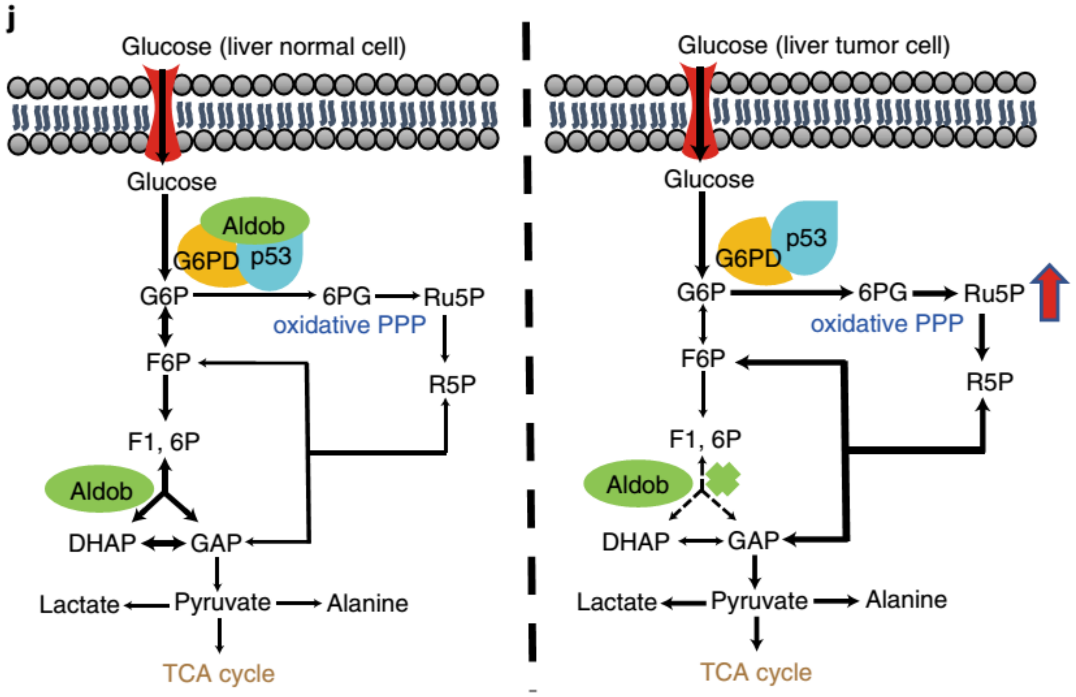

Figure 6. j, Summary scheme. Sufficient Aldob expression in normal liver cells maintains a metabolic homeostasis and prevents over-acting G6PD activity by interacting with G6PD and p53 to form a stable protein complex (Aldob/G6PD/p53) (left). Loss of Aldob results in the dissociation of Aldob–G6PD–p53 complex, which releases G6PD and enhances PPP metabolism to meet the bioenergetic demands for HCC progression (right). [6]

2. The authors make frequent references to a recent publication of theirs and that the current work is an extension of it. However this work is not cited in the references and I could not find it on Pubmed. Without any knowledge of its contents it is not possible to fairly assess the current manuscript as to whether it agrees with the earlier findings, or how much of the work being presented is novel.

Response: We are sorry for the inconvenience because our manuscript was under revisions in *Nature Cancer* at the initial submission of this manuscript and we included it as a supporting material. We have added the reference by Li et al [6] as ***ref. 18*** in the revision. The major finding of our previous work is summarized in the ***Figure 6j*** as shown above. In this study, we observed a novel non-enzymatic tumor-suppressive role of Aldob through a direct interaction with Akt in HCC. Aldob inhibits Akt activity and downstream signaling events that leads to the impairment of cell cycle progression and glucose uptake and metabolic flux to glycolysis and TCA cycle, thereby suppressing HCC cell proliferation and tumor growth. All these results are consistent with the conclusion in our recent publication that Aldob exhibits tumor suppressive function by acting as a metabolic switch in glucose metabolism in HCC *via* direct binding to G6PD and inhibiting pentose phosphate pathways [6]. Both studies support the tumor suppressive function of Aldob through regulating cell proliferation and glucose metabolism. On the basis of recent publication, our current work provides the mechanistic insights into how loss of Aldob results in paradoxical upregulation of glucose uptake and entire central carbon metabolism in HCC.

Our current work has multiple significant implications in understanding the underlying molecular mechanisms between Aldob and Akt during HCC progression. First of all, this study supports the tumor-suppressive role of Aldob and elucidates the potential contribution for Aldob deletion in the upstream signaling events leading to HCC progression and metabolic reprogramming. Abnormal activation of Akt signaling promotes tumor progression through upregulating cell cycle, metabolism and survival [7]. Activated Akt enhances glucose uptake and glycolysis by upregulation of the expression and activity of numerous glycolytic enzymes, such as hexokinases. Hexokinases catalyze the first irreversible step in glucose metabolism by phosphorylating glucose to glucose-6-phosphate, and plays a critical role in cellular glucose uptake and utilization [8]. Our data indicate that Aldob inhibited cell cycle progression, glucose uptake and metabolic flux to glycolysis and TCA cycle through downregulation of Akt signaling. Secondly, the data presented here indicate that Akt

kinase activity is downregulated through the novel protein interactions mediated by the glycolytic enzyme Aldob, independent of its enzymatic activity. Our current study has identified a close functional relationship among Aldob, Akt and PP2A, providing new mechanistic insights into the crucial upstream regulators involved in the Akt phosphorylation homeostasis. Aldob potentiates PP2A interaction and dephosphorylation of p-Akt, resulting in inhibition of Akt phosphorylation and kinase activity. Thirdly, we discovered that a specific small-molecule activator of PP2A (SMAP) efficiently attenuates the tumorigenic effects of Akt activation driven by Aldob deficiency in HCC, comparable to the antitumor efficacy of the well-studied allosteric Akt inhibitor MK2206. Our study suggests that inhibition of Akt activation resulted from the downregulation of Aldob is a potential therapeutic approach for HCC. PP2A activation using SMAP has the potential to be a new targeted therapeutic approach for HCC treatment.

3. Akt activation increases glucose uptake via glucose transporters and stimulation of hexokinase activity. The only way to resolve this with the loss of Aldob (assuming Aldoa is not expressed) is that glucose enters the PPP shunt and re-enters the glycolytic pathway as G3P. The authors should measure glucose uptake to see if it is increased or unchanged in their cell or mouse panel. HK1 levels are measured in mouse livers in Fig2A. However liver predominantly utilizes HK4, while HK2 is the isoform that is activated by Akt. Therefore the conclusion that a slight increase in HK1 levels is indicative of increased glucose metabolism in Aldob deficient cells is a very weak one. The authors need to show that glucose is taken up and mobilized to G6P in a way consistent with their model.

Response: We thank the reviewer for raising these concerns regarding the upregulation of glycolysis with the loss of Aldob and suggesting to provide more evidence of the increase of glucose uptake via upregulating "glucose transporters and stimulation hexokinase activity" upon AKT activation. In our previous work, we showed the increased glycolysis, PPP and TCA in human with Aldob deficiency and Aldob KO mice. Our mechanism is precisely the same as the reviewer stated. In the revision, we measured glucose consumption at different time points. As shown in ***Fig***

3E, we found that glucose levels were pronouncedly reduced in the culture medium of the cells incubated under these conditions. Interestingly, Huh7-Vector cells depleted glucose from the culture medium more efficiently than Huh7-Aldob cells. Furthermore, inhibition of Akt activity by MK2206 significantly inhibited glucose consumption and mitigated Aldob overexpression-induced inhibitory effects on glucose consumption (***Fig 3E***). Conversely, knockdown of Aldob via siRNA markedly increased glucose consumption, and MK2206 treatment also reversed the promoting effects of Aldob knockdown in Huh7-Aldob cells (***S4E and F Fig***). Consistently with these results, knockdown of endogenous Aldob by esi-Aldob in Huh7 cells significantly promoted tumor cell proliferation, glucose consumption, lactate production and Akt activation (***S3A-D Fig*** and ***Fig 7A-D***). Moreover, either Akt inhibition using MK2206 or PP2A activation with SMAP reversed Aldob knockdown-induced tumorigenic effects (***S3A-D Fig*** and ***Fig 7A-D***). All these data have further solidified our previous conclusion that Aldob suppresses glucose uptake and metabolic flux to glycolysis and TCA cycle through inhibition of the Akt signaling in HCC.

Furthermore, we found that loss of Aldob in the liver tumor tissues resulted in elevated expression levels of pT308-Akt, pS473-Akt and mTORC1 downstream target pT389-S6K (***S1B Fig***). ***It is noteworthy that the expression of HK1 and HK2 was significantly increased in ALDOB KO mice, while HK4 expression was decreased.*** Previous study have reported that HCC cells are metabolically distinct from normal hepatocytes by expressing the high-affinity hexokinase (HK1 and HK2) and suppressing glucokinase (HK4) for the accelerated glucose metabolism [9]. HK1 is expressed ubiquitously in adult tissues and is considered the housekeeping isoform of hexokinase, while HK2 is a more regulated form expressed in few adult tissues, including skeletal and cardiac muscle and adipose tissues [10], but it is highly expressed in many fetal tissues and in cancer cells. HK4, or glucokinase (GCK), is expressed primarily in the liver and pancreas [11]. HK1-2 are high-affinity hexokinases with low Km, whereas HK4 is a low affinity hexokinase with a high Km. Hexokinases share high-sequence homology but differ in their kinetics, subcellular distribution, and

regulation suited to their specific metabolic functions [11]. Akt/mTOR signaling has been shown to activate HK1 and HK2 for the induction of glucose uptake and glycolysis [12, 13]. Downregulation of hepatic HK1 and HK2 leads to impairment of glucose uptake and HCC cell proliferation [9, 14, 15]. Together, our results suggest that loss of Aldob facilitates glucose uptake and metabolic flux through upregulation of HK1 and HK2 to sustain tumor growth. These data were included in the ***Fig 3E***, ***Fig 7A-D***, ***S1B Fig***, ***S3A-D Fig***, ***S4E and F Fig***. We revised the text and added the reference accordingly. We have quantified and statistically analyzed the western blot results in ***Fig 2A***, and these data were included in the Excel file named "***Raw Data***".

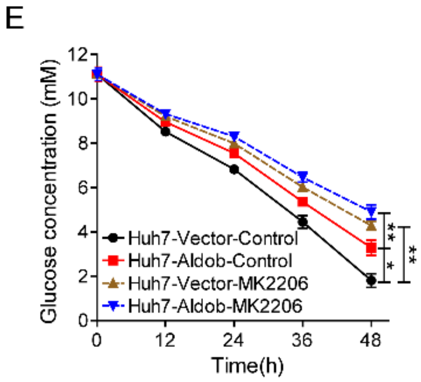

Fig 3. (E) Glucose levels in the culture medium of Huh7-Vector and Huh7-Aldob cells after treatment with DMSO or MK2206 (5 μM) at different time points.

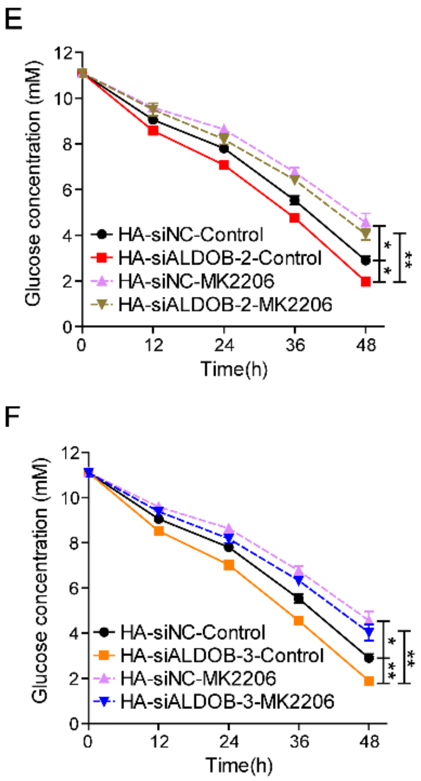

S4 Fig. (E and F) Glucose levels in the culture medium of Huh7-Aldob cells transfected with indicated siRNAs after treatment with DMSO or MK2206 (5 μM) at different time points.

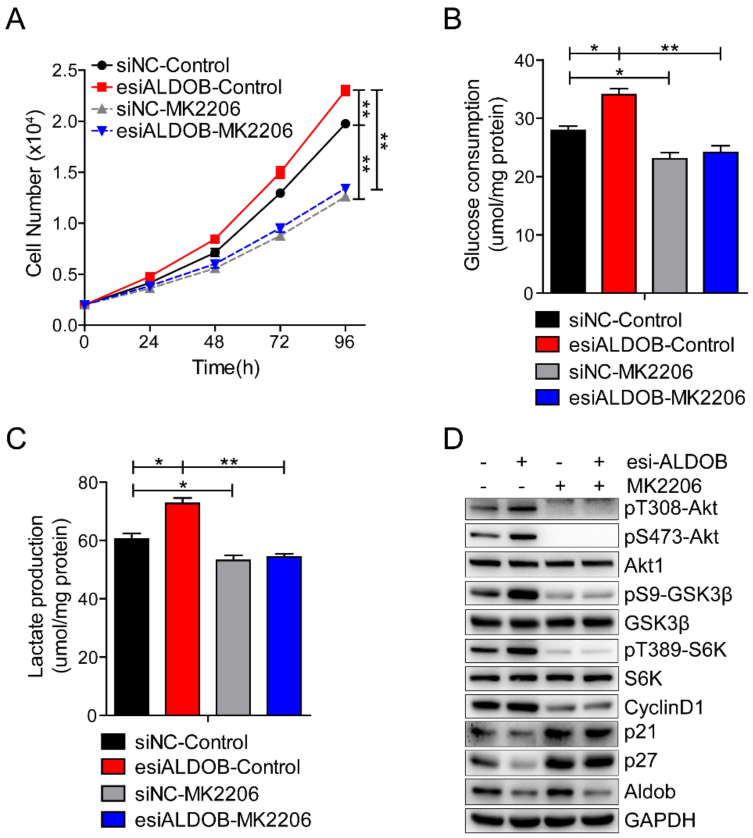

S3 Fig. (A) Cell viability of Huh7 cells transfected with indicated siRNAs in the presence of DMSO or MK2206 (2 μM). **(B and C)** Glucose consumption **(B)** and lactate production **(C)** of Huh7 cells transfected with indicated siRNAs after treatment with DMSO or MK2206 (5 μM). **(D)** IB analysis of WCL derived from Huh7 cells transfected with indicated siRNAs in the presence of DMSO or MK2206 (2 μM). Data are presented as mean ± SEM. * $p < 0.05$; ** $p < 0.01$ (Student's t-test).

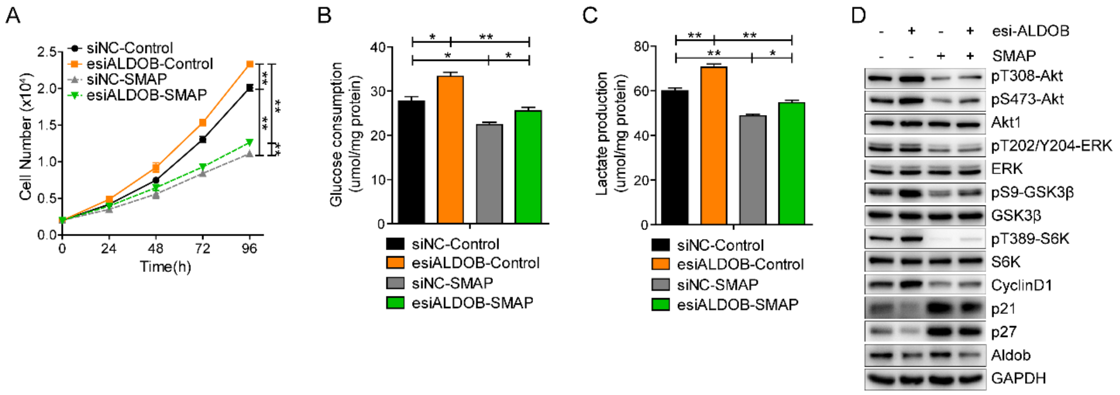

Fig 7. (A-D) Huh7 cells were transfected with esiRNA for knockdown of endogenous Aldob, and cell proliferation **(A)**, glucose consumption **(B)**, lactate production **(C)** and the expression levels of Akt signaling pathway factors **(D)** were determined in the presence of control DMSO or SMAP (10 μM).

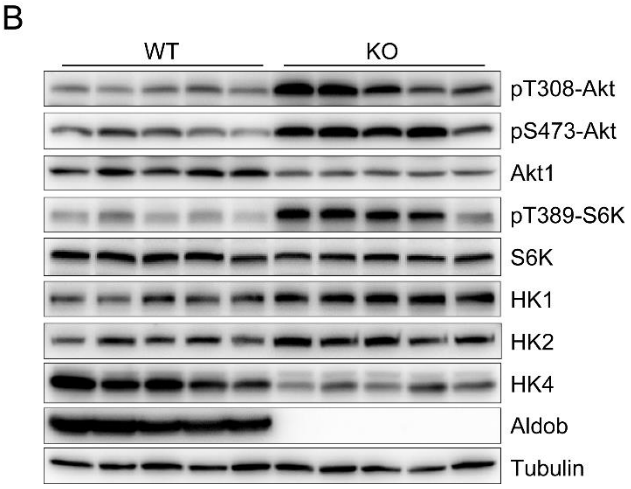

S1 Fig. (B) IB analysis of WCL derived from liver tumor tissues of WT and *ALDOB* KO mice after injection with DEN at post-natal day 14 to induce hepatocellular carcinoma for 10 months.

In the same direction, the metabolomic data presented in Fig3 does not address the questions raised. All that is shown is a general reduction in metabolites from glycolysis and the TCA upon Aldob re-expression or Akt inhibition. This reduction is across the board and in any case does not resolve the question as to how to reconcile Akt activation and increased glucose uptake with loss of aldolase activity. The authors should re-analyse their data to look for an increase in PPP intermediates to see if glucose is indeed diverted into the PPP.

Response: We thank the reviewer for raising this concern. In the previously published study, we provided compelling evidence that loss of Aldob led to a novel mode of metabolic reprogramming by which upregulating G6PD activity and PPP is able to bypass the glycolysis pathway due to the loss of Aldob [6]. We found that Aldob expression significantly inhibits G6PD activity and reduced oxidative PPP metabolites G6P, 6PG and R5P, but not non-oxidative PPP metabolites S7P and E4P (***Fig.1k-l***). Untargeted metabolomics analysis in liver tumor tissues from *ALDOB* KO and WT mice showed that oxidative PPP metabolites 6PG and R5P, but not non-oxidative PPP metabolites S7P and E4P, were significantly increased in Aldob KO mice (***Fig. 2f***), consistently with increased G6PD enzyme activity and GSH/oxidized glutathione (GSSG) ratios (***Fig. 2g and Extended Data Fig. 3j-l***). Furthermore, liver-specific *ALDOB* KO clearly increased, whereas restoration of Aldob suppressed oxidative PPP (6PG and R5P) (***Fig. 2m***), consistently with ratios of NADPH/NADP+ and GSH/GSSG (***Extended Data Fig. 4f, g***). In addition, the entire central carbon metabolism, including glycolysis, TCA and PPP, was also significantly altered (***Extended Data Fig. 4h***). Liver-specific *ALDOB* KO significantly enhanced the metabolites levels of glycolysis, TCA and PPP, such as G6P, F6P, DHAP and lactate, which were inhibited by restoration of Aldob (***Extended Data Fig. 4h***). Next, we used [1,2-$^{13}C_2$]glucose, [3-^{2}H]glucose, [1-^{2}H]glucose, and [U-$^{13}C_6$]glucose to track the metabolic flux to PPP, and found that Aldob inhibited oxidative PPP metabolism and decreased G6P, 6PG and R5P (***Fig. 6a-e and Extended Data Fig. 8a-f***).

Our recent study have strongly demonstrated that loss of Aldob in tumor cells increases G6PD activity and oxidative PPP metabolism to meet the bioenergetics and biosynthetic demands during HCC progression [6]. On this basis, our current work provides a novel insight into how loss of Aldob results in increased glucose uptake and entire central carbon metabolism, that is, loss of Aldob restores oncogenic Akt activation, thus leading to enhanced glucose uptake and metabolic flux to glycolysis and TCA cycle through upregulation of HK1 and HK2. Aldob overexpression suppresses Akt activity and reduces glucose uptake and utilization, resulting in

decreased metabolites levels of glycolysis and TCA cycle. We included this point in the discussion and added the reference accordingly.

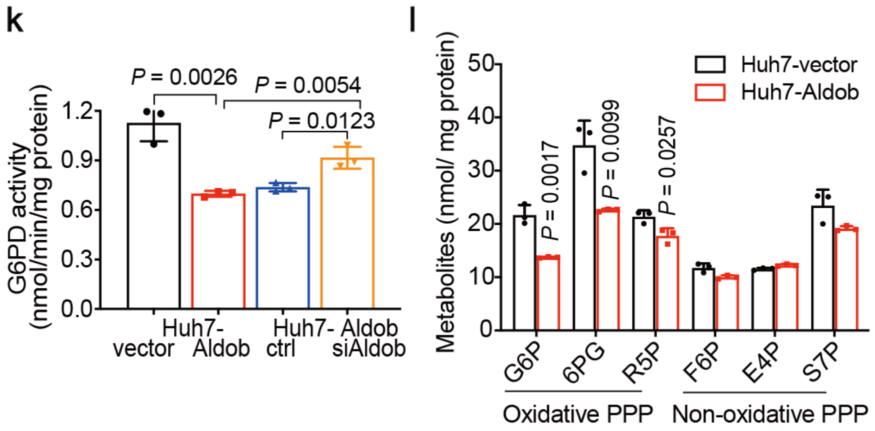

Fig. 1. k, Genetic perturbation of Aldob expression on G6PD activity by the exogenous expression or knockdown (*n* = 3 biologically independent samples). ctrl, control. **l**, Metabolite levels in PPP in Aldob overexpression cells analyzed by liquid chromatography–mass spectrometry (LC–MS) (*n* = 3 biologically independent samples). [6]

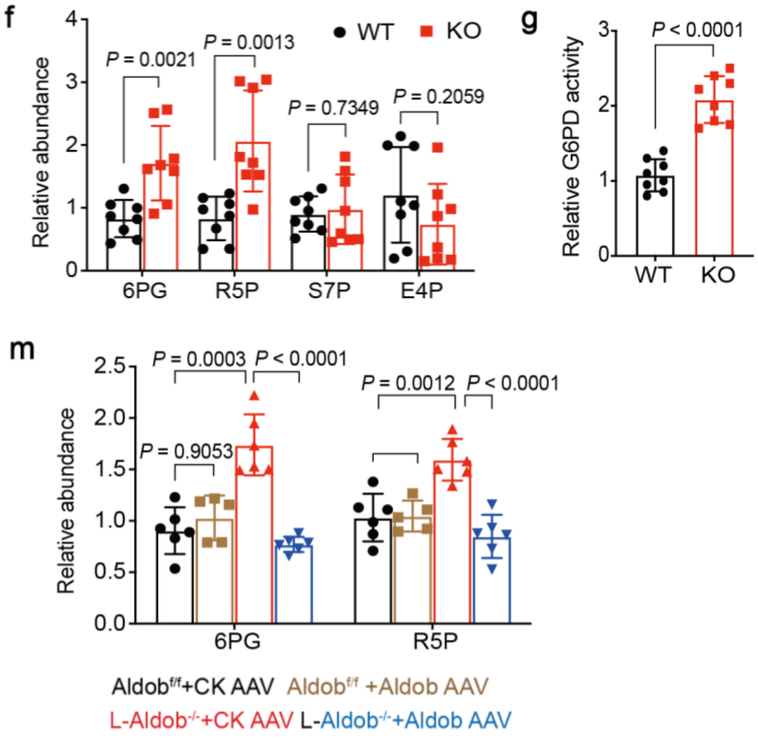

Fig. 2. f, Relative abundance of PPP metabolites in WT and KO mice at 40th week (*n* = 8 male mice per group). **g**, Relative G6PD enzyme activity in WT and Aldob KO mice (*n* = 8 male mice per group). Restoration of Aldob in L-Aldob−/− mice attenuates tumor formation. **m**, relative abundance of PPP metabolites in mice liver tissues in Aldob$^{f/f}$ + CK AAV (*n* = 6 male mice), Aldob$^{f/f}$ + Aldob AAV (*n* = 5 male mice), L-Aldob$^{-/-}$ + CK AAV (*n* = 6 male mice) and L-Aldob$^{-/-}$ + Aldob AAV (*n* = 6 male mice) groups. [6]

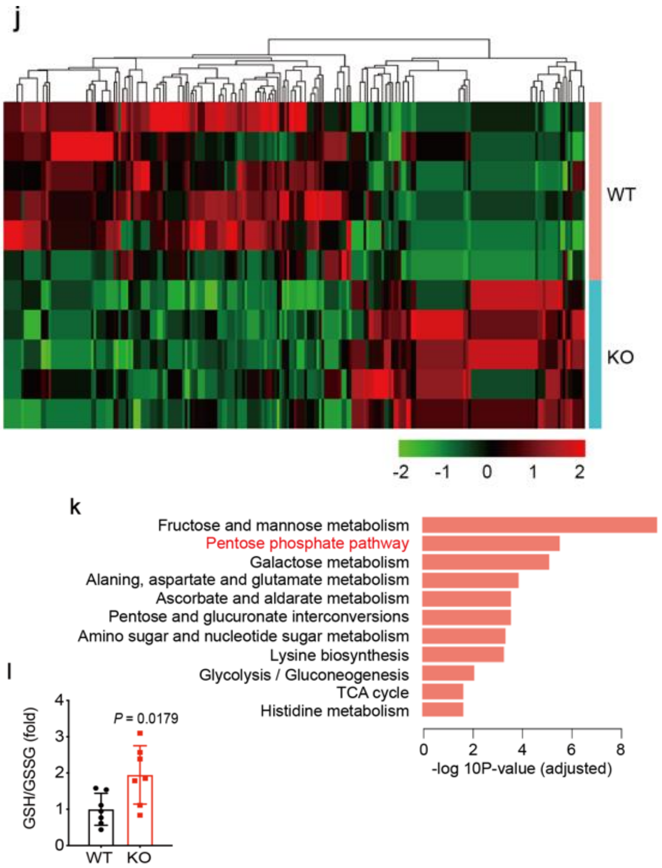

Extend Data Fig. 3. **j**, Differential metabolite analysis using untargeted metabolomics on WT and Aldob KO mice tumor at the age of 40th week by Triple TOF 6600 MS (*n* = 6 WT mice, *n* = 5 KO mice). **k**, Pathway enrichment analysis in metabolomics (*n* = 6 WT mice, *n* = 5 KO mice). **l**, Relative GSH/GSSG ratios using LC-MS (*n* = 7 male mice per group). [6]

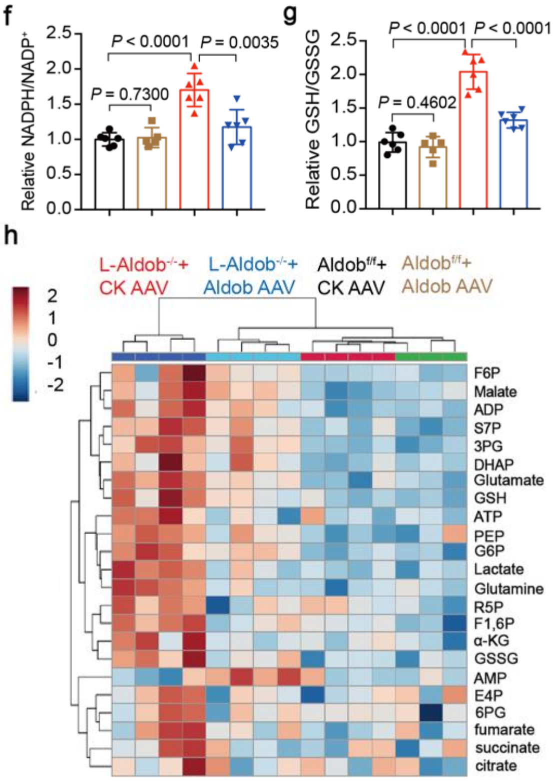

Extend Data Fig. 4. **f-g**, Relative NADPH/NADP+ ratios (**f**) and GSH/GSSG ratios (**g**) analyzed by targeted LC-MS/MS. Black: Aldob$^{f/f}$ + CK AAV (*n* = 6 male mice), Brown: Aldob$^{f/f}$ + Aldob AAV (*n* = 5 male mice), Red: L-Aldob$^{-/-}$+CK AAV (*n* = 6 male mice), Blue: L-Aldob$^{-/-}$ + Aldob AAV (*n* = 6 male mice). **h**, Heatmap of the changes of metabolites in four groups of mouse liver tumor tissues by LC-MS, Aldob$^{f/f}$ + CK AAV (*n* = 4 male mice), Aldob$^{f/f}$ + Aldob AAV (*n* = 3 male mice), L-Aldob$^{-/-}$+CK AAV (*n* = 4 male mice), L-Aldob$^{-/-}$ + Aldob AAV (*n* = 4 male mice). Heatmap is generated in MetaboAnalyst (https://www.metaboanalyst.ca) using metabolites abundance after log transformation. [6]

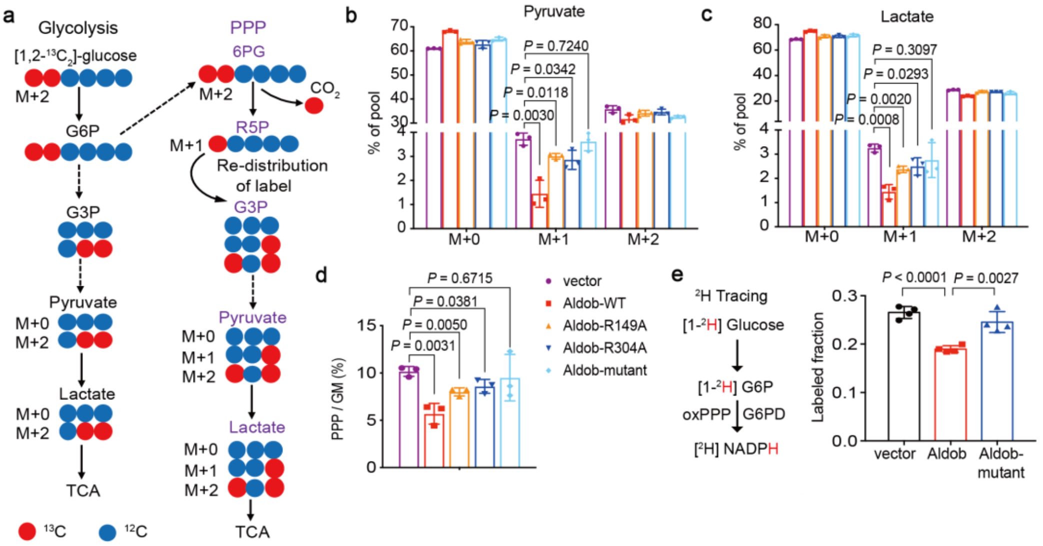

Fig. 6. a, Schematic diagram of the fate of 13C-label from [1,2-$^{13}C_2$]glucose in glycolysis and PPP. **b**,**c**, Isotopomer distributions of pyruvate (**b**) and lactate (**c**) from [1,2-$^{13}C_2$]glucose in Huh7 cells after vector, flag-Aldob, flag-Aldob-R149A, flag-Aldob-R304A and flag-Aldob-mutant plasmid transfection (cells in 2 g/L [1,2-$^{13}C_2$]glucose DMEM for 3 h; other nutritional components are the same as those in regular DMEM, *n* = 3 biologically independent samples). **d**, Percentage of PPP/glucose metabolism (GM) of lactate in Huh7 cells after vector, flag-Aldob, flag-Aldob-R149A, flag-Aldob-R304A and flag-Aldob-mutant plasmids transfection (*n* = 3 biologically independent samples). PPP/GM ratio is calculated by dividing the amount of isotopomer generated via oxidative PPP alone (M + 1) by the sum of isotopomers. GM = (M + 1)+(M + 2), which is produced from the combined activities of glycolysis and PPP. **e**, Labeled NADPH fraction from 100% [1-^{2}H] glucose (2 g/L) using a TripleTOF platform in stable Huh7-vector, Huh7-Aldob, Huh7-Aldob-mutant cell lines (*n* = 4 biologically independent samples). The time of labeling was 1 h. [6]

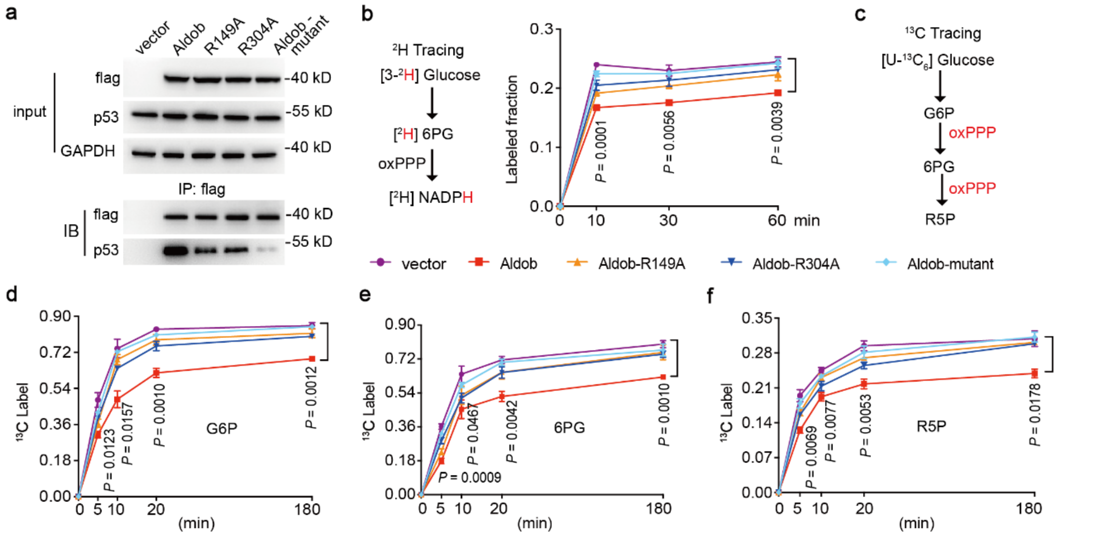

Extend Data Fig. 8. **a**, IP experiments are performed to detect the interactions between Aldob and p53 after transfection of flag Aldob-WT, flag Aldob-R149A, flag Aldob-R304A and flag Aldob-mutant plasmids. These cells are also used in b-f. **b**, Labelled NADPH fraction from 100% [3-^{2}H] glucose (2 g/L [3-^{2}H] glucose) using a TripleTOF 6600 MS platform (*n* = 3 biologically independent samples). **c-f**, Schematic diagram and labelled PPP metabolites G6P, 6PG, R5P from 100% [U-^{13}C$_6$] glucose (2 g/L [U-^{13}C$_6$] glucose) using a TripleTOF 6600 platform (*n* = 3 biologically independent samples). [6]

4. Minor points

1) Fig 1b. No controls shown for the IHC from the HCC patients. Staining from the adjacent non-malignant tissue should be shown.

Response: We thank the reviewer for this suggestion and have added the representative staining images of the adjacent non-malignant tissue in ***Fig. 1B***.

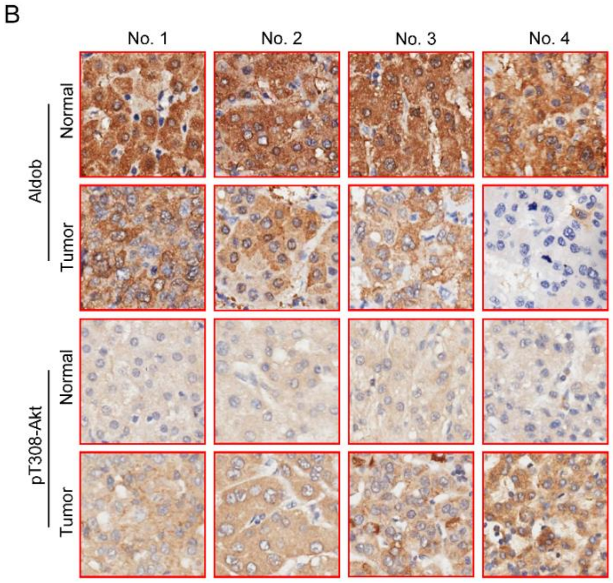

Fig 1. (B) Representative Immunohistochemistry (IHC) staining images of Aldob and pT308-Akt from the same HCC patients on tissue microarray were shown (original magnification ×200).

Reviewer #2:

1. The authors refer several times to their own previous work in which loss of Aldob promotes DEN-induced HCC in an ALDOB KO mouse model. However, there is no reference for this work along the manuscript and I have not been able to find that study across the literature. Can the authors explain that? Are those unpublished data? This should be clarified and we should have access to those data, since this is the basis for this study and a key point for the relevance of the data presented in this manuscript.

Response: We are sorry for the inconvenience and our previous manuscript was under revisions in *Nature Cancer* when we submitted this manuscript. In fact, we included the previous work by Li et al [6] as "Supporting Information". This manuscript has been published in *Nature Cancer* in July this year and we added the reference as ***ref. 18*** in the revision.

2. Authors conclude that AKT inhibition suppresses the tumorogenesis caused by loss of Aldob. This is not fully demonstrated here since they use an overexpression model for AldoB on AldoB deficient cell lines, in which AKT inhibition or SMAP-mediated PP2A activation mimics the effect of Aldob expression. Authors should limit that conclusions, or perform additional experiments to support that conclusion, that is, a loss of function model, such as the DEN-induced HCC model in the ALDOB KO mouse referred in the manuscript (see above), in which AKT inhibition (and SMAP treatment) should rescue the tumorogenesis induced by Aldob loss according to their model.

Response: We thank the reviewer for pointing out the limitation of our previous study by using overexpression models. So we used the esiRNA targeting human ALDOB (EHU001201, Sigma-Aldrich) to silence the ***endogenous*** expression of Aldob in Huh7 cells. In fact, Huh7 cells at baseline express moderate levels of Aldob among multiple HCC and PVTT (Portal Vein Tumour Thrombus) cell lines (**Figure I. A**). The endogenous Aldob expression of Huh7-Vector cells in western blots results of previous ***Fig 2C***, ***Fig 3D*** and ***Fig 7H*** appears low due to the high efficiency of Aldob overexpression with short exposure time in WB analysis (**Figure I. A**).

As shown in ***S3A Fig***, the cell viability of Huh7 cells transfected with esiRNA targeting Aldob were significantly enhanced as compared to siNC cells. Moreover,

knockdown of Aldob markedly promoted glucose consumption and lactate production (***S3B and C Fig***). Western blot analysis showed esiRNA-mediated silencing of Aldob expression significantly increased pT308-Akt, pS473-Akt, pS9-GSK3β, pT389-S6K and CyclinD1 expression, but reduced the levels of cell cycle inhibitors p21 and p27, suggesting that knockdown of Aldob promotes activation of Akt signaling in regulating cell cycle progression (***S3D Fig***). More importantly, inhibition of Akt activity by MK2206 significantly mitigated Aldob knockdown-mediated tumorigenic effects (***S3A and D Fig***). Furthermore, PP2A activation with SMAP not only inhibited glucose consumption and lactate production but also reversed Aldob knockdown-mediated above-mentioned oncogenic properties (***Fig 7A-D***). All these data support our original conclusion that downregulation of Aldob promotes HCC cell proliferation through activation of Akt signaling, and Akt inhibition or PP2A activation suppresses the tumorigenic effects driven by Aldob deficiency. We included these data in the ***S3A-D Fig*** and ***Fig 7A-D***, and revised the text accordingly. Lastly, we gathered compelling evidence to support our conclusion and it is likely out of the time frame for the revision by using the DEN-induced HCC model in the ALDOB KO mouse as suggested by the reviewer.

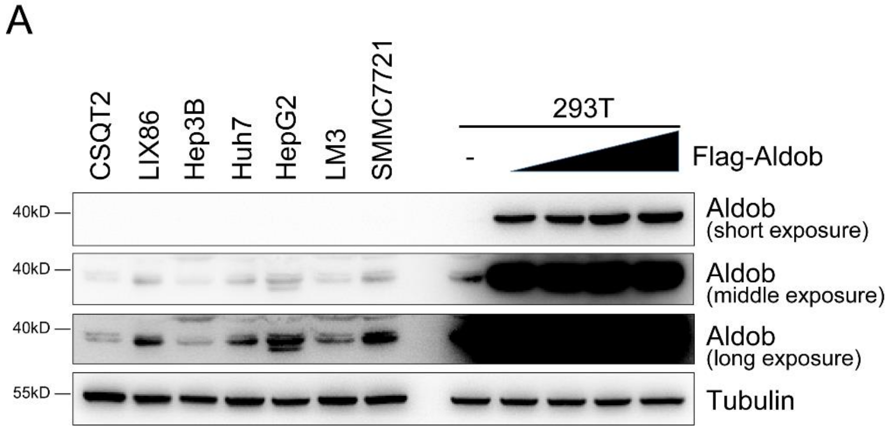

Figure I. (A) IB analysis of WCL derived from various HCC and PVTT (Portal Vein Tumor Thrombus) CSQT2 cell lines, as well as 293T cells transfected with increasing dose of Flag-Aldob constructs. The primary tumor cells LIXC086 were isolated from primary liver tumor tissues of HCC patients. (Data not shown in this reversion)

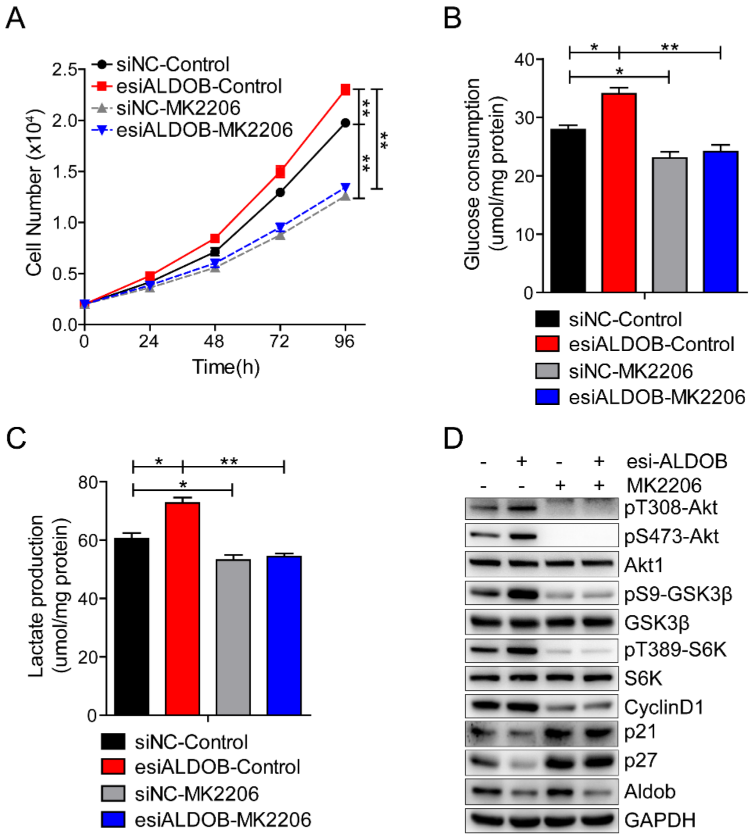

S3 Fig. (A) Cell viability of Huh7 cells transfected with indicated siRNAs in the presence of DMSO or MK2206 (2 μM). **(B and C)** Glucose consumption **(B)** and lactate production **(C)** of Huh7 cells transfected with indicated siRNAs after treatment with DMSO or MK2206 (5 μM). **(D)** IB analysis of WCL derived from Huh7 cells transfected with indicated siRNAs in the presence of DMSO or MK2206 (2 μM). Data are presented as mean ± SEM. * $p < 0.05$; ** $p < 0.01$ (Student's t-test).

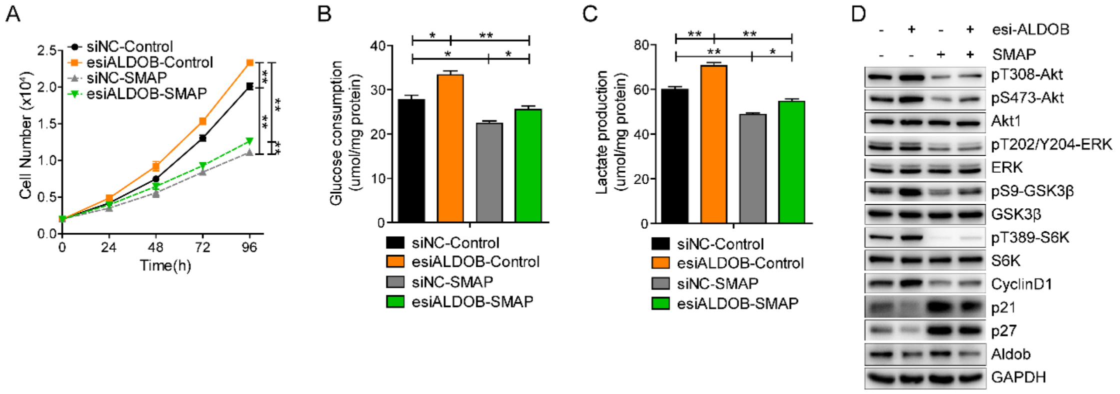

Fig 7. (A-D) Huh7 cells were transfected with esiRNA for knockdown of endogenous Aldob, and cell proliferation **(A)**, glucose consumption **(B)**, lactate production **(C)** and the expression levels of Akt signaling pathway factors **(D)** were determined in the presence of control DMSO or SMAP (10 μM).

3. PP2A is a multimeric enzyme and form multiple complexes with different regulatory subunits. Therefore, the recruitment of PP2A could be mediated through its catalytic

component but also through a specific regulatory subunit. Indeed, one of this B subunit, B55a, has been related to AKT dephosphorylation. All assays shown in the manuscript are based on the catalytic subunit of PP2A. Have the authors evaluated the presence of B55a or any other B regulatory subunit in the complex? At least some comments on the potential specific PP2A complexes involved in this new mechanism of regulation should be added to the discussion.

Response: We thank the reviewer for this comment to evaluate the presence of B55a or other B regulatory subunit in complex. PP2A is a large family of serine/threonine phosphatases, predominantly found in cellular systems in the form of heterotrimeric holoenzyme consisting of a variable regulatory subunit (B) and dimeric core enzyme formed by a structural subunit (A) and a catalytic subunit (C) [16]. The type of B subunit bound to the core dimer determines both the substrate specificity and cellular localization of PP2A holoenzyme complexes [17]. As shown in ***S7C Fig***, IP analysis showed that Flag-Aldob pulled down Akt1 and the PP2A-C subunit in Huh7 cells, but not the PP2A-A or B55α subunits. Moreover, Aldob overexpression markedly promoted Flag-PP2A-C interactions with Akt1 and Aldob without significantly impact the interactions between Flag-PP2A-C and PP2A-A or B55α subunits (***S7D Fig***). These results suggest that PP2A-C plays an important role in Aldob-dependent targeting of the PP2A to Akt. It is worthwhile to detect the presence of other B regulatory subunit in the Aldob/Akt/PP2A protein complex in future studies. These data were included in the ***S7C and D Fig***. We included this point in the discussion and revised the text accordingly.

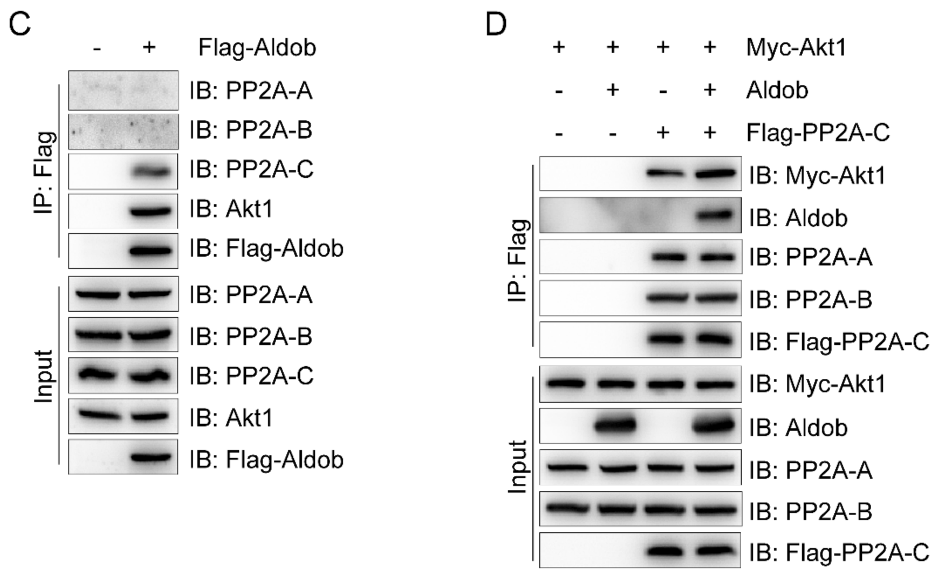

S7 Fig. **(C** and **D)** IB analysis of Flag-IP and WCL derived from Huh7 cells transfected with the indicated constructs.

4. Figure 1: The intensity scoring system used to stratify the expression of pAkt and Aldob is not clear. In methods section two different scoring systems are mentioned, this is confusing, and it is not clear whether the same scoring system was used for both markers.

Response: We are sorry for the confusion in the scoring systems and we made clarification in the revision. The former IHC intensity scoring system mentioned in Methods section is used for the analysis of tissue microarray (TMA) in ***Fig 1B***, whereas the latter is used for the Ki67 quantification in ***S6A and S8E Fig***. For the TMA, A Leica APERIO AT TURBO instrument was used for scanning and analysis. Protein expression intensity was stratified into negative (0-20 score), weak (21-100 score), moderate (101-180 score), strong (181-255 score). The calculation formula was (weak + moderate + strong)/ (negative + weak + moderate + strong) × 100%. Quantification of IHC staining was performed blindly. TMA antibody characterization of Aldob and pT308-Akt was shown in ***S1A Fig***. We revised the text accordingly.

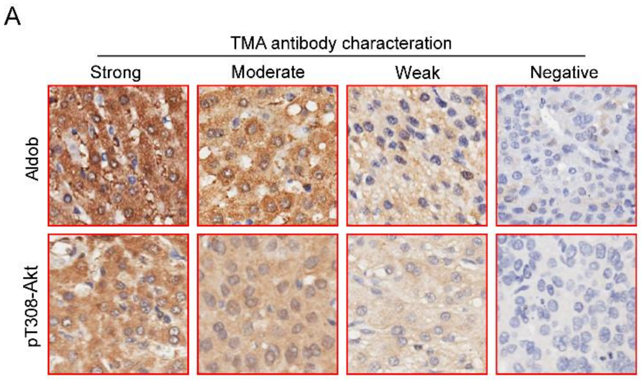

S1 Fig. (A) Tissue microarray (TMA) antibody characterization of Aldob and pT308-Akt (original magnification ×200).

5. Figure 2: Details should be provided on the ALDOB KO mouse model. Is it inducible or constitutive? Which promoter uses? This is not referenced in the text nor even mentioned in the methods section. On Fig 2A, for instance, if deletion is performed in vitro upon Cre expression, expression of Cre on ALDOB+/+ hepatocytes should be included as a control for Cre-mediated effects. In Figure 2B, are those extracts derived from liver tumors or healthy tissue? At which time those tissues were collected? The authors mention they used tissues from a KO model in which HCC was induced by DEN injection but no figure showing these data is provided. I assume this refers again to their previous study, which has not been referenced in the manuscript.

Response: We are sorry for missing this critical information regarding our Aldob KO mice, which was described in detail in our recent publication [6]. The genetically engineered mouse models mentioned in ***Fig 2A and 2B*** are constitutive deletion of Aldob. The promoter is CMV. On ***Fig 2A***, we directly isolated the primary hepatocytes from the livers of male liver-specific ALDOB-knockout (L-Aldob$^{-/-}$) and Aldob$^{flox/flox}$ (Aldob$^{f/f}$) mice for the western blot analysis. Aldob deletion is not performed in vitro upon Cre expression. Aldob$^{f/f}$ mice [6] were generated at Cyagen, using a targeting construct, which contained loxP sites flanking exons 2 and 3, followed by a loxP-flippase recognition target (FRT) site and a neomycin cassette inserted between exons 3 and 4. The targeting vector was electroporated into C57BL/6 Bruce4 embryonic stem cells. The correct recombined embryonic stem cell colony was then injected into C57BL/6 blastocysts. Male chimeras were mated with female C57BL/6 mice to obtain mice with a targeted *Aldob* allele. Mice were crossbred with C57BL/6 flp-recombinase

mice to remove the neomycin cassette to create heterozygous Aldob$^{f/f}$ mice. The mice were then crossbred with C57BL/6 mice for seven generations before being bred with heterozygous Aldob$^{f/f}$ mice to get the Aldob$^{f/f}$ mice. To generate L-Aldob$^{-/-}$ mice, we crossed Aldob$^{f/f}$ mice with *Alb*-Cre mice; mouse genotyping was performed by PCR and running DNA gel. In ***Fig 2B***, these extracts were derived from liver tumors of WT and global *ALDOB* KO mice. *ALDOB* KO and WT mice were injected with a single dose of DEN (40mg per kg body weight) at post-natal day 14. These mice were sacrificed at the age of 40 weeks, and mouse liver tumor tissues and plasma were collected. The whole-body *ALDOB* KO mouse model was generated by transcription activator-like effector nuclease (TALENs) technology. A 17 bp deletion (from 176 to192) of Aldob gene destroys Aldob protein expression using TALENs technique (***Extended Data Fig. 3a***) [6].

In our recent study [6], we investigated the tumor-suppressive role of Aldob by injecting diethyl nitrosamine (DEN) into global *ALDOB* knockout (KO) mice fed with a fructose-free diet (***Extended Data Fig.3a, and Fig.2a***). At the 40th week, *ALDOB* KO mice had significantly greater ratios of liver/body weight, more tumours, larger tumor size and higher incidence of tumors >5mm (***Fig.2b-e and Extended Data Fig.3g***). IHC analysis also demonstrated that tumor proliferation marker Ki67, vascular endothelial growth factor A (VEGFA) and β-cadherin-associated protein (β-catenin) were all significantly increased in KO mice tumors (***Extended Data Fig. 3h,i***). The same results were also found in the DEN-induced HCC model in liver-specific *ALDOB* KO mice. All these data clearly demonstrate that loss of Aldob promotes liver tumorigenesis in mice. The text and methodological details were revised accordingly.

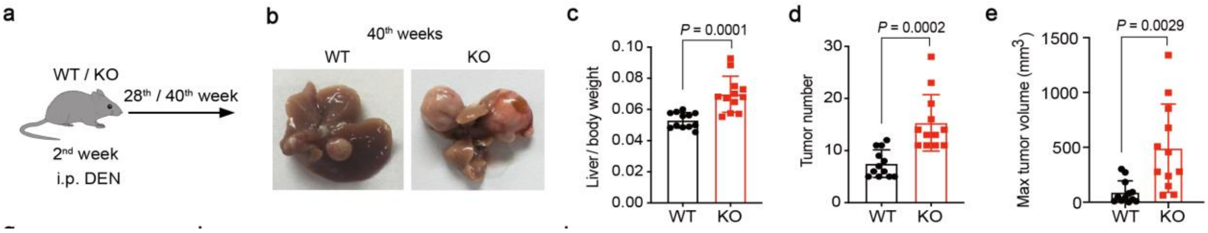

Fig. 2. Global Aldob deficiency (KO) promotes tumorigenesis (**a-e**). **a,** HCC mouse model design. **b,** Representative images of WT and global Aldob-KO liver at 40th week. **c,** Liver/body weight; **d**, Tumor numbers; **e**, Max tumor volume at 40th week (n=12 male mice per group). The formula of max tumor volume: 0.52 x length x width^{2}**.** [6]

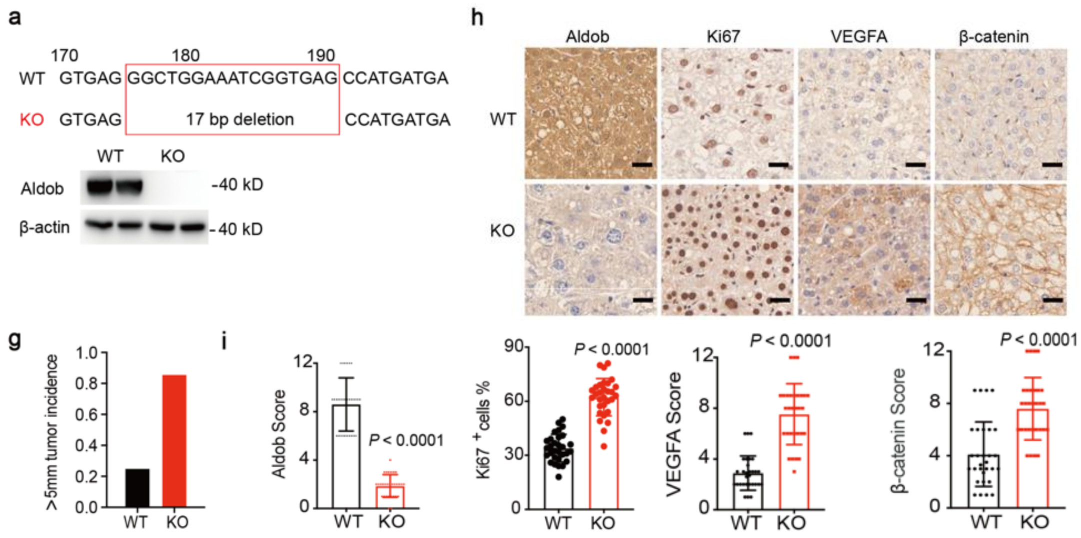

Extended Data Fig. 3. a, A 17bp deletion (from 176 to192) of Aldob gene destroys Aldob protein expression using TALENs technique, and Western Blot of Aldob expression. **g**, Incidence of tumor with >5mm in WT and Aldob KO mouse livers at 40th week (n=12 male mice per group). **h-i**, Representative images (**h**) and IHC quantification of Aldob, Ki67, VEGFA and β-catenin expressions (**i**) (n=30 IHC staining) in mouse liver at 40th week (IHC score = intensity x area. The intensity was graded as 0, 1, 2, 3. The area of positive staining was assessed by 0, 1-25%, 26-50%, 51-75%, 76-100%, corresponding to 0, 1, 2, 3, 4 grades, respectively, scale bars, 50 μm). [6]

6. Figure 3D: On panel D, the authors claim that AldoB expression similarly to AKT inhibition increases the levels of p21 and p27. However, this is not clearly seen in the figure.

Response: We performed the quantification analysis for the levels of p21 and p27 in ***Fig 3D***. We found that Aldob overexpression significantly upregulated the levels of p21 and p27 by about 2 and 1.8 folds, respectively. Akt inhibition using MK2206 significantly increased the levels of p21 and p27 by about 2.3 and 2 folds, respectively. Moreover, esiRNA-mediated silencing of endogenous Aldob expression significantly inhibited the levels of p21 and p27 by about 50% and 55%, respectively, compared to that of control group (***S3D Fig***). We have quantified and statistically analyzed all western blot results, and these data were included in the Excel file named "***Raw Data***".

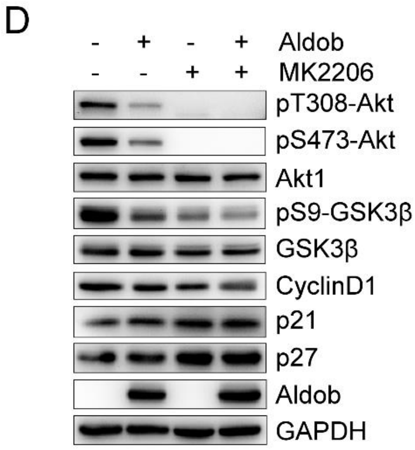

Fig 3. (D) Cells were treated with DMSO or MK2206 (2 μM) for 48 hours, and then subjected to IB analysis with the indicated antibodies **(D)**.

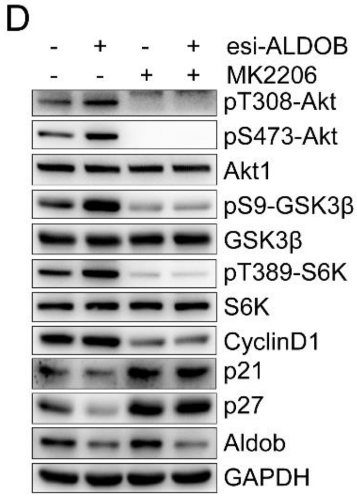

S3 Fig. (D) IB analysis of WCL derived from Huh7 cells transfected with indicated siRNAs in the presence of DMSO or MK2206 (2 μM).

7. Figure 3E-G: Aldob expression reduced both glycolysis and TCA metabolism, suggesting a general reduction in the metabolic activity of these cells. Is this caused by a reduction in glucose uptake? These data could probably be obtained from the metabolic labeling assay by monitoring the glucose consumption from the medium.

Response: We thank the reviewer for this suggestion and similar concerns were raised by Reviewer 1. We measured glucose consumption, lactate production and performed metabolic tracing experiments to show that Aldob expression indeed reduces glucose uptake and subsequent glycolysis and TCA metabolism. As shown in ***Fig 3E***, we found that glucose levels were pronouncedly reduced in the culture medium of the cells incubated under these conditions. Interestingly, Huh7-Vector cells depleted

glucose from the culture medium more efficiently than Huh7-Aldob cells. Furthermore, inhibition of Akt activity by MK2206 significantly inhibited glucose consumption and diminished Aldob overexpression-induced inhibitory effects on glucose consumption (***Fig 3E***). Conversely, knockdown of Aldob via siRNA markedly increased glucose consumption, and MK2206 treatment also reversed the promoting effects of Aldob knockdown in Huh7-Aldob cells (***S4E and F Fig***). Consistently with these results, knockdown of endogenous Aldob by esi-Aldob in Huh7 cells significantly promoted tumor cell proliferation, glucose consumption, lactate production and Akt activation (***S3A-D Fig*** and ***Fig 7A-D***). Moreover, either Akt inhibition using MK2206 or PP2A activation with SMAP reversed Aldob knockdown-induced tumorigenic effects (***S3A-D Fig*** and ***Fig 7A-D***). All these results suggest that Aldob-induced inhibition of Akt activity resulted in the reduction of glucose uptake and metabolic flux to glycolysis and TCA cycle in HCC. These data were included in the ***Fig 3E***, ***Fig 7A-D***, ***S3A-D Fig***, ***S4E and F Fig***. We revised the text and added the data accordingly.

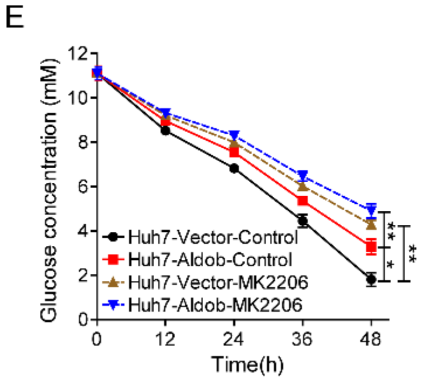

Fig 3. (E) Glucose levels in the culture medium of Huh7-Vector and Huh7-Aldob cells after treatment with DMSO or MK2206 (5 μM) at different time points.

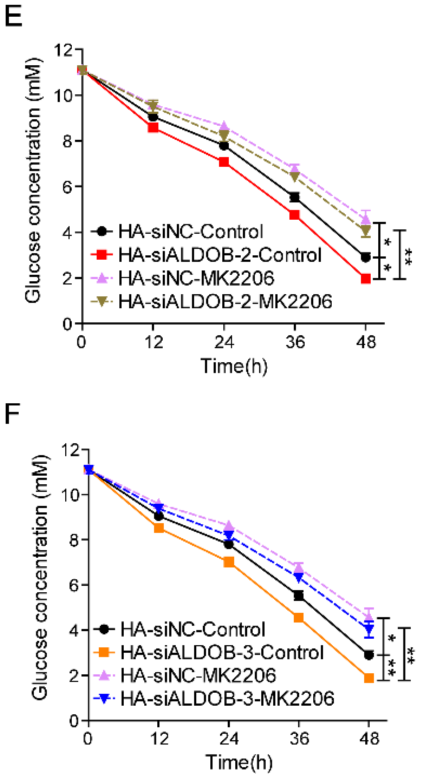

S4 Fig. (E and F) Glucose levels in the culture medium of Huh7-Aldob cells transfected with indicated siRNAs after treatment with DMSO or MK2206 (5 μM) at different time points.

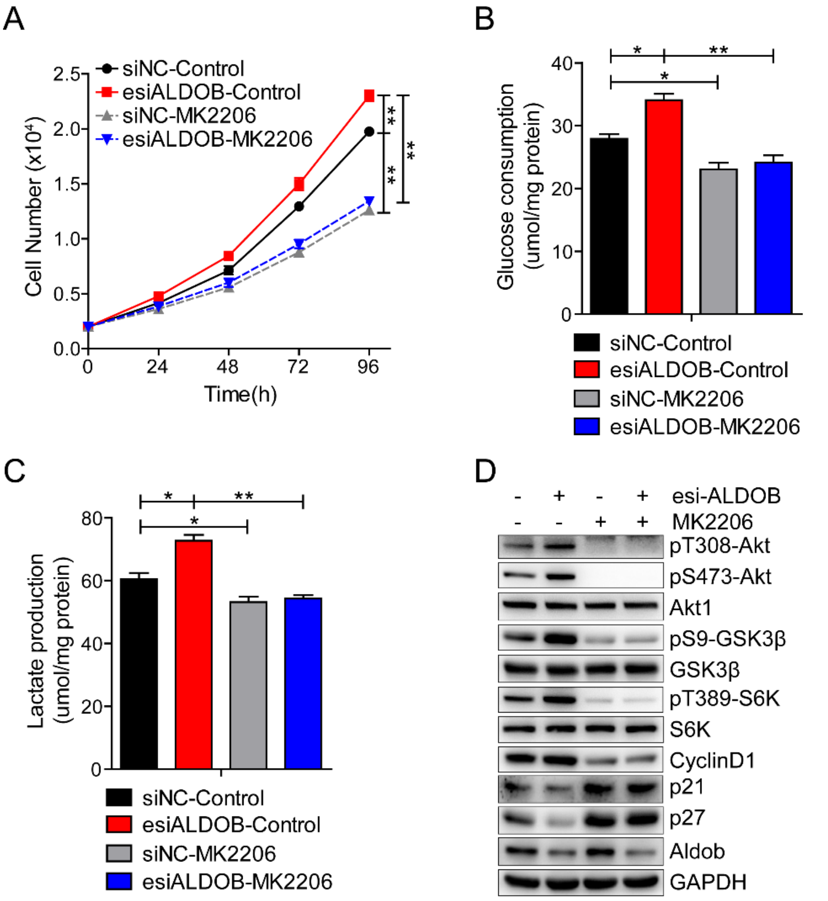

S3 Fig. (A) Cell viability of Huh7 cells transfected with indicated siRNAs in the presence of DMSO or MK2206 (2 μM). **(B and C)** Glucose consumption **(B)** and lactate production **(C)** of Huh7 cells transfected with indicated siRNAs after treatment with DMSO or MK2206 (5 μM). **(D)** IB analysis of WCL derived from Huh7 cells transfected with indicated siRNAs in the presence of DMSO or MK2206 (2 μM).

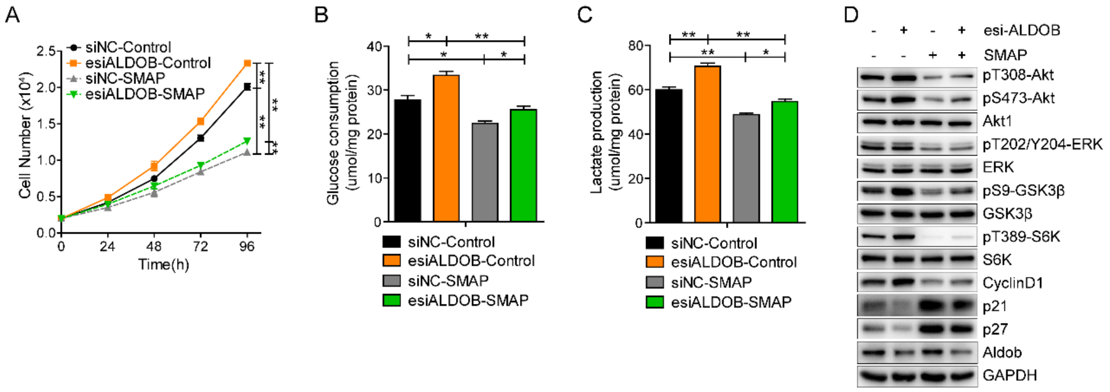

Fig 7. (A-D) Huh7 cells were transfected with esiRNA for knockdown of endogenous Aldob, and cell proliferation **(A)**, glucose consumption **(B)**, lactate production **(C)** and the protein expression levels of Akt pathway **(D)** were determined in the presence of control DMSO or SMAP (10 μM).

8. Figure 3H-J: When MK2206 was administered? At the time of subcutaneous injection or once the tumors well established. This is relevant information for the therapeutic proposal. Same for SMAP in the xenograft assay shown in Figure 7.

Response: We are sorry for missing these experimental details in our previous version. About 10 days after cell inoculation, when the tumors were visible and reached 100 mm^3, MK2206 and SMAP were administered in xenograft mouse models. We revised the text and methodological details accordingly.

9. Figure 4E: How Aldob enzymatic activity was determined? This is not indicated neither in the figure legend or in the methods section.

Response: Aldolase catalyzes the reversible reaction of fructose-1, 6-bisphosphate (F-1, 6-BP) to glyceraldehyde-3-phosphate and dihydroxyacetone phosphate. The measurement of Aldolase activity was performed using F-1, 6-BP as the substrate by Aldolase Activity Colorimetric Assay Kit (#K665-100, BioVision) according to the manufacturer's instructions. We added the methodological details into the Methods

section of the revision.

Reviewer #3:

1- The down-regulation of aldolase B gene expression in liver cancer cells is clear. However, the authors should measure in the same cancer samples the expression of multiple aldolase isoforms, which may not be expressed in normal liver but may be reactivated during tumorigenesis. This is quite common for isoforms of many other glycolytic enzymes. Total aldolase activity should be assessed in their experimental system.

Response: We thank the reviewer for the overall enthusiasm about our manuscript and raising this concern regarding the potential roles of other aldolase isoforms, especially Aldoa. Similar concerns were raised by Reviewer 1 and please also see our response. In short, we have compelling evidence to show that hepatic Aldob plays a more important role in HCC and the total aldolase activities in human HCC tissues are significantly down regulated with the loss of Aldob [6]. Aldolase family includes Aldoa, Aldob, and Aldoc, and these three isoenzymes are expressed in the specific human organs [1]. Aldoa is expressed in embryos and is abundantly available in adult muscle tissue. Aldob is the predominant isoform in liver, kidney and small intestine. Aldoc is abundant in the central nervous system. In our recent study [6], we found that Aldoa expression was slightly ***decreased***, whereas there was ***no significant change*** of Aldoc in HCC tumor tissues when compared to normal liver tissues ***(Extended Data Fig. 1g–j)***. Furthermore, Aldoa and Aldoc expression did ***not correlate*** with HCC survival ***(Extended Data Fig. 1k–m)***. Moreover, we detected total aldolase enzymatic activity in 4 pairs of human HCC samples and found that aldolase enzymatic activity was significantly ***decreased*** in tumor and PVTT (Portal Vein Tumour Thrombus) tissues as compared to matched adjacent normal liver tissues ***(Extended Data Fig. 3d)***. These results suggest that Aldob plays a key role in regulating glucose metabolism and HCC progression.

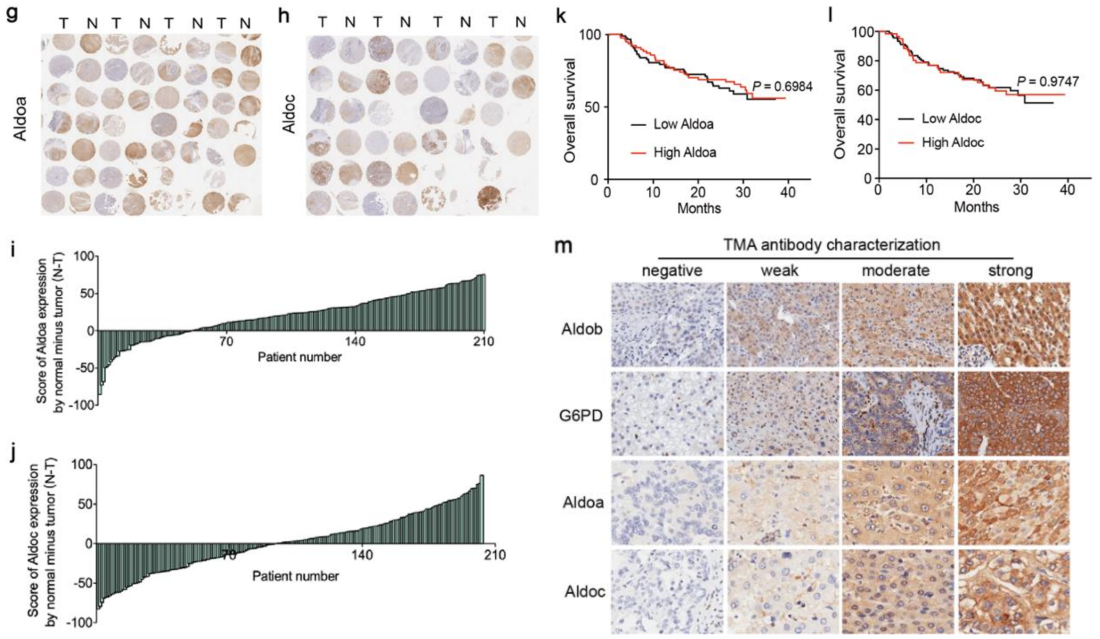

Extended Data Fig. 1. **g-h**, Representative TMA images with Aldoa (**g**) and Aldoc (**h**) antibody. i-j, Quantification of protein expression score of the matched normal tissues minus tumors from 184 HCC tissue microarray data with Aldoa (**i**) and Aldoc (**j**) antibody. Protein expression intensity is classified into negative (0-20 score), weak (21-100 score), moderate (101-180 score), strong (181-255 score). The calculation formula: (weak + moderate + strong)/(weak + moderate + strong + negative) x 100%. **k-l**, Kaplan-Meier survival curves in HCC patients with Aldoa (**k**, *n* = 214) and Aldoc (**l**, *n* = 203) high and low expression (patients are divided into two groups with high (>50) and low (< 50%) Aldoa or Aldoc expression). **m**, TMA antibody characterization of Aldob, G6PD, Aldoa, Aldoc (magnification: 200×). [6]

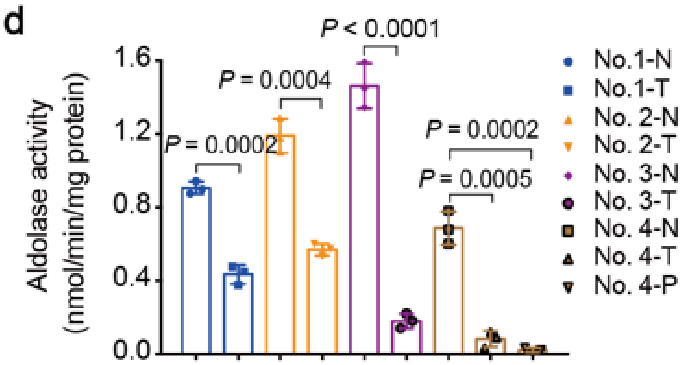

Extend Data Fig. 3. **d**, Aldolase enzymatic activity in human HCC tissues (n = 3 biologically independent samples). [6]

2- All western blots should be quantified and statistically analysed.

Response: We thank the reviewer for this suggestion. We have quantified and

statistically analyzed all western blot results, and these data were included in the Excel file named "***Raw Data***".

3- The authors show in Fig. 1 an up-regulation of S6K1 phosphorylation which is at least as important as Akt. They comment that S6K1 is an indirect target of Akt activity. They should also consider the possibility that there is a general activation of both mTORC1 and mTORC2 in these cancer cells. This can be tested by looking at the phosphorylation of additional mTORC1 and mTORC2 targets. They should also measure S6K1 phosphorylation in the experimental conditions of the following figures (i.e. pharmacological inhibition of Akt).

Response: We thank the reviewer for raising this concern and suggestions to test other phosphorylation targets of mTORC1 and mTORC2. As shown in ***S1B Fig***, Aldob knockout in the liver tumor tissues resulted in elevated expression levels of pT308-Akt, pS473-Akt and mTORC1 direct downstream target pT389-S6K1. Moreover, loss of Aldob clearly upregulated the sterol responsive element binding protein 1 (SREBP1) transcription factor (**Figure II. A**). mTORC1 promotes de novo lipid synthesis through SREBP1, which controls the expression of metabolic genes involved in fatty acid biosynthesis [18]. However, there was no significant change of the phosphorylation level of mTORC2 downstream target PKCα in liver tumor tissues from *ALDOB* KO mice when compared to WT (***S1B Fig***). These results indicate that loss of Aldob promotes the activation of Akt/mTORC1 signaling, but not the mTORC2 kinase activity.

We also used the esiRNA targeting human ALDOB (EHU001201, Sigma-Aldrich) to knockdown the endogenous expression of Aldob in Huh7 cells. As shown in ***S3D Fig*** and ***Fig 7D***, Western blot analysis showed esiRNA-mediated silencing of Aldob expression significantly increased pT308-Akt, pS473-Akt, pS9-GSK3β, pT389-S6K1 and CyclinD1 expression, but reduced the levels of cell cycle inhibitors p21 and p27, suggesting that knockdown of Aldob promotes activation of Akt signaling. More importantly, inhibition of Akt activity by MK2206 or PP2A activation with SMAP significantly reversed Aldob knockdown-mediated activation of oncogenic Akt

signaling and upregulation of S6K1 phosphorylation (***S3D Fig*** and ***Fig 7D***). Consistently, up-regulated Aldob obviously reduced pT308-Akt, pS473-Akt, pS9-GSK3β, pT389-S6K1 and CyclinD1 expression, while elevating the levels of cell cycle inhibitors p21 and p27 (***S2F Fig***). More importantly, Akt activation by ectopic expression of Myc-Akt1, but not the Myc-Akt1 phospho-deficient mutant (Myc-Akt1-AA), dramatically diminished Aldob-mediated inhibition of Akt signaling and S6K1 phosphorylation (***S2F Fig***). All these data support our original conclusion that downregulation of Aldob promotes HCC cell proliferation through activation of Akt signaling, and Akt inhibition or PP2A activation suppresses the tumorigenic effects driven by Aldob deficiency. We included these data in the ***S1B Fig***, ***S2F Fig***, ***S3D Fig*** and ***Fig 7D***, and revised the text accordingly.

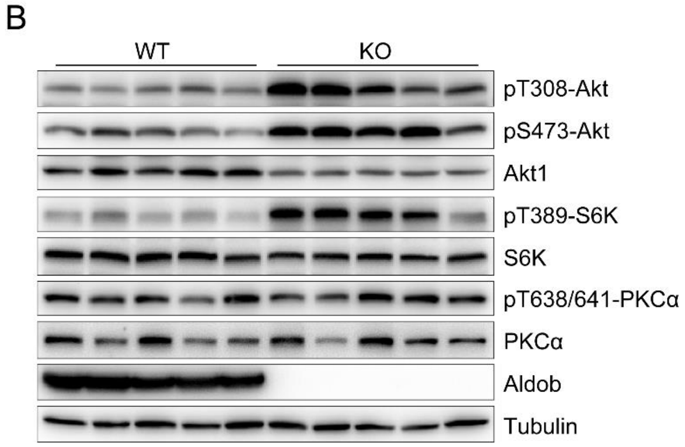

S1 Fig. (B) IB analysis of WCL derived from liver tumor tissues of WT and *ALDOB* KO mice after injection with DEN at post-natal day 14 to induce hepatocellular carcinoma for 10 months.

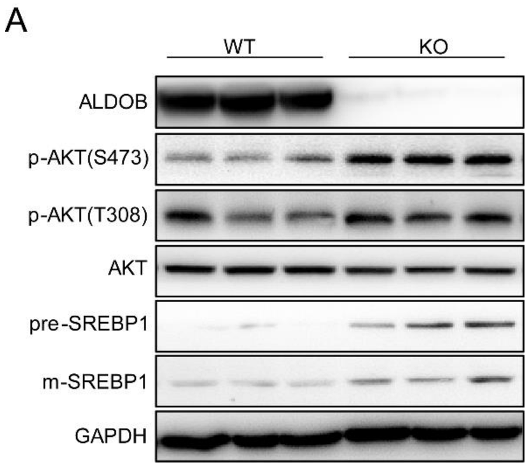

Figure II. (A) IB analysis of WCL derived from liver tumor tissues of WT and *ALDOB* KO mice

after injection with DEN at post-natal day 14 to induce hepatocellular carcinoma for 10 months. pre, precursor. m, mature. (Data not shown in this reversion)

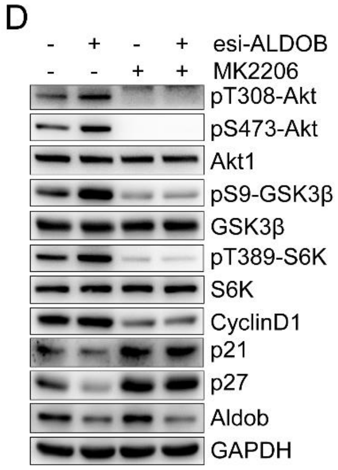

S3 Fig. (D) IB analysis of WCL derived from Huh7 cells transfected with indicated siRNAs in the presence of DMSO or MK2206 (2 μM).

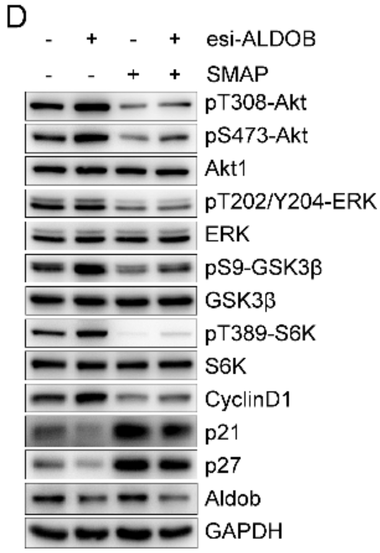

Fig 7. (D) Huh7 cells were transfected with esiRNA for knockdown of endogenous Aldob, and the expression levels of Akt signaling pathway factors were determined in the presence of control DMSO or SMAP (10 μM).

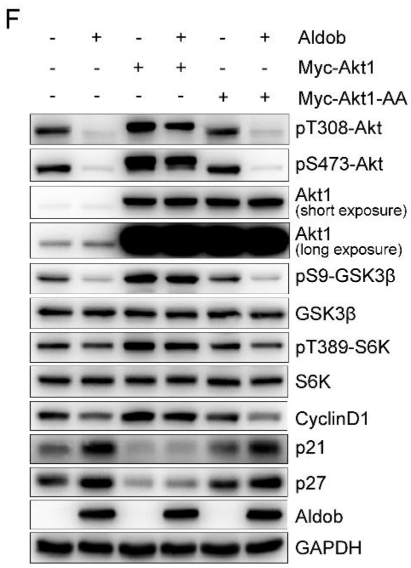

S2 Fig. (F) IB analysis of WCL derived from Huh7 cells transfected with the indicated constructs.

4- If multiple mTORC1 and mTORC2 targets are phosphorylated, the authors should consider the possibility that aldolase B may affect kinase activity in parallel of the phosphatase.

Response: We thank the reviewer for this suggestion. As we mentioned in the response to previous concern, our data support the conclusion that loss of Aldob promotes the activation of Akt/mTORC1 signaling without a significant impact on mTORC2 kinase activity.

The mechanistic target of rapamycin (mTOR) is a conserved serine/threonine protein kinase that forms two cellular complexes known as mTORC1 and mTORC2, with distinct subunit composition and substrate selectivity [19]. mTORC1 functions as a downstream effector for multiple oncogenic signaling pathways, including PI3K/Akt signaling and Ras/Raf/MEK/ERK (MAPK) signaling. In response to growth factors stimulation, activated Akt phosphorylates the tuberous sclerosis complex 2 (TSC2) and inhibits the GTPase-activating protein (GAP) activity of the tumor suppressor TSC2/TSC1 complex. TSC2 acts as a GAP specific for the GTPase Rheb and promotes the conversion of Rheb-GTP which is an essential positive regulator of mTORC1, to Rheb-GDP, thereby suppressing mTORC1. Thus Akt acts as an upstream activator of mTORC1 [18]. Similarly, ERK also activates mTORC1 via phosphorylation and

inhibition of TSC2 [20]. mTORC1 activation promotes the biosynthetic processes of protein, lipid and nucleotide to support cell growth through phosphorylating numerous downstream substrates such as S6K1, while suppresses the catabolic pathway of autophagy. mTORC1 promotes de novo lipid synthesis through SREBP1, which controls the expression of metabolic genes involved in fatty acid biosynthesis [18]. On the other hand, mTORC2 regulates cell proliferation and survival primarily by phosphorylating several members of the AGC (PKA/PKG/PKC) family of protein kinases, such as Akt and protein kinase C (PKC) isoforms [18].

As shown in ***S1B Fig*** and **Figure II. A**, loss of Aldob significantly increased pT308-Akt, pS473-Akt, pT389-S6K1 and SREBP1 expression, but had no effect on PKCα phosphorylation, suggesting that loss of Aldob enhances the kinase activities of Akt and mTORC1, but not mTORC2. Moreover, esiRNA-mediated silencing of Aldob expression significantly increased the expression of pT308-Akt, pS473-Akt and pT389-S6K1, but had no effect on ERK phosphorylation, suggesting that knockdown of Aldob promotes mTORC1 kinase activity through activation of Akt, but not ERK (***Fig 7D***). mTORC1 signaling plays an important role in promoting lipid synthesis through upregulating SREBP1. Indeed, ongoing studies will test the effect of Aldob on lipid synthesis and hepatocarcinogenesis. Our current study reports that Aldob prefers interacting with phosphorylated Akt and promotes the recruitment of PP2A to dephosphorylate p-Akt, thereby inhibiting Akt phosphorylation and kinase activity. We conclude that loss of Aldob increases mTORC1 kinase activity due to the activation of mTORC1 upstream activator Akt, but had no effect on mTORC2 which is the upstream protein kinase of Akt. We included these data in the ***S1B Fig*** and ***Fig 7D***, and discussed this point in the revision.

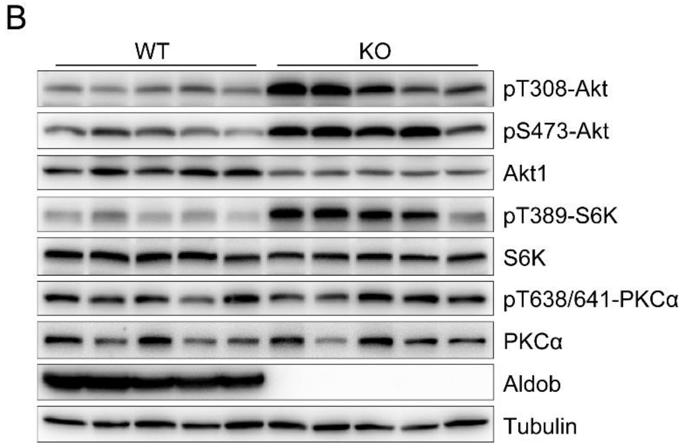

S1 Fig. (B) IB analysis of WCL derived from liver tumor tissues of WT and *ALDOB* KO mice after injection with DEN at post-natal day 14 to induce hepatocellular carcinoma for 10 months.

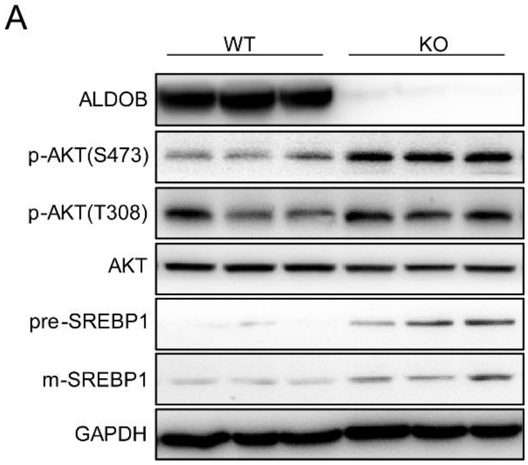

Figure II. (A) IB analysis of WCL derived from liver tumor tissues of WT and *ALDOB* KO mice after injection with DEN at post-natal day 14 to induce hepatocellular carcinoma for 10 months. pre, precursor. m, mature. (Data not shown in this reversion)

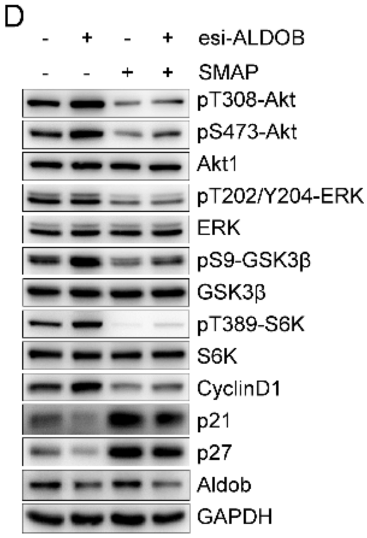

Fig 7. (D) Huh7 cells were transfected with esiRNA for knockdown of endogenous Aldob, and the expression levels of Akt signaling pathway factors were determined in the presence of control DMSO or SMAP (10 μM).

5- In the majority of the experimental data, aldolase B expression and Akt inhibition have additive effects on the functional read-outs (metabolomics, proliferation, tumour growth: Fig. 2). The authors should consider the possibility that aldolase B and Akt may be on parallel pathways regulating tumor cell growth and metabolism.

Response: We thank the reviewer for this comment and our data strongly suggest the additive effects but **not** "on parallel pathways regulating tumor cell growth and metabolism". In ***Fig 2A***, we found that in liver-specific *ALDOB* KO mouse primary hepatocytes, Aldob deletion significantly augmented Akt activation compared to WT, as evidenced by increased levels of pT308-Akt, pS473-Akt, pS9-GSK3β, pT389-S6K and HK1. Meanwhile, loss of Aldob in liver tumor tissues of *ALDOB* KO mice compared to WT mice resulted in elevated expression levels of pT308-Akt, pS473-Akt, pS9-GSK3β, pT389-S6K, HK1 and HK2 (***Fig 2B and S1B Fig***). These data were consistent with our previous work that loss of Aldob promotes DEN-induced HCC tumorigenesis in *ALDOB* KO mice [6], suggesting a potential tumor-suppressive role of Aldob through inhibition of oncogenic Akt activity. Moreover, knockdown of endogenous Aldob by esi-Aldob in Huh7 cells significantly promoted cell viability, glucose consumption and lactate production, as well as increased pT308-Akt, pS473-Akt, pS9-GSK3β, pT389-S6K and CyclinD1 expression, reduced the levels of cell cycle inhibitors p21 and p27 (***S3A-D Fig*** and ***Fig 7A-D***). More importantly, treatment with MK2206 or SMAP significantly inhibited Akt activity and effectively reversed Aldob knockdown-induced tumorigenic effects (***S3A-D Fig*** and ***Fig 7A-D***). Furthermore, overexpression of Aldob inhibited, whereas Aldob knockdown promoted Akt signaling, cell proliferation, cell cycle progression, glucose consumption and metabolic flux to glycolysis and TCA (***Fig 2C-F, Fig 3A-H and S4A-J Fig***). Besides, either Akt activation by ectopic expression of Myc-Akt1 or Akt inhibition using MK2206 weakened Aldob overexpression-induced growth inhibitory effects (***Fig 3A-H, S2A-F and S4A-J Fig***). *In vitro* Akt kinase assay also showed that overexpressed Aldob dramatically inhibited Akt phosphorylation of its substrate GSK3α (***Fig 2G***). All

these data suggest that Aldob indeed inhibits Akt activity and downstream signaling events, which is required for Aldob-induced suppression of cancer cell proliferation, glucose metabolism and tumorigenesis.

Aldolase B has been implicated in diverse physiological and pathological processes. Previous study reported that aldolase acts as a sensor of FBP and glucose availability in the regulation of AMP-activated protein kinase (AMPK) [21, 22]. Dissociation of aldolase from actin cytoskeleton leads to increased aldolase activity and enhanced glycolytic flux, which is positively regulated by PI3K signaling [23]. It has been suggested that overexpression of Aldob inhibits HCC cell migration through elevation of Ten-Eleven Translocation 1 [24]. Although our current work does not rule out the possibility of other tumor-suppressive mechanism(s) for Aldob, our current results strongly suggest that Aldob suppresses HCC cell proliferation and glucose metabolism through inhibition of Akt kinase activity.

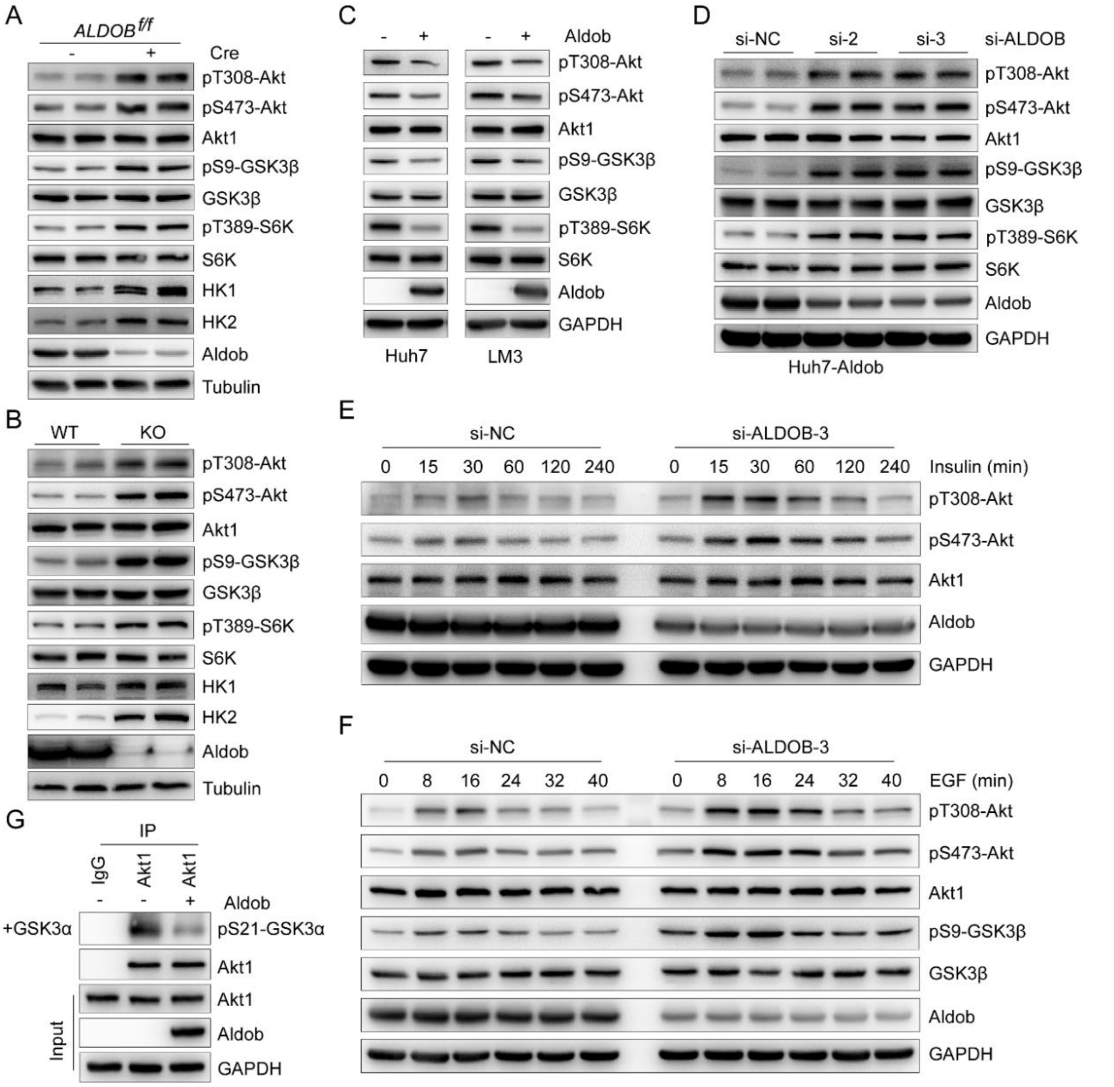

Fig 2. **(A)** IB analysis of WCL derived from conditional *ALDOB* KO and WT mouse primary hepatocytes. **(B)** IB analysis of WCL derived from liver tumor tissues of *ALDOB* KO and WT mice after injection with DEN at post-natal day 14 to induce hepatocellular carcinoma for 10 months. **(C)** IB analysis of WCL derived from Huh7 and LM3 cells stably expressing Aldob via lentiviral infection (with Vector as a negative control). **(D)** IB analysis of WCL derived from Aldob-overexpressing Huh7 (Huh7-Aldob) cells transfected with indicated siRNAs (si-NC as a negative control). **(E and F)** IB analysis of WCL derived from Huh7-Aldob cells transfected with indicated siRNAs. Resulting cells were serum-starved for 24 hours and then stimulated with 0.1 μM insulin **(E)** or 100 ng/ml EGF **(F)** at the indicated time before harvesting. **(G)** *In vitro* Akt1 kinase assay was conducted with recombinant GSK3α as a substrate and immunoprecipitated Akt1 from Huh7-Vector or Huh7-Aldob cells as the source of kinase. IgG was used as a negative control.

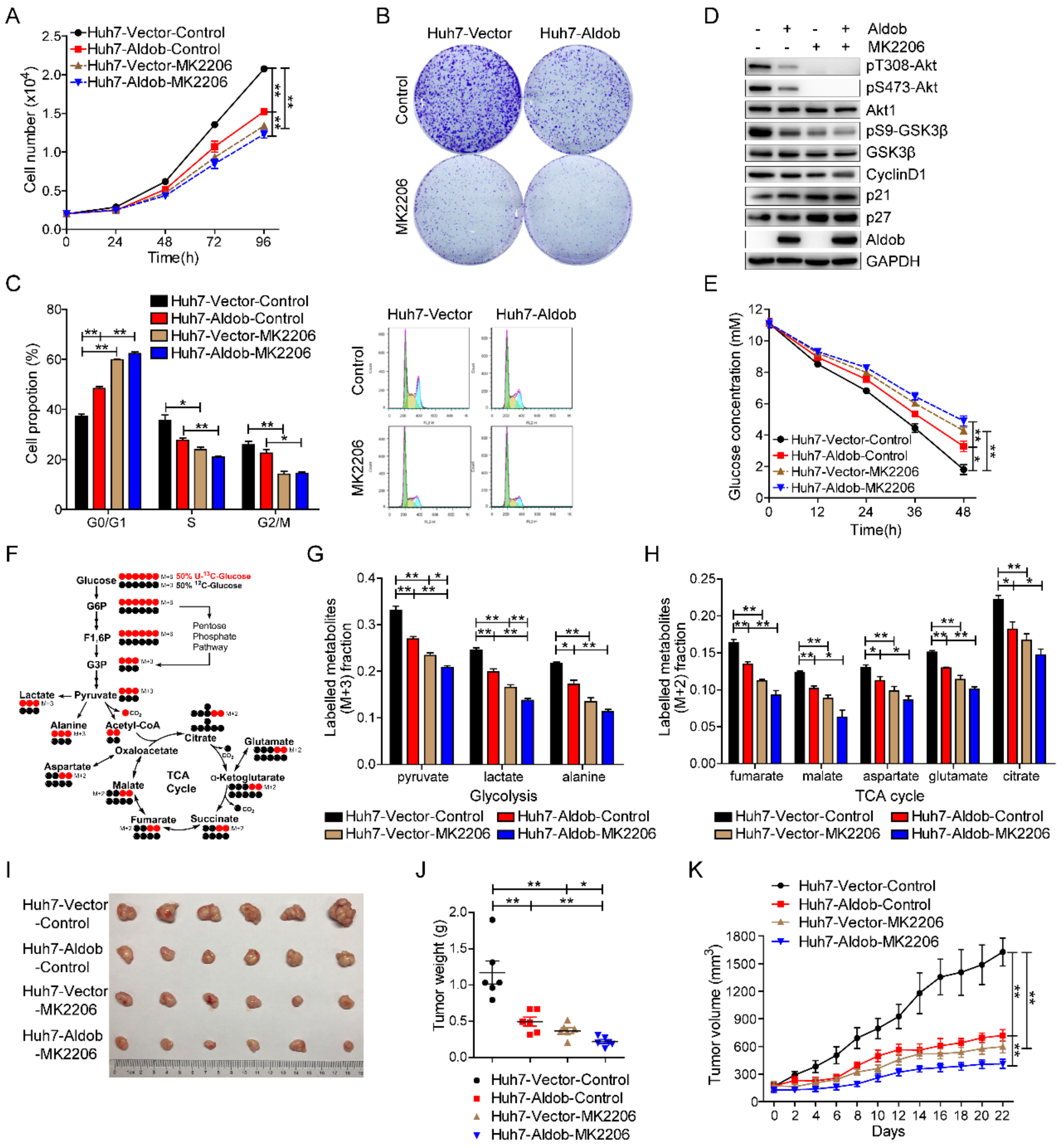

Fig 3. (A) Huh7-Vector and Huh7-Aldob cells were treated with DMSO or MK2206 (2 µM) at the indicated time and cell viability was measured by CCK-8 assays. **(B)** Huh7-Vector and Huh7-Aldob cells (5000 cells/well in 6-well plates) were cultured with or without MK2206 (2 µM) for 10 days before being fixed and stained. Representative graphs of cell colonies were shown. **(C and D)** Cells were treated with DMSO or MK2206 (2 µM) for 48 hours, and then monitored for cell cycle distribution by FACS **(C)** or subjected to IB analysis with the indicated antibodies **(D)**. **(E)** Glucose levels in the culture medium of Huh7-Vector and Huh7-Aldob cells after treatment with DMSO or MK2206 (5 µM) at different time points. **(F)** Schematic presentation of ^{13}C distribution in glycolysis and the first turn of the TCA cycle with 50% U-^{13}C-glucose (labeled at all six carbons, red circles) and 50% ^{12}C-glucose (unlabeled, black circles). G6P, glucose-6-phosphate; F1,6P, fructose-1,6-bisphosphate; G3P, glyceraldehyde-3-phosphate. (**G** and **H**) Fraction of the labeled metabolites of M+3 from ^{13}C-glucose in glycolysis **(G)** and fraction of the labeled metabolites of M+2 from ^{13}C-glucose in TCA cycle **(H)** by DMSO or MK2206 (5 µM) treatment for 12 hours in Huh7-Vector and Huh7-Aldob cells. **(I-K)** Huh7-Vector and Huh7-Aldob cells were injected subcutaneously into the flanks of nude mice, followed by treatment with Control solvent or MK2206 (n=6 mice).

Representative tumor images **(I)**, xenograft tumor weight **(J)** and tumor size **(K)** in each group were recorded. Data are presented as mean ± SEM. * p < 0.05; ** p < 0.01 (Student's t-test).

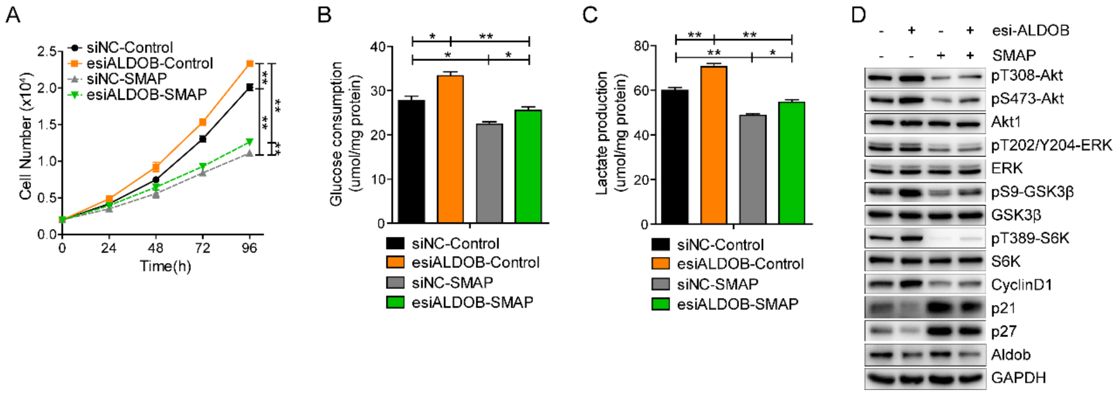

Fig 7. (A-D) Huh7 cells were transfected with esiRNA for knockdown of endogenous Aldob, and cell proliferation **(A)**, glucose consumption **(B)**, lactate production **(C)** and the expression levels of Akt signaling pathway factors **(D)** were determined in the presence of control DMSO or SMAP (10 μM).

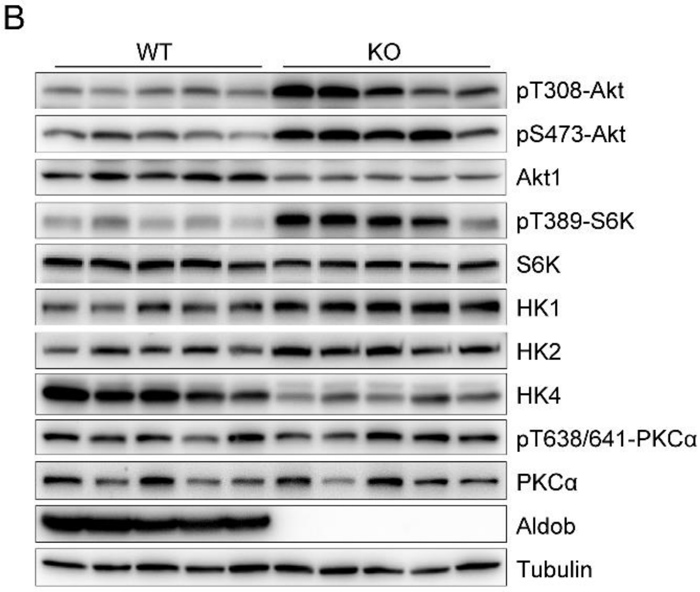

S1 Fig. (B) IB analysis of WCL derived from liver tumor tissues of WT and *ALDOB* KO mice after injection with DEN at post-natal day 14 to induce hepatocellular carcinoma for 10 months.

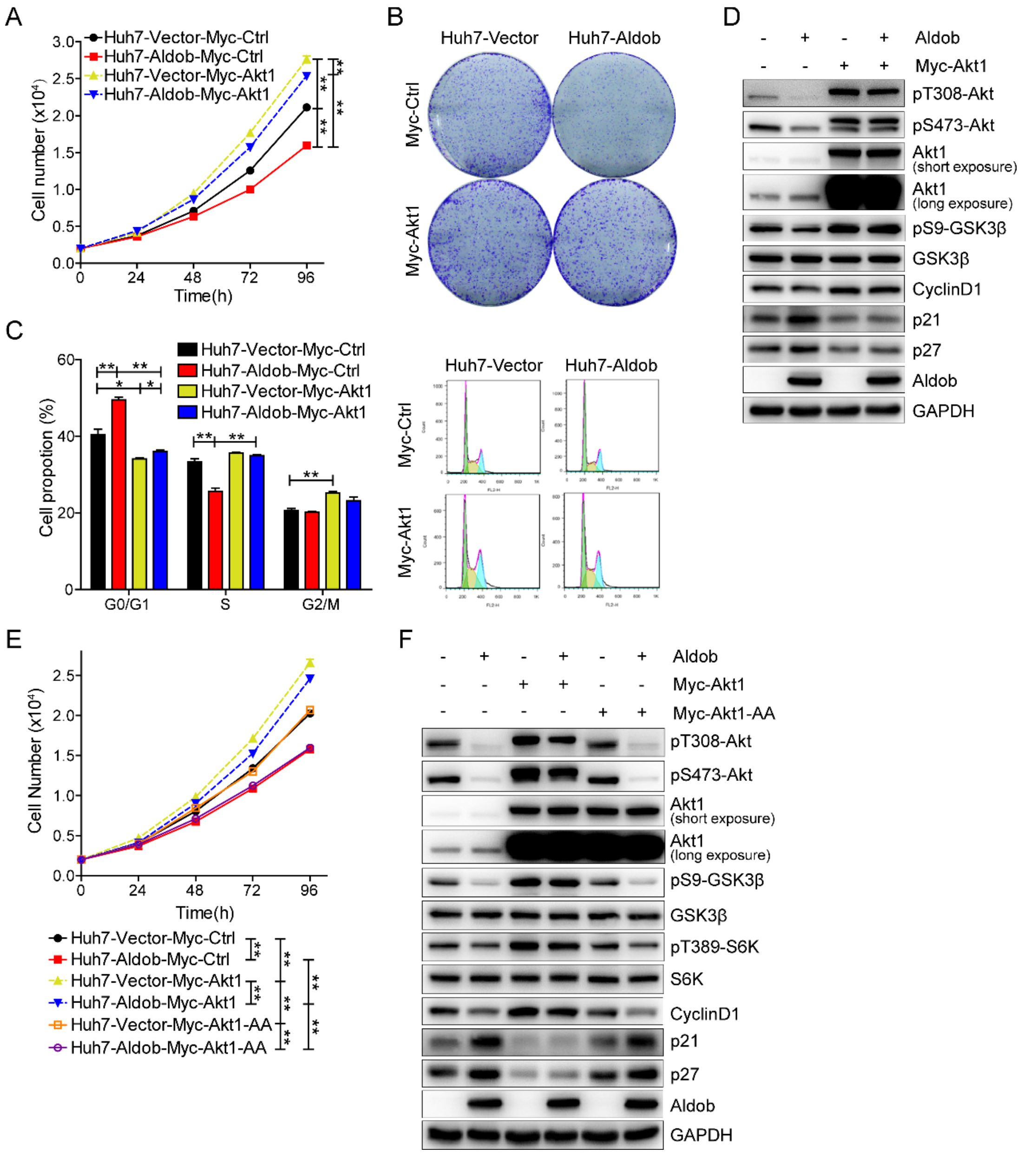

S2 Fig. (A) Cell viability assay of Huh7-Vector and Huh7-Aldob cells transfected with Myc-Ctrl or Myc-Akt1 constructs. **(B)** Representative graphs from colony formation assay of Huh7-Vector and Huh7-Aldob cells after transfection with Myc-Ctrl or Myc-Akt1 for 7 days. **(C** and **D)** Cells were transfected with Myc-Ctrl or Myc-Akt1 for 48 hours, and then monitored for cell cycle distribution **(C)** or subjected to IB analysis **(D)**. **(E)** Cell viability assay of Huh7-Vector and Huh7-Aldob cells transfected with the indicated constructs. Myc-Akt1-AA indicated the Myc-Akt1 phospho-deficient mutant harboring duple mutations (T308A/S473A). **(F)** IB analysis of WCL derived from Huh7 cells transfected with the indicated constructs. Data are presented as mean ± SEM. * $p < 0.05$; ** $p < 0.01$ (Student's t-test).

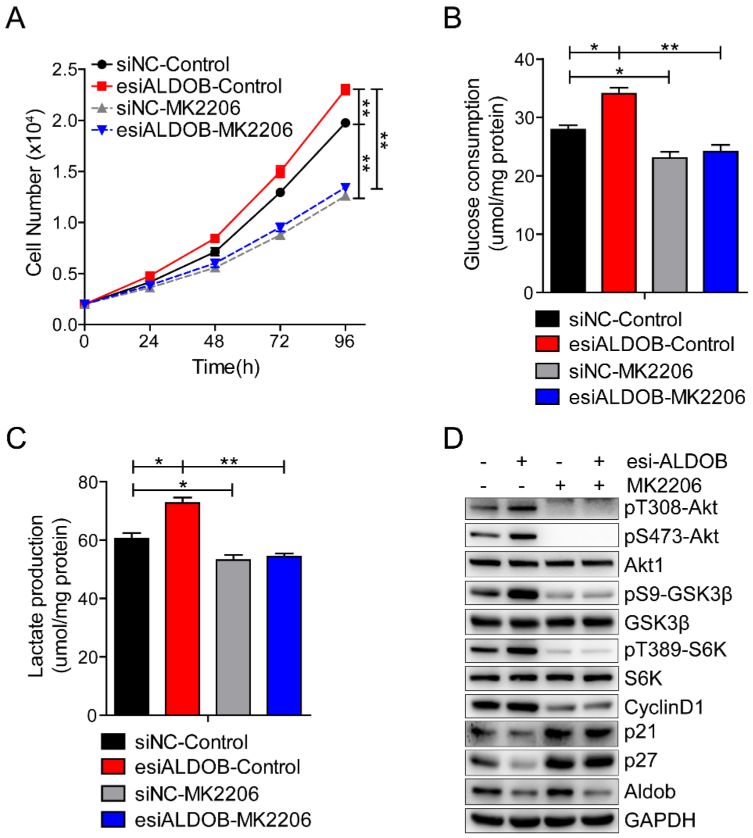

S3 Fig. (A) Cell viability of Huh7 cells transfected with indicated siRNAs in the presence of DMSO or MK2206 (2 μM). **(B and C)** Glucose consumption **(B)** and lactate production **(C)** of Huh7 cells transfected with indicated siRNAs after treatment with DMSO or MK2206 (5 μM). **(D)** IB analysis of WCL derived from Huh7 cells transfected with indicated siRNAs in the presence of DMSO or MK2206 (2 μM).

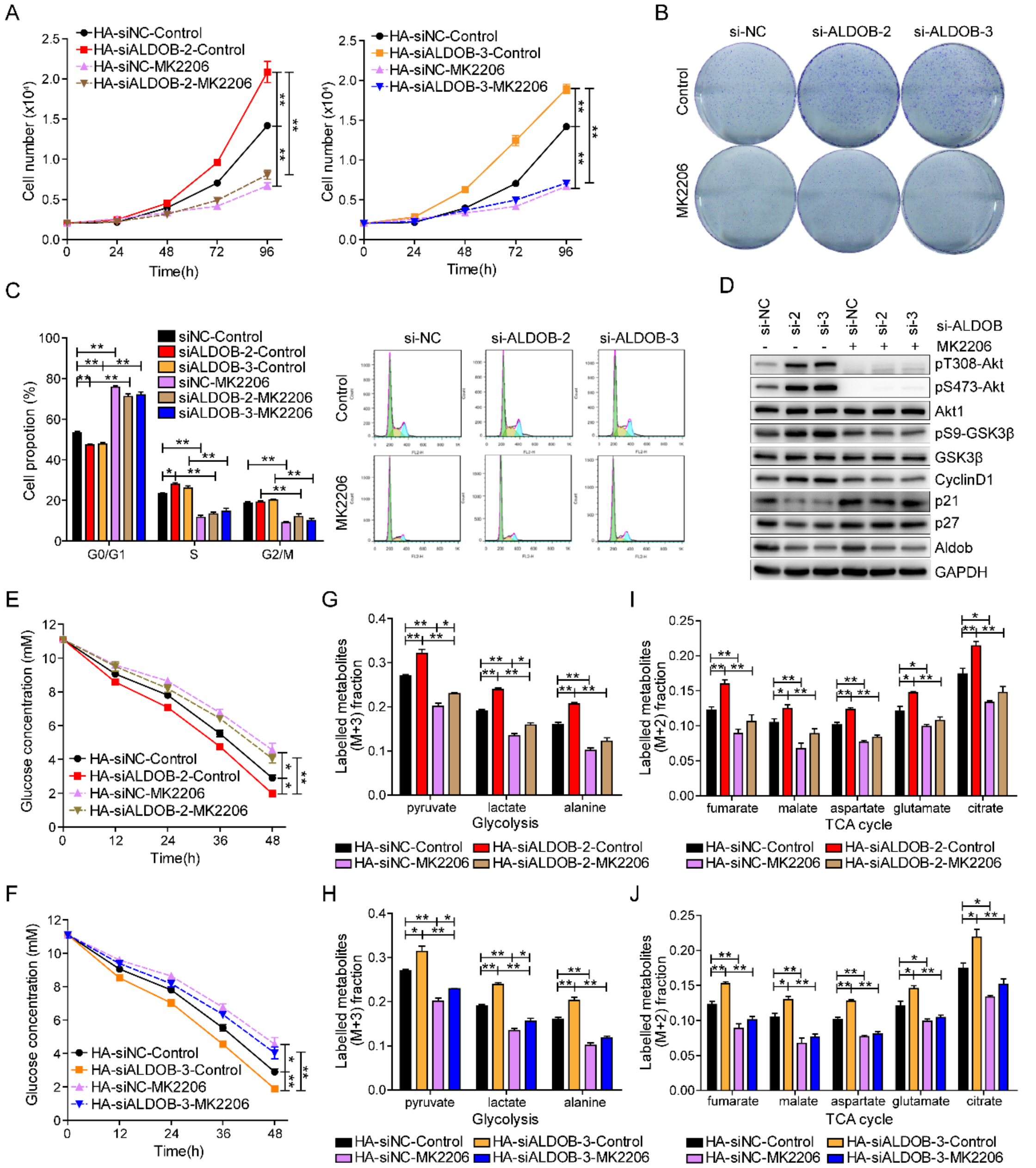

S4 Fig. (A-D) Aldob-overexpressing Huh7 cells transfected with indicated siRNAs were used to determine their effects with or without MK2206 treatment (2 μM) on cell proliferation **(A)**, colony formation **(B)**, cell cycle distribution **(C)** and cell cycle-related protein levels **(D)**. **(E** and **F)** Glucose levels in the culture medium of Huh7-Aldob cells transfected with indicated siRNAs after treatment with DMSO or MK2206 (5 μM) at different time points. **(G** and **H)** Fraction of the labeled metabolites of M+3 from ^{13}C-glucose in glycolysis by DMSO or MK2206 (5 μM) treatment for 12 hours in Huh7-Aldob cells transfected with indicated siRNAs. (**I** and **J**) Fraction of the labeled metabolites of M+2 from ^{13}C-glucose in TCA cycle by DMSO or MK2206 (5 μM) treatment for 12 hours in Huh7-Aldob cells transfected with indicated siRNAs. Data are presented as mean ± SEM. * $p < 0.05$; ** $p < 0.01$ (Student's t-test).

6- To really demonstrate the proposed mechanism, the authors should express mutants

of Akt which are constitutive active and/or cannot be phosphorylated and check whether they become resistant to aldolase signalling.

Response: We thank the reviewer for this suggestion. In ***S2E and F Fig***, Aldob overexpression significantly inhibited cell viability and Akt signaling, as evidenced by reduced expression levels of pT308-Akt, pS473-Akt, pS9-GSK3β, pT389-S6K1 and CyclinD1, as well as increased levels of cell cycle inhibitors p21 and p27. To further determine the involvement of Akt, activation of Akt signaling by ectopic expression of Myc-Akt1 significantly enhanced cell proliferation and rescued Aldob overexpression-induced suppression of cell viability and Akt signaling. However, coexpression of Aldob with Myc-Akt1 ***phospho-deficient mutant*** (Myc-Akt1-AA) failed to diminish the Aldob-mediated above-mentioned tumor-suppressive effects. All these data are consistent with our previous conclusion that Aldob suppresses HCC cell proliferation through inhibition of Akt signaling. We included these data in the ***S2E and F Fig***, and revised the text accordingly.

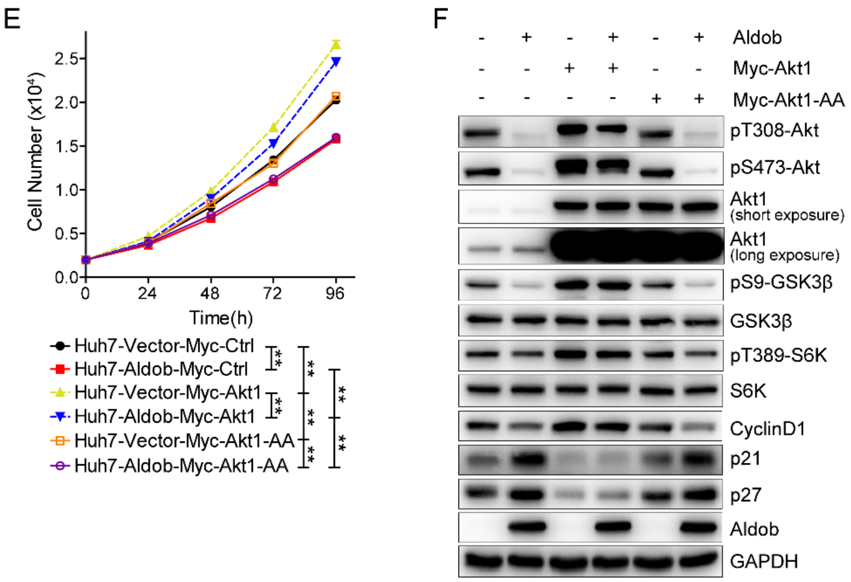

S2 Fig. (E) Cell viability assay of Huh7-Vector and Huh7-Aldob cells transfected with the indicated constructs. Myc-Akt1-AA indicated the Myc-Akt1 phospho-deficient mutant harboring duple mutations (T308A/S473A). **(F)** IB analysis of WCL derived from Huh7 cells transfected with the indicated constructs.

Reference

1. Chang YC, Yang YC, Tien CP, Yang CJ, Hsiao M. Roles of Aldolase Family Genes in Human Cancers and Diseases. Trends in endocrinology and metabolism: TEM. 2018;29(8):549-559.

2. Hamaguchi T, Iizuka N, Tsunedomi R, Hamamoto Y, Miyamoto T, Iida M, et al. Glycolysis module activated by hypoxia-inducible factor 1alpha is related to the aggressive phenotype of hepatocellular carcinoma. International journal of oncology. 2008;33(4):725-731.

3. Li X, Jiang F, Ge Z, Chen B, Yu J, Xin M, et al. Fructose-Bisphosphate Aldolase A Regulates Hypoxic Adaptation in Hepatocellular Carcinoma and Involved with Tumor Malignancy. Digestive diseases and sciences. 2019.

4. He TL, Zheng KL, Li G, Song B, Zhang YJ. Identification of typical miRNAs and target genes in hepatocellular carcinoma by DNA microarray technique. European review for medical and pharmacological sciences. 2014;18(1):108-116.

5. Gao Q, Zhu H, Dong L, Shi W, Chen R, Song Z, et al. Integrated Proteogenomic Characterization of HBV-Related Hepatocellular Carcinoma. Cell. 2019;179(5):1240.

6. Li M, He X, Guo W, Yu H, Zhang S, Wang N, et al. Aldolase B suppresses hepatocellular carcinogenesis by inhibiting G6PD and pentose phosphate pathways. Nature cancer. 2020;1(7):735-747.

7. Manning BD, Toker A. AKT/PKB Signaling: Navigating the Network. Cell. 2017;169(3):381-405.

8. Jang M, Kim SS, Lee J. Cancer cell metabolism: implications for therapeutic targets. Experimental & molecular medicine. 2013;45:e45.

9. DeWaal D, Nogueira V, Terry AR, Patra KC, Jeon SM, Guzman G, et al. Hexokinase-2 depletion inhibits glycolysis and induces oxidative phosphorylation in hepatocellular carcinoma and sensitizes to metformin. Nature communications. 2018;9(1):446.

10. Robey RB, Hay N. Mitochondrial hexokinases, novel mediators of the

antiapoptotic effects of growth factors and Akt. Oncogene. 2006;25(34):4683-4696.
11. Wilson JE. Isozymes of mammalian hexokinase: structure, subcellular localization and metabolic function. The Journal of experimental biology. 2003;206(Pt 12):2049-2057.
12. Cairns RA, Harris IS, Mak TW. Regulation of cancer cell metabolism. Nature reviews Cancer. 2011;11(2):85-95.
13. Lincet H, Icard P. How do glycolytic enzymes favour cancer cell proliferation by nonmetabolic functions? Oncogene. 2015;34(29):3751-3759.
14. Hua S, Lei L, Deng L, Weng X, Liu C, Qi X, et al. miR-139-5p inhibits aerobic glycolysis, cell proliferation, migration, and invasion in hepatocellular carcinoma via a reciprocal regulatory interaction with ETS1. Oncogene. 2018;37(12):1624-1636.
15. Zhou Y, Liu K, Liu Y, Tan L. MicroRNA-34a inhibit hepatocellular carcinoma progression by repressing hexokinase-1. Journal of cellular biochemistry. 2018.
16. Janssens V, Goris J. Protein phosphatase 2A: a highly regulated family of serine/threonine phosphatases implicated in cell growth and signalling. The Biochemical journal. 2001;353(Pt 3):417-439.
17. Slupe AM, Merrill RA, Strack S. Determinants for Substrate Specificity of Protein Phosphatase 2A. Enzyme research. 2011;2011:398751.
18. Saxton RA, Sabatini DM. mTOR Signaling in Growth, Metabolism, and Disease. Cell. 2017;169(2):361-371.
19. Fruman DA, Chiu H, Hopkins BD, Bagrodia S, Cantley LC, Abraham RT. The PI3K Pathway in Human Disease. Cell. 2017;170(4):605-635.
20. Ma L, Chen Z, Erdjument-Bromage H, Tempst P, Pandolfi PP. Phosphorylation and functional inactivation of TSC2 by Erk implications for tuberous sclerosis and cancer pathogenesis. Cell. 2005;121(2):179-193.
21. Zhang CS, Hawley SA, Zong Y, Li MQ, Wang ZC, Gray A, et al. Fructose-1,6-bisphosphate and aldolase mediate glucose sensing by AMPK. Nature. 2017;548(7665):112-+.
22. Li M, Zhang CS, Zong Y, Feng JW, Ma T, Hu M, et al. Transient Receptor Potential

V Channels Are Essential for Glucose Sensing by Aldolase and AMPK. Cell Metab. 2019.
23. Hu H, Juvekar A, Lyssiotis CA, Lien EC, Albeck JG, Oh D, et al. Phosphoinositide 3-Kinase Regulates Glycolysis through Mobilization of Aldolase from the Actin Cytoskeleton. Cell. 2016;164(3):433-446.
24. Tao QF, Yuan SX, Yang F, Yang S, Yang Y, Yuan JH, et al. Aldolase B inhibits metastasis through Ten-Eleven Translocation 1 and serves as a prognostic biomarker in hepatocellular carcinoma. Mol Cancer. 2015;14:170.
