# Supplementary material for: Loss of hepatic aldolase B activates Akt and promotes hepatocellular carcinogenesis by destabilizing the Aldob/Akt/PP2A protein complex
Source: PLoS Biol. 2020 Dec 4;18(12):e3000803. doi: 10.1371/journal.pbio.3000803 (PMC7744066; doi:10.1371/journal.pbio.3000803)
Supplement: Attachment — Submitted filename: PBIOLOGY-D-20-01535R2_Response to Reviewers.pdf [file pbio.3000803.s014.pdf]

中国科学院上海营养与健康研究所
SHANGHAI INSTITUTE OF NUTRITION AND HEALTH, CAS

October 29, 2020

Roland G Roberts, PhD,

Senior Editor, PLOS BIOLOGY

Dear Dr. Roberts,

Thank you so much for tentatively accepting our manuscript entitled "**Loss of hepatic aldolase B activates Akt and promotes hepatocellular carcinogenesis by destabilizing the Aldob/Akt/PP2A protein complex**" (PBIOLOGY-D-20-01535R2). We have renamed the two supplementary data files as "S1_Data" and "S2_Data", and cited them as the location of the underlying data in all of the relevant Figure legends. In addition, we revised the title of our manuscript to "Loss of hepatic aldolase B activates Akt and promotes hepatocellular carcinogenesis by destabilizing the Aldob/Akt/PP2A protein complex".

In summary, we really appreciate the opportunity to revise our manuscript and sincerely thank the reviewers for their comments and suggestions. We hope that our revised manuscript will soon be officially accepted by *PLOS BIOLOGY*.

Sincerely,

Huiyong Yin, Ph.D., Principal Investigator and Professor

Shanghai Institute of Nutrition and Health
Chinese Academy of Sciences.
320 Yueyang Road, Shanghai, China 200031.
Phone: (86)-21-54920942; E-mail: hyyin@sibs.ac.cn
